# Efficacy of transarterial therapy combined with first-line tyrosine kinase inhibitors for unresectable hepatocellular carcinoma: a network meta-analysis

**DOI:** 10.1186/s12957-023-03098-3

**Published:** 2023-07-21

**Authors:** Lingbo Hu, Jiangying Lin, Xingpeng Shi, Aidong Wang

**Affiliations:** 1 Department of Hepatopancreatobiliary Surgery, Taizhou Hospital of Zhejiang Province Affiliated to Wenzhou Medical University, Zhejiang, China; 2 Department of Hepatopancreatobiliary Surgery, Enze Hospital, Taizhou Enze Medical Center (Group), Zhejiang, China; 3 Department of Blood Purification, Taizhou Hospital of Zhejiang Province Affiliated to Wenzhou Medical University, Zhejiang, China

**Keywords:** Transarterial chemoembolization, Hepatic arterial infusion chemotherapy, Selective internal radiation therapy, Tyrosine kinase inhibitors, Hepatocellular carcinoma, Network meta-analysis

## Abstract

**Background:**

Transarterial therapies, including transarterial chemoembolization (TACE), hepatic arterial infusion chemotherapy (HAIC), and selective internal radiation therapy, combined with first-line tyrosine kinase inhibitors (TKIs) are considered the standard therapy for unresectable hepatocellular carcinoma. However, inconsistent results have been reported in various studies assessing different combinations of targeted agents.

**Methods:**

A network meta-analysis (NMA) was performed by including 23 randomized controlled trials (RCTs) with 6175 patients to investigate the efficiency of transarterial therapies in combination with different TKIs. Outcomes of interest included overall survival (OS), progression-free survival (PFS), time to progression (TTP), and tumor objective response rate (ORR). A random-effects consistency model was used in this Bayesian NMA. Hazard ratio and odd risks with a 95% credible interval were calculated and agents were ranked based on ranking probability.

**Results:**

HAIC showed maximal OS and TTP and TACE plus lenvatinib showed maximal PFS, ORR, and disease control rate (DCR). HAIC and TACE plus lenvatinib were ranked highest based on their respective parameters, which were OS for HAIC and PFS, ORR, and DCR for TACE plus lenvatinib.

**Conclusion:**

HAIC and TACE plus lenvatinib were relatively better choice for unresectable hepatocellular carcinoma. However, owing to the lack of statistically significant OS benefits among most agents, other agents should be considered as potential alternatives for unresectable hepatocellular carcinoma.

**Supplementary Information:**

The online version contains supplementary material available at 10.1186/s12957-023-03098-3.

## Introduction

Liver cancer is the sixth most common cancer and the third most common cause of cancer-related mortality worldwide. Hepatocellular carcinoma (HCC) is the most common subtype of liver cancer [1]. Liver resection, radiofrequency ablation, and liver transplantation have been recommended as curative treatments by the European Association for the Study of the Liver (EASL) and the American Association for the Study of Liver Diseases (AASLD) guidelines [2, 3]. However, less than 30% of the patients have the chance to receive curative treatments because the disease in more than 70% of the patients is diagnosed at a medium or advanced stage, which makes the option of surgery unfeasible [4, 5]. The outcome for these patients with advanced HCC is dismal, with a 5-year survival rate of only 5–36% [6].

For the treatment of advanced HCC, transarterial therapies, immune checkpoint inhibitors, and tyrosine kinase inhibitors (TKIs) are most commonly used [7, 8]. Transarterial therapies include transarterial chemoembolization (TACE), hepatic arterial infusion chemotherapy (HAIC), and transarterial radiation embolization (TARE), which is also called selective internal radiation therapy (SIRT). The first-line TKIs include drugs such as sorafenib, lenvatinib, and donafenib.

About 20 years ago, many meta-analyses, including high-quality randomized controlled trials (RCTs), showed that TACE is an effective and safe therapy for patients with intermediate-stage HCC compared with the best supportive care or tamoxifen [9, 10]. Based on these studies, TACE has been recommended by many guidelines and adopted as a standard of care for patients with intermediate-stage HCC globally [2, 3]. Furthermore, many clinicians have used TACE for treating patients with advanced HCC concomitant with portal vein tumor thrombosis (PVTT); however, the positive therapeutic outcomes have been limited [11, 12]. TACE combined with sorafenib was also used to treat patients with advanced HCC [13, 14]. The subtotal abdominal hysterectomy (STAH) and transcatheter arterial chemoembolization therapy in combination with sorafenib (TACTICS) trials showed that the combination of sorafenib and TACE significantly improved the progression-free survival (PFS) rate [13, 14].

HAIC is a treatment that delivers a high concentration of a local chemotherapeutic drug continuously through the hepatic artery. The Chinese and Japanese guidelines recommend HAIC as the preferred treatment for HCC with PVTT [15, 16]. Compared with sorafenib, HAIC has shown remarkable therapeutic effects, such as better overall survival (OS) rate, time to progression (TTP), and objective response rate (ORR), for patients with advanced HCC concomitant with PVTT [17, 18]. Furthermore, the HAIC treatment provided better effectiveness and fewer adverse effects for patients with large HCC compared with the TACE treatment [19].

SIRT, which is also known as TARE, is a treatment that delivers nonbiodegradable microspheres loaded with yttrium-90, instead of the chemotherapeutic drug, to the hepatic artery that nourishes the tumor. In retrospective studies, the treatment of patients with unresectable HCC from Barcelona Clinic Liver Cancer (BCLC) stage A to stage C with SIRT provided robust evidence of the survival benefit and excellent disease control [20, 21]. AASLD and EASL guidelines recommend SIRT to patients with intermediate-stage HCC with level 2 evidence [2, 3]. However, in three separately performed RCTs, SIRT did not show any advantages over sorafenib for treating advanced HCC [22–24].

Sorafenib is the first oral multikinase inhibitor that can improve OS in patients with advanced HCC. In the SHARP trial, sorafenib improved OS by 2.3 months compared with the placebo [25]. A similar effect was seen in another study with Asia–Pacific patients with advanced HCC [26]. Lenvatinib is another multikinase inhibitor that is less effective than sorafenib in terms of improving OS in patients with advanced HCC [27]. However, lenvatinib is advantageous in several aspects, such as improving PFS, TTP, and ORR in patients with advanced HCC. Donafenib is a novel multikinase inhibitor and a deuterated sorafenib derivative, which is more advantageous than sorafenib in improving OS of advanced HCC patients and has favorable safety and tolerability [28].

Despite these effective treatment options, the extent of improvement in OS is limited. Therefore, the combinations of different monotherapies were explored, such as atezolizumab plus bevacizumab [29], sintilimab plus a bevacizumab biosimilar [30], TACE combined with sorafenib [13, 14, 31–35], TACE combined with lenvatinib [36, 37], HAIC combined with sorafenib [38–42], and SIRT combined with sorafenib [22, 23, 43, 44]. Some combinations showed an exhilarating effect on OS, whereas the effects of some were the same as those of the monotherapies.

There is a lack of consensus about which regimen is most effective among the combination and monotherapy regimens because of limited head-to-head studies. Therefore, a network meta-analysis (NMA) was considered the best way to address this concern. In this study, we performed an NMA of transarterial therapy, first-line TKIs, and their combinations in the treatment of patients with unresectable HCC and ranked the therapies based on their treatment effects.

## Method

In this study, NMA was performed using the Preferred Reporting Items for Systematic Reviews and Meta-Analyses (PRISMA) Extension Statement, referred to as PRISMA-NMA [45]. The proposed review is registered in PROSPERO (registration no. CRD 42021266287).

### Eligibility criteria for inclusion of RCTs

All published RCTs that compared the use of transarterial therapy (i.e., TACE, HACI, and SIRT), TKI (i.e., sorafenib, lenvatinib, and donafenib), or a combination of them for the treatment of unresectable HCC were included. Studies comparing other combination therapies were excluded.

### Outcomes of interest

The efficacy of the therapies was determined based on different outcomes of interest. The primary efficacy outcome was OS. The second efficacy outcomes included PFS, TTP, ORR, and disease control rate (DCR), which were evaluated by either Response Evaluation Criteria In Solid Tumors (RECIST) or modified Response Evaluation Criteria In Solid Tumors (mRECIST) [46, 47]. The safety outcomes were treatment-related grade 3 or grade 4 adverse effects (AEs). OS, PFS, and TTP were measured using the hazard ratio (HR). Engauge Digitizer version 4.1 was used to obtain the HR with a 95% confidence interval (CI) from the survivorship curve [48], following the method described by Tierney et al., when HR was not available in the reported studies [49]. The odds ratio (OR) with 95% CI was calculated for ORR, DCR, and AE.

### Definition

OS was calculated from the date of random assignment to the date of death from any cause or the date of the last follow-up. PFS was defined as the interval from random assignment to progression according to RECIST or mRECIST critieria or death from any cause. TTP was defined as time to recurrence in patients with complete response or progression in patients without complete response. ORR was defined as the percentage of patients achieving either complete response (CR) or partial response (PR), and DCR as the percentage of patients achieving CR, PR, or stable disease (SD) according to RECIST or mRECIST critieria, and AEs by the use of Common Terminology Criteria for Adverse Events v4.0. We only calculated grade 3 and 4 AEs in our study.

### Data source and extraction

Comprehensive searches were carried out on PubMed, Embase, Web of Science, and the Cochrane library from inception until December 12, 2021 (Supplementary material S1). The last search was updated in November 2022. The subject words and keywords include hepatocellular carcinoma, transarterial chemoembolization, hepatic arterial infusion therapy, selective internal radiation therapy, sorafenib, lenvatinib, and dorafenib were used. The literature retrieval was independently carried out. The study language was restricted to English. Unpublished studies were not searched and experts in the field were not contacted. All the data were retrieved from the qualified published RCTs.

Data regarding the following were independently extracted and later compiled from the included trials: published year, participant’s age, gender, ECOG status, Child–Pugh, interventions, tumor characteristics, and primary and secondary outcomes.

### Risk of bias and quality of evidence assessments

The potential biases for each study were summarized using the Cochrane risk of bias tool [50].

### Statistical analysis

The Bayesian approach was adopted in this network meta-analysis using a random-effects model with the package gemtc in the R software version 4.1.2 (R Project for Statistical Computing). Network plots showing an indirect comparative relationship among different interventions were constructed using the Stata software (version 15.1, Stata Corporation, College Station, TX, USA). The funnel plots of the outcome indicators with Egger’s test were plotted to describe the publication bias using the package netmeta in the R software. OS, PFS, and TTP were estimated using HR with 95% credible interval (CrI). For ORR, DCR, and AE, OR with 95% CrI was calculated. *P* values < 0.05 were considered statistically significant. Convergence was determined using Brooks–Gelman–Rubin diagnostics, traces, and density plots. A total of 50,000 iterations were performed, of which the initial 20,000 were used to anneal the algorithm for removing the effect of the initial value. Forest plots were mapped to compare the results. The ranking probabilities of the different treatments were estimated by the “Rank. Probability” function. The “mtc.anohe” command in the “gemtc” package was used to evaluate heterogeneity, which was determined using the variance parameter *I*^2^. To determine whether the results of the direct and indirect comparisons were consistent within treatment loops, node-splitting models were used to assess the local consistency of NMA [51].

## Results

### Study selection and characteristics of included studies

**Fig. 1:**
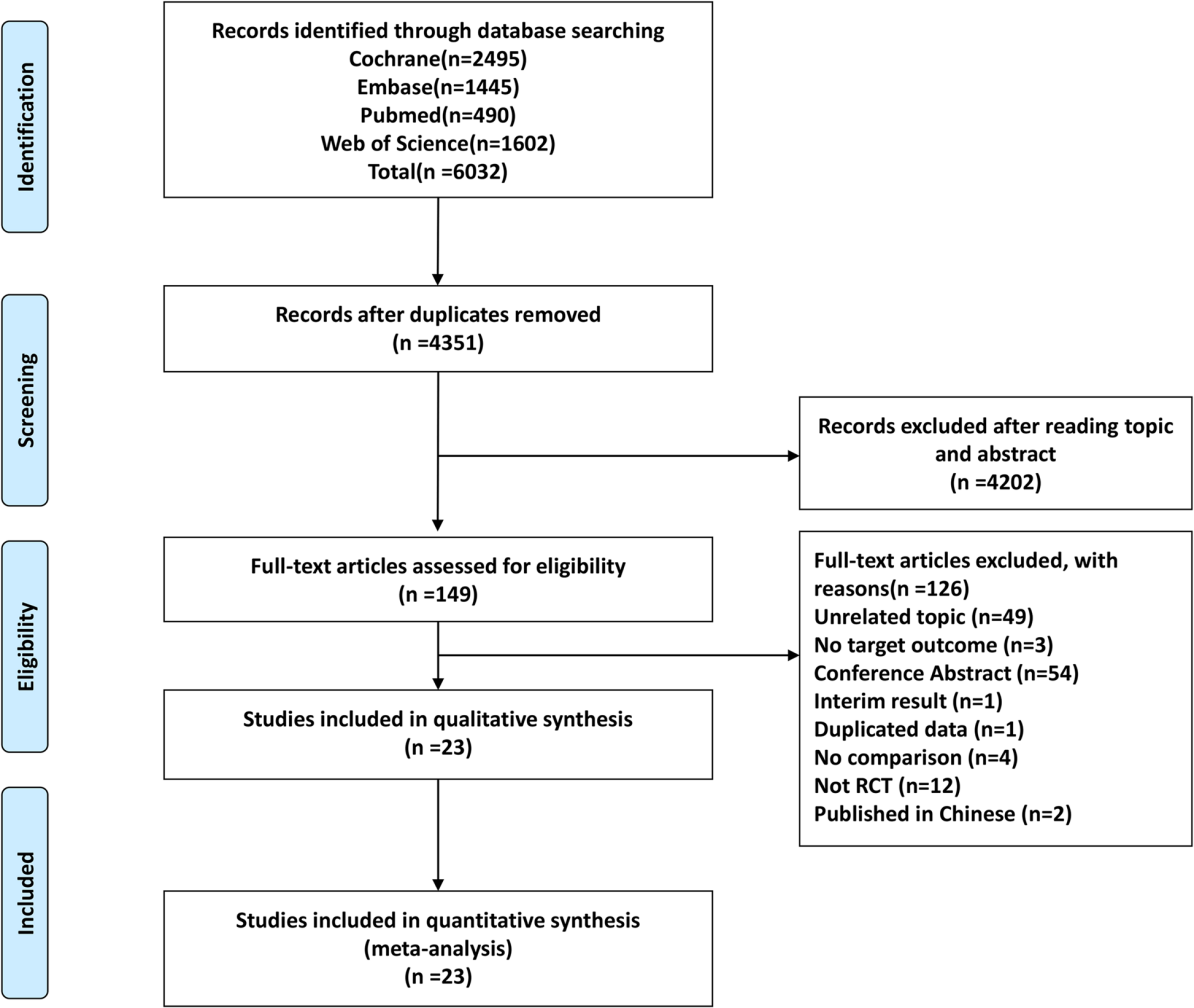
PRISMA flow diagram

Using the search strategy shown in Supplementary Material S1, we identified a total of 6032 titles and abstracts. Of the total, 149 articles fulfilling the eligibility criteria were included for assessment. Among these, 23 RCTs were included in the network meta-analysis [13, 14, 17–19, 22–24, 27, 28, 34, 36, 37, 39–42, 44, 52] after excluding 12 non-RCTs, 54 published abstracts or conference proceedings, 49 unrelated topics, and other articles for reasons shown in the PRISMA flow diagram (Fig. 1). We did not include any additional studies after searching again.

Supplementary Material S2 and S3 detail the baseline characteristics of the included studies. The included 23 trials involved 6175 patients. The direct comparisons included HAIC plus sorafenib vs. sorafenib (five trials), HAIC vs. sorafenib (two trials) or TACE (one trial), TACE plus sorafenib vs. TACE (four trials) or sorafenib (one trial), TACE plus lenvatinib vs. lenvatinib (one trial) or TACE plus sorafenib (one trial), SIRT plus sorafenib vs. sorafenib (one trial), SIRT vs. sorafenib (two trials) or TACE (one trial), lenvatinib vs. sorafenib (one trial), and donafenib vs. sorafenib (one trial). The proportion of patients with Barcelona clinical staging of liver cancer (BCLC) stage B or C was high and that of patients with unresectable BCLC stage A was low. All liver functions were either Child–Pugh A or B.

**Fig. 2:**
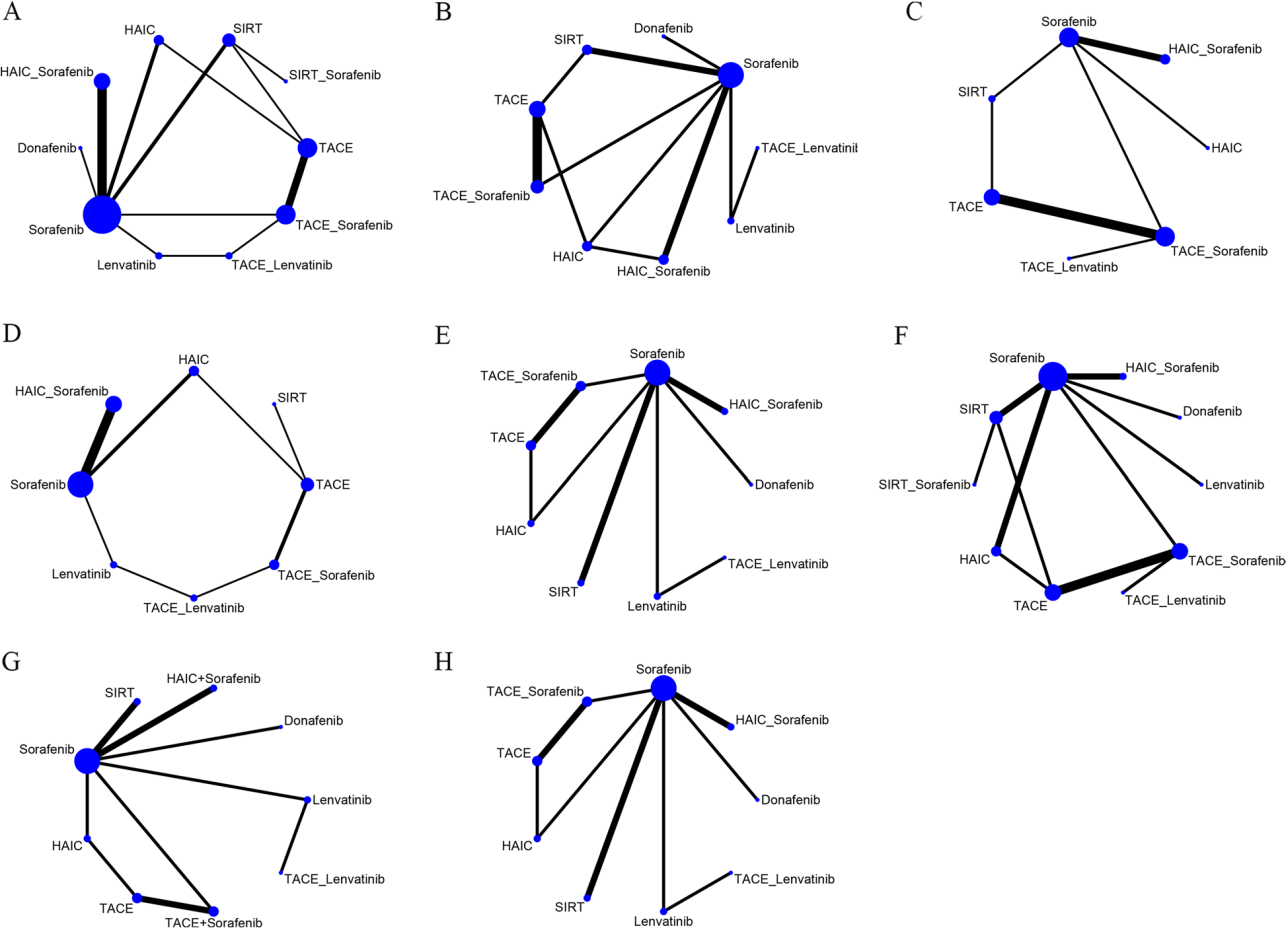
Network graph of the outcomes. **A** Network graph of overall survival. **B** Network graph of progression-free survival. **C** Network graph of time to progression. **D** Network graph of objective response rate evaluated by mRECIST criteria. **E** Network graph of disease controlled rate evaluated by mRECIST criteria. **F** Network graph of adverse event. **G** Network graph of objective response rate evaluated by RECIST criteria. **H** Network graph of disease controlled rate evaluated by RECIST criteria

Figure 2 depicts the treatment network; the thickness of each line of the plots in the network is proportional to the number of comparisons. Owing to the different proportions of BCLC stage B and C patients enrolled in different studies, interstudy heterogeneity was inevitable. Therefore, a random-effects model was used in the analysis.

### OS

**Fig. 3:**
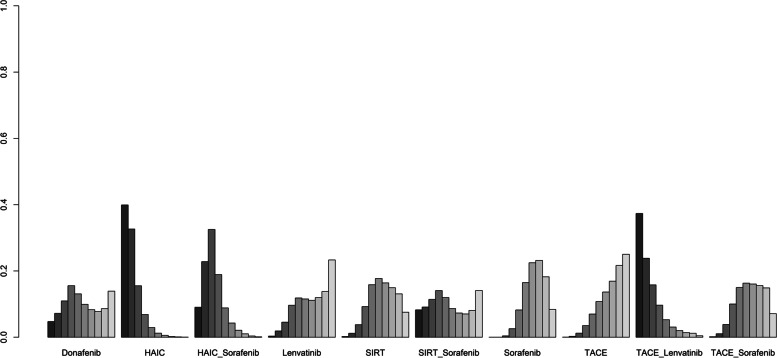
Ranking probabilities of OS in the studies. The dark-to-light color indicates the order of the rank. Dark color reflects a better survival. The size of the bar is proportional to the probability of interventions in each treatment

**Fig. 4:**
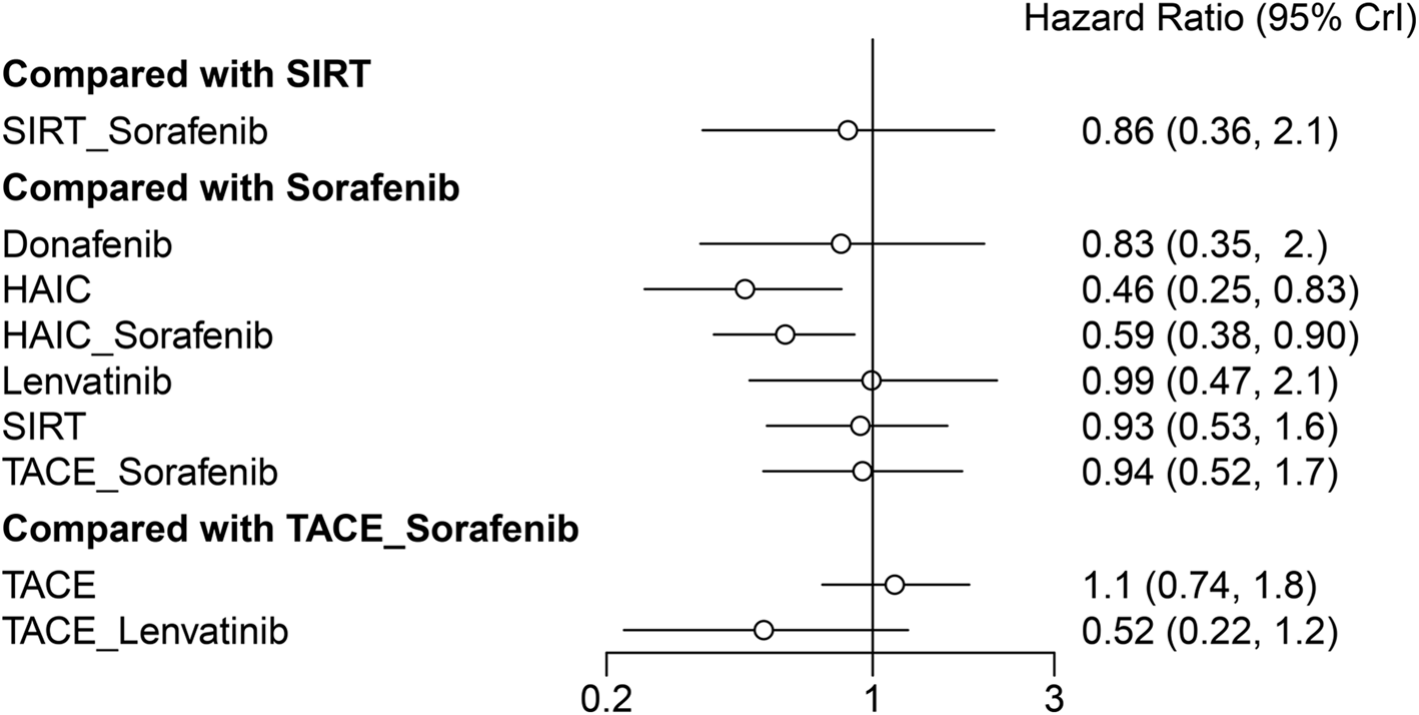
Comparison of OS among different treatments

**Fig. 5:**
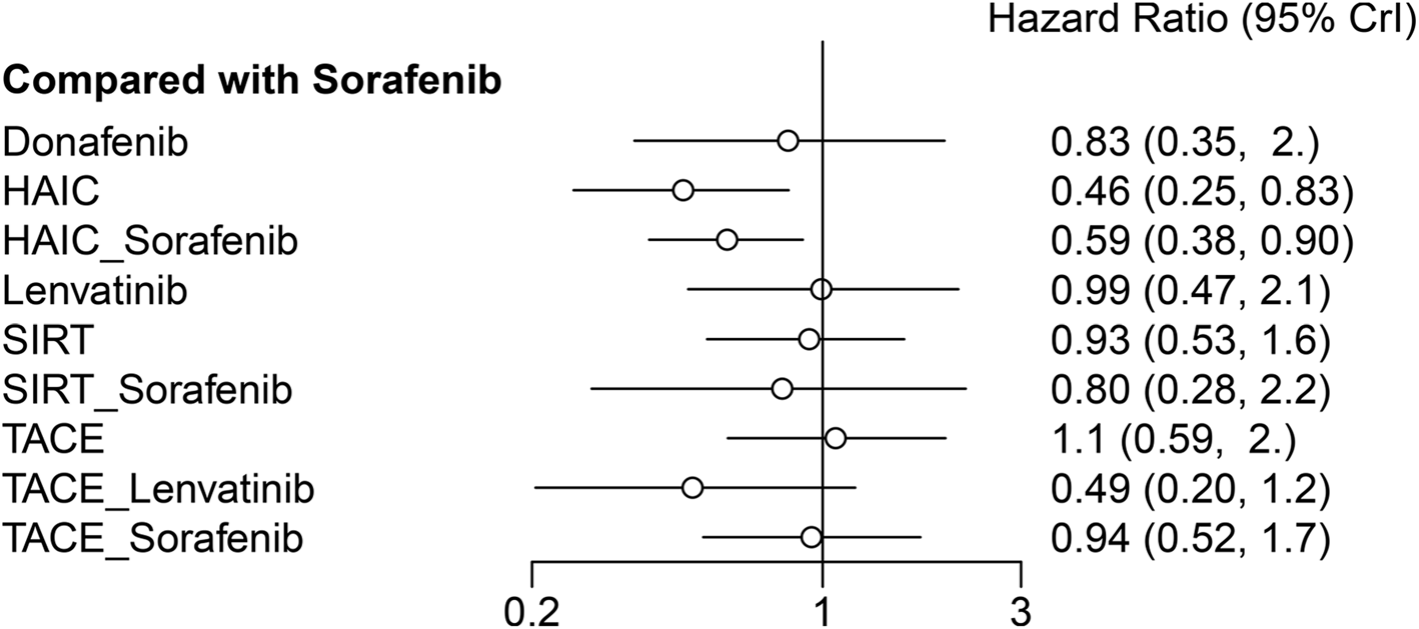
Comparison of OS between sorafenib and other different treatments

The included studies reported ten different treatment modalities to compare OS. The result of the network meta-analysis is presented in Table 1. Compared with sorafenib, HAIC plus sorafenib or HAIC monotherapy showed significantly better OS (HR 0.59, 95% CrI 0.38–0.90; HR 0.46, 95% CrI 0.25–0.83, respectively), whereas other agents showed a similar effect. Furthermore, HAIC monotherapy had significantly better OS than TACE (HR 0.43, 95% CrI 0.22–0.82) or TACE plus sorafenib (HR 49, 95% CrI 0.24–0.99). The treatment ranking analysis indicated that the likelihood of maximal OS was the highest with HAIC, followed by TACE plus lenvatinib and HAIC plus sorafenib (Figs. 3, 4 and 5) and Supplementary Material S4).

**Table 1:** Indirect comparisons of outcomes among different treatments

OS									
Donafenib									
1.79 (0.64,5.21)	HAIC								
1.4 (0.54,3.71)	0.79 (0.38,1.62)	HAIC_Sorafenib							
0.84 (0.26,2.59)	0.46 (0.18,1.15)	0.59 (0.25,1.39)	Lenvatinib						
0.89 (0.33,2.53)	0.5 (0.23,1.07)	0.64 (0.32,1.28)	1.07 (0.44,2.72)	SIRT					
1.03 (0.28,4.06)	0.58 (0.18,1.86)	0.73 (0.24,2.27)	1.25 (0.36,4.53)	1.16 (0.48,2.8)	SIRT_Sorafenib				
0.83 (0.35,1.95)	*0.46 (0.25,0.83)*	*0.59 (0.38,0.9)*	0.99 (0.48,2.12)	0.93 (0.53,1.57)	0.8 (0.28,2.2)	Sorafenib			
0.77 (0.27,2.2)	*0.43 (0.22,0.82)*	0.55 (0.26,1.13)	0.92 (0.38,2.23)	0.86 (0.43,1.67)	0.74 (0.24,2.2)	0.93 (0.51,1.68)	TACE		
1.7 (0.5,5.75)	0.95 (0.34,2.53)	1.21 (0.44,3.19)	2.05 (0.94,4.39)	1.92 (0.68,5)	1.65 (0.42,6.05)	2.05 (0.84,4.9)	2.2 (0.88,5.47)	TACE_Lenvatinib	
0.88 (0.31,2.51)	*0.49 (0.24,0.99)*	0.63 (0.3,1.3)	1.06 (0.45,2.51)	0.99 (0.47,1.97)	0.85 (0.27,2.59)	1.06 (0.58,1.93)	1.14 (0.74,1.79)	0.52 (0.22,1.23)	TACE_Sorafenib
PFS									
TACE_Lenvatinib									
0.79 (0.17, 3.94)	HAIC_Sorafenib								
0.43 (0.15, 1.21)	0.55 (0.16, 1.79)	Lenvatinib							
0.41 (0.08, 1.99)	0.52 (0.22, 1.09)	0.95 (0.26, 3.19)	HAIC						
0.38 (0.07, 1.93)	0.48 (0.18, 1.22)	0.88 (0.24, 3.16)	0.91 (0.4, 2.27)	TACE_Sorafenib					
0.32 (0.05, 1.84)	0.4 (0.12, 1.3)	0.73 (0.17, 3.06)	0.76 (0.23, 2.77)	0.84 (0.23, 3.03)	Donafenib				
0.3 (0.06, 1.46)	*0.38 (0.15, 0.91)*	0.68 (0.2, 2.29)	0.72 (0.3, 1.86)	0.78 (0.31, 1.99)	0.94 (0.28, 3.16)	SIRT			
0.29 (0.07, 1.23)	*0.36 (0.18, 0.68)*	0.66 (0.24, 1.82)	0.7 (0.35, 1.46)	0.76 (0.35, 1.67)	0.9 (0.33, 2.51)	0.96 (0.5, 1.9)	Sorafenib		
0.25 (0.05, 1.26)	*0.31 (0.12, 0.77)*	0.57 (0.15, 2.01)	0.6 (0.27, 1.35)	0.65 (0.36, 1.14)	0.78 (0.21, 2.72)	0.84 (0.33, 1.99)	0.86 (0.39, 1.84)	TACE	
TTP									
HAIC									
0.28 (0.06, 1.19)	HAIC_Sorafenib								
0.33 (0.07, 1.52)	1.15 (0.51, 3)	SIRT							
*0.24 (0.06, 0.92)*	0.84 (0.51, 1.45)	0.73 (0.34, 1.43)	Sorafenib						
*0.19 (0.04, 0.84)*	0.68 (0.25, 1.6)	0.58 (0.23, 1.16)	0.8 (0.34, 1.58)	TACE					
0.53 (0.09, 3.16)	1.92 (0.51, 6.89)	1.65 (0.44, 5.42)	2.25 (0.66, 7.17)	2.83 (1.03, 8.5)	TACE_Lenvatinb				
0.29 (0.06, 1.31)	1.04 (0.42, 2.48)	0.9 (0.37, 1.88)	1.23 (0.58, 2.44)	1.54 (1.05, 2.51)	0.55 (0.21, 1.43)	TACE_Sorafenib			
AE									
SIRT									
0.89 (0.12, 5.53)	HAIC								
0.7 (0.05, 9.97)	0.8 (0.06, 12.81)	Donafenib							
0.64 (0.11, 3.78)	0.73 (0.14, 4.35)	0.9 (0.06, 15.96)	TACE						
0.62 (0.06, 6.05)	0.7 (0.04, 14.3)	0.88 (0.03, 31.19)	0.96 (0.05, 16.78)	SIRT_Sorafenib					
0.43 (0.1, 1.79)	0.49 (0.12, 2.2)	0.61 (0.06, 5.99)	0.67 (0.12, 3.42)	0.7 (0.05, 10.07)	Sorafenib				
0.31 (0.02, 4.48)	0.36 (0.03, 5.42)	0.44 (0.02, 10.59)	0.49 (0.03, 7.61)	0.51 (0.01, 16.78)	0.73 (0.08, 6.89)	Lenvatinib			
0.17 (0.02, 1.49)	0.2 (0.02, 1.84)	0.25 (0.01, 3.86)	0.27 (0.02, 2.61)	0.28 (0.01, 6.3)	0.4 (0.07, 2.08)	0.55 (0.03, 8.58)	HAIC_Sorafenib		
*0.15 (0.02, 0.9)*	0.16 (0.02, 1.07)	0.21 (0.01, 3.13)	*0.23 (0.05, 0.76)*	0.23 (0.01, 4.18)	0.34 (0.05, 1.68)	0.46 (0.02, 6.89)	0.84 (0.07, 8.67)	TACE_Sorafenib	
0.08 (0, 1.57)	0.09 (0, 1.88)	0.11 (0, 4.18)	0.12 (0.01, 1.72)	0.12 (0, 5.16)	0.17 (0.01, 3.19)	0.24 (0.01, 9.21)	0.43 (0.01, 12.81)	0.52 (0.04, 6.05)	TACE_Lenvatinib
ORR mRECIST									
SIRT									
*0 (0, 0.85)*	Sorafenib								
*0 (0, 0.25)*	0.28 (0.04, 1.86)	Lenvatinib							
*0 (0, 0.17)*	0.23 (0.02, 2.14)	0.81 (0.07, 9.39)	TACE						
*0 (0, 0.15)*	0.2 (0.02, 2.12)	0.7 (0.06, 8.17)	0.87 (0.21, 3.71)	TACE_Sorafenib					
*0 (0, 0.14)*	*0.15 (0.05, 0.45)*	0.55 (0.06, 5.16)	0.68 (0.06, 8.94)	0.78 (0.06, 11.47)	HAIC_Sorafenib				
*0 (0, 0.08)*	*0.1 (0.02, 0.49)*	0.37 (0.04, 3.49)	0.45 (0.07, 2.97)	0.52 (0.05, 4.53)	0.66 (0.08, 4.44)	HAIC			
*0 (0, 0.06)*	*0.07 (0.01, 0.7)*	0.24 (0.04, 1.67)	0.3 (0.03, 2.92)	0.35 (0.04, 2.69)	0.44 (0.03, 5.75)	0.67 (0.06, 8.25)	TACE_Lenvatinib		
DCR mRECIST									
Sorafenib									
0.61 (0.06,5.31)	TACE								
0.57 (0.05,5.64)	0.93 (0.23,3.82)	TACE_Sorafenib							
0.52 (0.08,3.25)	0.84 (0.08,10.28)	0.9 (0.08,10.91)	Lenvatinib						
0.53 (0.17,1.52)	0.87 (0.08,10.18)	0.92 (0.07,12.55)	1.02 (0.12,8.58)	HAIC_Sorafenib					
0.25 (0.05,1.02)	0.41 (0.06,2.69)	0.44 (0.05,3.86)	0.49 (0.05,4.06)	0.48 (0.07,2.86)	HAIC				
*0.09 (0.01,0.89)*	0.15 (0.01,1.52)	0.16 (0.02,1.38)	0.17 (0.02,1.17)	0.17 (0.01,2.23)	0.36 (0.03,4.14)	TACE_Lenvatinib			
ORR RECIST									
Sorafenib									
0.59 (0.03,10.8)	Lenvatinib								
0.57 (0.02,15.33)	0.97 (0.01,81.45)	Donafenib							
0.23 (0.02,2.2)	0.39 (0.01,16.12)	0.4 (0.01,21.12)	SIRT						
0.18 (0,14.3)	0.3 (0.01,7.77)	0.31 (0,72.24)	0.79 (0.01,119.1)	TACE_Lenvatinib					
*0.1 (0.01,0.88)*	0.17 (0.01,3.39)	0.18 (0,8.76)	0.44 (0.02,11.25)	0.56 (0.01,45.15)	TACE_Sorafenib				
*0.1 (0.01,0.98)*	0.17 (0.01,3.32)	0.17 (0,9.3)	0.43 (0.02,12.43)	0.54 (0.01,42.95)	0.96 (0.11,7.92)	TACE			
*0.03 (0,0.39)*	0.05 (0,2.59)	0.06 (0,3.56)	0.14 (0,4.57)	0.18 (0,27.94)	0.32 (0.01,8)	0.33 (0.01,9.78)	HAIC_Sorafenib		
*0.03 (0,0.37)*	0.04 (0,1.67)	0.05 (0,3.03)	0.11 (0,4.14)	0.14 (0,18.54)	0.25 (0.01,4.81)	0.26 (0.02,3.6)	0.79 (0.02,35.16)	HAIC	
DCR RECIST									
SIRT									
0.76 (0.29, 2.05)	Sorafenib								
0.7 (0.13, 3.74)	0.9 (0.23, 3.49)	Donafenib							
0.51 (0.12, 2.59)	0.66 (0.21, 2.41)	0.73 (0.13, 5.05)	Lenvatinib						
0.42 (0.11, 1.73)	0.54 (0.2, 1.52)	0.61 (0.12, 3.35)	0.82 (0.22, 2.75)	TACE					
0.38 (0.1, 1.39)	0.49 (0.19, 1.2)	0.54 (0.1, 2.75)	0.73 (0.2, 2.32)	0.9 (0.34, 2.23)	TACE_Sorafenib				
*0.2 (0.05, 0.9)*	*0.26 (0.09, 0.83)*	0.29 (0.05, 1.73)	0.4 (0.08, 1.72)	0.49 (0.16, 1.49)	0.54 (0.16, 2.03)	HAIC			
*0.17 (0.04, 0.69)*	*0.23 (0.07, 0.61)*	0.25 (0.04, 1.31)	0.34 (0.06, 1.46)	0.41 (0.08, 1.63)	0.46 (0.1, 1.79)	0.86 (0.16, 3.67)	HAIC_Sorafenib		
*0.11 (0.01, 0.98)*	0.14 (0.02, 1.02)	0.16 (0.02, 1.77)	0.21 (0.05, 0.93)	0.26 (0.04, 1.84)	0.29 (0.05, 2.16)	0.54 (0.07, 4.66)	0.63 (0.08, 6.42)	TACE_Lenvatinib	

Comparisons should be read from left to right. HRs (95% CrIs) for comparisons are in the cells shared by the column-defining and row-defining interventions. Italicized cells are significant. For OS, PFS, and TTP, an HR < 1 favors the row-defining treatment. For AE, an OR < 1 favors the row-defining treatment. For ORR mRECIST, DCR mRECIST, ORR RECIST, and DCR RECIST, an OR < 1 favors the column-defining treatment

*HAIC* Hepatic arterial infusion chemotherapy, *TACE* Transarterial chemoembolization, *SIRT* Selective internal radiation therapy

### PFS

The included studies reported nine different treatment modalities to compare PFS. The network meta-analysis result is presented in Table 1. HAIC plus sorafenib had significantly better OS than SIRT, sorafenib, and TACE monotherapy (HR 0.38, 95% CrI 0.15–0.91; HR 0.36, 95% CrI 0.18–0.68; HR 0.31, 95% CrI 0.12–0.77, respectively). According to the treatment ranking analysis, TACE plus lenvatinib exhibited the highest likelihood of giving maximal PFS, followed by HAIC plus sorafenib and lenvatinib (Supplementary Material S4 and Supplementary Material S5).

### TTP

The included studies reported seven different treatment modalities to compare TTP. The network meta-analysis result is presented in Table 1. HAIC had significantly better OS than sorafenib and TACE monotherapy (HR 0.24, 95% CrI 0.06–0.92; HR 0.19, 95% CrI 0.04–0.84, respectively). Treatment ranking analysis showed that the likelihood of maximal TTP was the highest with HAIC, followed by TACE plus lenvatinib and SIRT (Supplementary Material S4 and Supplementary Material S5).

### ORR evaluated by mRECIST

The included studies reported eight different treatment modalities to compare ORR evaluated by mRECIST. The network meta-analysis result is presented in Table 1. SIRT showed significant lower ORR than sorafenib, lenvatinib, TACE, HAIC, TACE plus sorafenib, and TACE plus lenvatinib. Compared with sorafenib, HAIC, HAIC plus sorafenib, and TACE plus lenvatinib had significantly higher ORR (sorafenib vs other agents, OR 0.15, 95% CrI 0.05–0.45; OR 0.10, 95% CRI 0.02–0.49; OR 0.07, 95% CrI 0.01–0.70, respectively). Treatment ranking analysis showed that the likelihood of maximal ORR, evaluated by mRECIST, was the highest with TACE plus lenvatinib, followed by HAIC and HAIC plus sorafenib (Supplementary Material S4 and Supplementary Material S5).

### DCR evaluated by mRECIST

The included studies reported seven different treatment modalities to compare DCR evaluated by mRECIST. The network meta-analysis result is presented in Table 1. Sorafenib showed lower DCR than TACE plus lenvatinib (OR 0.09, 95% CrI 0.01–0.89). Treatment ranking analysis indicated that the likelihood of maximal DCR, which was evaluated by mRECIST, was the highest with TACE plus lenvatinib, followed by HAIC and HAIC plus sorafenib (Supplementary Material S4 and Supplementary Material S5).

### ORR evaluated by RECIST

The included studies reported nine different treatment modalities to compare ORR evaluated by RECIST. The network meta-analysis result is presented in Table 1. HAIC, HAIC plus sorafenib, TACE, and TACE plus sorafenib had significantly higher ORR than sorafenib (sorafenib vs. other agents, OR 0.10, 95% CrI 0.01–0.88; OR 0.10, 95% CrI 0.01–0.98; OR 0.03, 95% CrI 0.00–0.39; OR 0.03, 95% CrI 0.00–0.37, respectively). Treatment ranking analysis indicated that the likelihood of maximal ORR, which was evaluated by RECIST, was the highest with HAIC, followed by HAIC plus sorafenib and TACE plus sorafenib (Supplementary Material S4 and Supplementary Material S5).

### DCR evaluated by RECIST

The included studies reported seven different treatment modalities to compare DCR evaluated by RECIST. The network meta-analysis result is presented in Table 1. SIRT showed significantly lower DCR than HAIC, HAIC plus sorafenib, and TACE plus lenvatinib (OR 0.20, 95% CrI 0.05–0.90; OR 0.17, 95% CrI 0.04–0.69; OR 0.11, 95% CrI 0.01–0.98, respectively). Sorafenib showed significantly lower DCR than HAIC and HAIC plus sorafenib (OR 0.26, 95% CrI 0.09–0.83; OR 0.23, 95% CrI 0.07–0.61, respectively). Lenvatinib monotherapy had lower DCR than lenvatinib combined with TACE (OR 0.21, 95% CrI 0.05–0.93). Treatment ranking analysis indicated that the likelihood of maximal DCR, which was evaluated by RECIST, was the highest with TACE plus lenvatinib, followed by HAIC plus sorafenib and HAIC (Supplementary Material S4 and Supplementary Material S5).

### AE

The included studies reported ten different treatment modalities to compare grade 3 or 4 AE. The network meta-analysis result is presented in Table 1. SIRT and TACE showed significantly lower incidence of AEs than TACE plus sorafenib (OR 0.15, 95% CI 0.02–0.90; OR 0.23, 95% CI 0.05–0.76, respectively). Treatment ranking analysis indicated that the likelihood of the minimum AE was the highest with SIRT, followed by HAIC and donafenib (Supplementary Material S4 and Supplementary Material S5).

### Results of quality assessment, global inconsistency, convergence, publication bias, local inconsistency, and heterogeneity analyses

For the qualitative assessment, various domains of bias for each study were investigated using the Cochrane risk of bias tool for RCTs, and the results are summarized in Supplementary Material S6. Deviance information criteria (DIC) were adopted to detect global inconsistency. As shown in Supplementary Material S7, no global inconsistency was found. The preferred model convergence was confirmed using Brooks–Gelman–Rubin in all analyses. The potential scale reduction factor was limited to 1 (Supplementary Material S8), which indicated that the convergence in this analysis was good. Furthermore, the nearly symmetrical funnel plot of representative studies and the p values of the Egger test greater than 0.05 suggested the absence of publication bias (Supplementary Material S9). Indirect and direct evidence of the outcomes of the node-splitting method showed no local consistency (Supplementary Material S10). Some pairwise comparisons were heterogenous when analyzing all outcome measures (Supplementary Material S11). Forest of comparison among interventions and comparison between interventions and sorafenib were shown in Supplementary Material S12 and 13. Results of meta regression for OS were shown in Supplementary Material S14. Meta regression showed that age was a significant factor, however, year of publication, sample size, proportion of male, and proportion of patients with Child A were not significant factors.

## Discussion

Transarterial therapies, TKIs, and a combination of them are presently the main treatment for unresectable HCC. However, a direct comparison of these combinations is challenging because of a number of such drug combinations and fewer cases. Therefore, in this network meta-analysis, which included data from 23 RCTs consisting of 6175 patients with unresectable HCC, we compared the efficacy of these combinations based on their risk categories and by using direct and indirect evidence.

Our network meta-analysis showed that HAIC is superior to sorafenib and TACE, and their combination can improve OS in patients with unresectable HCC. Furthermore, HAIC was also superior to sorafenib and TACE in improving TTP. On the other hand, HAIC plus sorafenib led to better OS than sorafenib in patients with unresectable HCC. Moreover, HAIC plus sorafenib improved PFS compared with SIRT, sorafenib, and TACE. Even though no significant improvement was found in the OS of TACE plus lenvatinib, its rank of OS is ahead, and its ranks of PFS, ORR, and DCR are at the top of the list. These results suggested that HAIC and HAIC plus sorafenib are relatively superior to other monotherapy or combination agents. Moreover, TACE plus lenvatinib is another choice.

HAIC is superior to TACE in directly delivering high doses of chemotherapeutic drugs to the tumor-supplying artery with a higher local concentration and longer persistence, thereby helping in achieving better inhibition of tumor progression [53]. However, tumor necrosis and the upregulation of hypoxia-inducible factors caused by embolism can cause tumor progression [54, 55]. A recent meta-analysis with one RCT and 7 cohort studies showed that the OS and PFS of patients receiving HAIC as the initial therapy were superior to those of patients who received TACE [56], which corroborates the results of the present study.

Although sorafenib significantly improved the survival of patients with advanced HCC compared with placebo, it had lower ORR (2%–3.3%) and DCR (43%–53%) [25, 26]. However, previous studies have shown that HAIC can provide higher ORR and DCR than sorafenib [18, 57], which is consistent with the results of the present study. Thus, patients having unresectable HCC without extrahepatic metastasis can benefit more from HAIC than from sorafenib in terms of OS and PFS [18, 57, 58].

HAIC plus sorafenib can be considered superior to sorafenib in improving OS and PFS owing to the abovementioned reasons. However, we detected significant heterogeneity in OS when comparing HAIC plus sorafenib with sorafenib. The source of heterogeneity can be the different chemotherapy regimens among studies. Several chemotherapeutic drugs of HAIC have been reported, which include fluorouracil, cisplatin, oxaliplatin, leucovorin, doxorubicin, epirubicin, and mitomycin C [12, 17–19, 38–42, 53, 57, 58]. The chemotherapeutic regimens often comprise different combinations of these drugs; however, the optimal combination is still debatable and requires further investigation.

Owing to the increased angiogenesis and upregulation of vascular endothelial growth factor (VEGF) expression after TACE, which resulted in the formation of rich vascular beds in residual tumors, it was considered that additional TKI, such as sorafenib, can improve survival and recurrence. Therefore, Kudo et al. published their research on this topic; however, the result was not satisfactory [35]. Subsequently, other studies have also shown that the combination of TACE and sorafenib did not significantly improve OS compared with the two single treatment regimens [13, 32, 33].

Owing to the remarkable performance of lenvatinib in the REFLECT study, a combination of lenvatinib and TACE is also expected. In a retrospective study, TACE combined with lenvatinib showed its better efficacy in improving OS, PFS, and ORR compared with TACE [59]. Similarly, as shown in LAUNCH trial, TACE combined with lenvatinib better improved OS, PFS, and ORR in patients with advanced HCC compared with lenvatinib monotherapy [37]. The reason, they argued, is that tumor debulking through TACE improved the efficacy of LEN. Additionally, a high ORR of 54.1% made it become downstaging and conversion to surgical therapy. As a result, 26 patients (15.4%) received curative surgical resection after downstaging. In another RCT, TACE combined with lenvatinib provided better OS (despite no significance) and significantly better PFS. Therefore, considering these results and the ranks of OS, PFS, ORR, and DCR, a combination of TACE and lenvatinib is also a reliable option.

## Limitations

This network meta-analysis has many limitations. First, based on our selected topic, we excluded some other TKIs, such as apatinib, regorafenib, and cabozantinib, because they are not first-line TKIs for HCC. However, appropriate studies evaluating those TKIs in combination with TACE were not available except one RCT that assessed the combination of apatinib and TACE [60]; therefore, the efficacy of transarterial therapy plus regorafenib or cabozantinib could not be compared, and we diminutively ignored the comparison between TACE plus apatinib and agents mentioned in this study. Moreover, although we included only RCTs, double-blinding was considered unrealistically in most studies owing to the remarkable difference between transarterial therapies and TKIs. Lastly, unavoidable confounding factors were found in this study, which manifested in the difference in follow-up time, the proportion of BCLC stage B and C, portal vein invasion, extrahepatic metastasis, and HBV/HCV infection. Therefore, heterogeneity was detected in a part of the comparisons. However, subgroup NMA for these confounding factors could not be performed in this study because of limited studies reporting these outcomes.

## Conclusions

Many treatment strategies are available for patients with unresectable HCC. When considering transarterial therapies and TKIs, HAIC and TACE along with lenvatinib are preferred. Further investigation of other potential combinations and the best treatment strategy for patients with unresectable HCC is highly warranted. The results of present study require further validation with data from ongoing head-to-head clinical trials.

## Supplementary Information

**Additional file 1: Supplementary Material S1.** Results of full search strategy of database. **Supplementary Material S2.** Characteristics of included studies in this analysis. **Supplementary Material S3.** Additional information of characteristics of included studies in this analysis. **Supplementary Material S4.** Treatment ranking probability in patients with unresectable HCC. **Supplementary Material S5.** Ranking probabilities of outcomes in the studies.The dark-to-light color indicates the order of the rank. Dark color reflects a better PFS and TTP, lower AE, and higher ORR(mRECIST), DCR(mRECIST), ORR(RECIST), and DCR(RECIST). The size of the bar is proportional to the probability of interventions in each treatment. **Supplementary Material S6.** Quality assessment of the included studies. **Supplementary Material S7.** Results of global inconsistency analysis. **Supplementary Material S8.** Results of convergence. **Supplementary Material S9.** Results of publication bias (the Funnel Plot of Enrolled Trials). **Supplementary Material S10.** Node-splitting method for assessing local inconsistency between direct and indirect evidence. **Supplementary Material S11.** Heterogeneity. **Supplementary Material S12.** Forest plot of the outcomes. **Supplementary Material S13.** Forest plot of the outcomes (compared with sorafenib). **Supplementary Material S14.** Results of meta regression for OS.

## Data Availability

Data can be obtained by contacting corresponding authors.
