# Supplementary material for: Efficacy of transarterial therapy combined with first-line tyrosine kinase inhibitors for unresectable hepatocellular carcinoma: a network meta-analysis
Source: World J Surg Oncol. 2023 Jul 21;21:208. doi: 10.1186/s12957-023-03098-3 (PMC10360255; doi:10.1186/s12957-023-03098-3)
Supplement: Supplementary file 1 — Additional file 1: Supplementary Material S1. Results of full search strategy of database. Supplementary Material S2. Characteristics of included studies in this analysis. Supplementary Material S3. Additional information of characteristics of included studies in this analysis. Supplementary Material S4. Treatment ranking probability in patients with unresectable HCC. Supplementary Material S5. Ranking probabilities of outcomes in the studies.The dark-to-light color indicates the order of the rank. Dark color reflects a better PFS and TTP, lower AE, and higher ORR(mRECIST), DCR(mRECIST), ORR(RECIST), and DCR(RECIST). The size of the bar is proportional to the probability of interventions in each treatment. Supplementary Material S6. Quality assessment of the included studies. Supplementary Material S7. Results of global inconsistency analysis. Supplementary Material S8. Results of convergence. Supplementary Material S9. Results of publication bias (the Funnel Plot of Enrolled Trials). Supplementary Material S10. Node-splitting method for assessing local inconsistency between direct and indirect evidence. Supplementary Material S11. Heterogeneity. Supplementary Material S12. Forest plot of the outcomes. Supplementary Material S13. Forest plot of the outcomes (compared with sorafenib). Supplementary Material S14. Results of meta regression for OS. [file 12957_2023_3098_MOESM1_ESM.docx]

Supplementary Material S1. Results of full search strategy of database.

| Databases (records) | Search strategy |
| --- | --- |
| Pubmed (490) | #1 "liver"[Title/Abstract] OR "hepato*"[Title/Abstract]  #2 "carcinom*"[Title/Abstract] OR "cancer*"[Title/Abstract] OR "neoplasm*"[Title/Abstract] OR "malign*"[Title/Abstract] OR "tumo*"[Title/Abstract]  #3 #1 AND #2  #4 HCC[Title/Abstract]  #5 #3 OR #4  #6 carcinoma, hepatocellular[MeSH Terms]  #7 (liver neoplasms[MeSH Terms])  #8 #5 OR #6 OR #7  #9 sorafenib[Title/Abstract] OR nexavar[Title/Abstract]  #10 lenvatinib[Title/Abstract]  #11 donafenib[Title/Abstract]  #12 chemoembolization, therapeutic[MeSH Terms]  #13 ((TACE[Title/Abstract]) OR (transarterial chemoembolization[Title/Abstract])) OR (transcatheter arterial chemoembolization[Title/Abstract])  #14 #12 OR #13  #15 (hepatic arterial infusion chemotherapy[Title/Abstract]) OR (HAIC[Title/Abstract])  #16 (((radiotherapy[Title/Abstract]) OR (radiation[Title/Abstract])) OR (SIRT[Title/Abstract])) OR (yttrium[Title/Abstract])  #17 #9 OR #10 OR #11 OR #14 OR #15 OR #16  #18 #8 AND #17  #19 (randomized controlled trial[Filter])  #20 AND #18 AND #19 |
| Cochrane Library (2495) | #1 (liver) ti,ab,kw OR (hepato*) ti,ab,kw  #2 (carcinom*) ti,ab,kw OR cancer* ti,ab,kw OR neoplasm* ti,ab,kw OR malign* ti,ab,kw OR tumo* ti,ab,kw  #3 #1 AND #2  #4 HCC ti,ab,kw  #5 #3 OR #4  #6 MeSH descriptor: [carcinoma, hepatocellular] explored all trees  #7 MeSH descriptor: [liver neoplasms] explored all trees  #8 #5 OR #6 OR #7  #9 (sorafenib) ti,ab,kw OR (nexavar) ti,ab,kw  #10 (lenvatinib) ti,ab,kw  #11 (donafenib) ti,ab,kw  #12 chemoembolization, therapeutic[MeSH Terms]  #13 (TACE) ti,ab,kw) OR (transarterial chemoembolization) ti,ab,kw) OR (transcatheter arterial chemoembolization) ti,ab,kw  #14 #12 OR #13  #15 (hepatic arterial infusion chemotherapy) ti,ab,kw OR (HAIC) ti,ab,kw  #16 (radiotherapy) ti,ab,kw OR (radiation) ti,ab,kw OR (SIRT) ti,ab,kw OR (yttrium) ti,ab,kw  #17 #9 OR #10 OR #11 OR #14 OR #15 OR #16  #18 #8 AND #17 |
| Embase (1365) | #1 'liver cell carcinoma'/exp OR 'liver tumor'/exp OR 'hepatocellular carcinoma':ti,ab,kw OR hcc:ti,ab,kw  #2 'sorafenib'/exp OR nexavar:ti,ab,kw OR sorafenib:ti,ab,kw  #3 'chemoembolization'/exp OR 'transarterial chemoembolization':ti,ab,kw OR 'transcatheter arterial chemoembolization':ti,ab,kw OR tace:ti,ab,kw  #4 'radiotherapy'/exp OR 'radiation'/exp OR radiotherapy:ti,ab,kw OR radiation:ti,ab,kw OR sirt:ti,ab,kw OR yttrium:ti,ab,kw  #5 'hepatic artery infusion chemotherapy'/exp OR 'hepatic artery infusion chemotherapy':ti,ab,kw OR haic:ti,ab,kw  #6 #2 OR #3 OR #4 OR #5  #7 #1 AND #6  #8 #7 AND 'randomized controlled trial'/de |
| Web of Science (1602) | #1 TS=(hepatocellular carcinoma) OR TS=(hcc)  #2 TS=(transarterial chemoembolization) OR TS=(transcatheter arterial chemoembolization) OR TS=(TACE)  #3 TS=(sorafenib) OR TS=(nexavar)  #4 TS=(lenvatinib)  #5 TS=(donafenib)  #6 TS=(hepatic artery infusion chemotherapy) OR TS=(HAIC)  #7 TS=(radiotherapy) OR TS=(radiation) OR TS=(SIRT) OR TS=(yttrium)  #8 #2 OR #3 OR #4 OR #5 OR #6 OR #7  #9 #1 AND #8  #10 TS=(randomized controlled trial)  #11 #9 AND #10 |

Supplementary Material S2. Characteristics of included studies in this analysis.

| Study | Phase | Intervention | Sample size | Age | Gender | ECOG | | | Chlid-pugh | | Maximum tumor size | BCLC stage | | | No. of tumors | | AFP | Portal vein invasion | | | | | Extrahepatic metastasis | |
| --- | --- | --- | --- | --- | --- | --- | --- | --- | --- | --- | --- | --- | --- | --- | --- | --- | --- | --- | --- | --- | --- | --- | --- | --- |
|  |  |  |  |  | M/F | 0 | 1 | 2 | A | B | cm | A | B | C | Single | Multiple | ng/ml | Absent | Vp1 | Vp2 | Vp3 | Vp4 | Absent | Present |
| Zheng 2022 | II | Sorafenib +HAIC | 32 | 56±11 | 30/2 | 14 | 16 | 2 | 28 | 4 | 10.6 ± 4.0 | NA | NA | NA | 15 | 17 | 310.5 (117.5-351)^§^ | 0 | 0 | 0 | 14 | 18 | 28 | 4 |
|  |  | Sorafenib | 32 | 55±10 | 31/1 | 14 | 15 | 3 | 27 | 5 | 10.7 ± 3.9 | NA | NA | NA | 12 | 20 | 655.2 (78.2-74277)^§^ | 0 | 0 | 0 | 14 | 18 | 27 | 5 |
| Peng 2022 | III | TACE +Lenvatinib | 170 | 54 (46-64) ^§^ | 139/31 | 89 | 81 | 0 | NA | NA | 8.4(4.5-9.5)^§^ | 170 | 0 | 0 | 30 | 140 | 87 (<400) 83 (≥400) | 48 | 122(vp1+2+3+4) | | | | 76 | 94 |
|  |  | Lenvatinib | 168 | 56 (48-63) ^§^ | 132/36 | 99 | 69 | 0 | NA | NA | 7.4(4.1-9.7)^§^ | 168 | 0 | 0 | 38 | 130 | 81 (<400) 87 (≥400) | 51 | 117(vp1+2+3+4) | | | | 73 | 95 |
| Lyu 2022 | III | HAIC | 130 | 54 (45-61) ^§^ | 115/15 | 15 | 83 | 32 | 88 | 42 | 11.5 ± 4.5 | 0 | 5 | 125 | 43 (1-3) | 87 (>3) | 337.8 (28.5-12,902.75) ^§^ | 41 | 21 (vp1+2) | | 31 | 37 | 86 | 44 |
|  |  | Sorafenib | 132 | 53 (45-62) ^§^ | 123/9 | 14 | 95 | 23 | 93 | 39 | 11.0 ± 3.4 | 0 | 9 | 123 | 55 (1-3) | 77 (>3) | 304.2 (15.3-3,086.5) ^§^ | 49 | 26 (vp1+2) | | 29 | 28 | 86 | 46 |
| Li 2022 | III | HAIC | 159 | 53 (44-63) ^§^ | 135/24 | 95 | 64 | 0 | 159 | 0 | 84 (≤10) 75 (>10) | NA | NA | NA | 60 | 99 | 83 (≤400) 76 (>400) | NA | NA | NA | NA | NA | NA | NA |
|  |  | TACE | 156 | 54 (43-62) ^§^ | 141/15 | 102 | 54 | 0 | 156 | 0 | 84 (≤10) 72 (>10) | NA | NA | NA | 54 | 102 | 75 (≤400) 81 (>400) | NA | NA | NA | NA | NA | NA | NA |
| Elisabeth 2022 | II | SIRT | 38 | 67 (63-72)^§^ | 33/5 | 34 | 4 | 0 | 36 | 2 | 12.8 | 7 | 31 | 0 | 8 | 4 | 33 (<400) 4 (≥400) | 38 | 0 | 0 | 0 | 0 | 38 | 0 |
|  |  | TACE | 34 | 68 (61-71) ^§^ | 30/4 | 29 | 5 | 0 | 29 | 5 | 15 | 4 | 30 | 0 | 30 | 30 | 28 (<400) 5 (≥400) | 34 | 0 | 0 | 0 | 0 | 34 | 0 |
| Chen 2022 | unclear | TACE +Sorafenib | 29 | 66.6±10.4 | 24/5 | 27 | 2 | 0 | 22 | 7 | 4.7±2.6 | 0 | 29 | 0 | 4 | 25 | 20.4 (2.4-6678.3) ^¶^ | NA | NA | NA | NA | NA | NA | NA |
|  |  | TACE | 30 | 62.8±14.3 | 24/6 | 28 | 2 | 0 | 24 | 6 | 5.0±4.2 | 0 | 30 | 0 | 2 | 28 | 174.1 (2.0-107,553.0)^¶^ | NA | NA | NA | NA | NA | NA | NA |
| Qin 2021 | II-III | Dorafenib | 328 | 53 (46-62) ^§^ | 281/47 | 127 | 201 | 0 | 324 | 4 | NA | 0 | 42 | 286 | NA | NA | 208 (8-3,541)^§^ | NA | NA | NA | NA | NA | NA | NA |
|  |  | Sorafenib | 331 | 53 (46-61) ^§^ | 291/40 | 110 | 221 | 0 | 318 | 13 | NA | 0 | 41 | 290 | NA | NA | 210 (9-2,582)^§^ | NA | NA | NA | NA | NA | NA | NA |
| Ding 2021 | unclear | TACE +lenvatinib | 32 | 57 ± 11 | 25/7 | 24 | 8 | 0 | 22 | 10 | 25 (>7.0) 7 (≤7.0) | NA | NA | NA | 16 | 16 | 23731.4 ± 47862.5 | 0 | 21 (vp1+2) | | 11 (vp3+4) | | 19 | 13 |
|  |  | TACE +Sorafenib | 32 | 56 ± 11 | 27/5 | 22 | 10 | 0 | 28 | 4 | 23 (>7.0) 9 (≤7.0) | NA | NA | NA | 13 | 19 | 22862.5 ± 42846.2 | 0 | 25 (vp1+2) | | 7 (vp3+4) | | 23 | 9 |
| Kudo 2020 | unclear | TACE +Sorafenib | 80 | 72(36-85) ^¶^ | 63/17 | 71 | 9 | 0 | 79 | 1 | NA | 27 | 44 | 9 | NA | NA | 64 (<200) 16 (≥200) | 0 | 0 | 0 | 0 | 0 | 0 | 0 |
|  |  | TACE | 76 | 73(53-86) ^¶^ | 55/21 | 67 | 9 | 0 | 71 | 5 | NA | 33 | 34 | 9 | NA | NA | 60 (<200) 16 (≥200) | 0 | 0 | 0 | 0 | 0 | 0 | 0 |
| Ricke 2019 | II | SIRT +Sorafenib | 114 | 43 (<65) 71 (≥ 65) | 100/14 | NA | NA | NA | 107 | 7 | 11.6 ± 5.9 | 4 | 32 | 78 | 16 | 98 | NA | 70 | 44(vp1+2+3+4) | | | | 85 | 29 |
|  |  | Sorafenib | 174 | 75 (<65) 99 (≥ 65) | 151/23 | NA | NA | NA | 160 | 14 | 11.1 ± 5.5 | 3 | 48 | 122 | 34 | 140 | NA | 98 | 76(vp1+2+3+4) | | | | 139 | 35 |
| Park 2019 | III | TACE +Sorafenib | 170 | 60.2 ± 9.6 | 136/34 | 136 | 33 | 1 | 148 | 22 | NA | 3 | 39 | 128 | NA | NA | 7,557.1 ± 22,642.52 | 102 | 20 (vp1+2) | | 48 (vp3+4) | | 108 | 62 |
|  |  | Sorafenib | 169 | 61.3 ± 9.6) | 147/22 | 140 | 28 | 1 | 147 | 22 | NA | 0 | 44 | 125 | NA | NA | 24113.9 ± 168,194.81 | 106 | 15 (vp1+2) | | 48 (vp3+4) | | 110 | 59 |
| Kondo 2019 | II | HAIC +Sorafenib | 35 | 72.0±7.0 | 28/7 | NA | NA | NA | 31 | 4 | NA | 2 | 14 | 19 | NA | NA | 67.3 (2-281,600) ^¶^ | 14 | 21(vp1+2+3+4) | | | | 25 | 10 |
|  |  | Sorafenib | 33 | 70.9±9.1 | 27/6 | NA | NA | NA | 29 | 4 | NA | 2 | 13 | 18 | NA | NA | 216.7 (5-161,160) ^¶^ | 11 | 22(vp1+2+3+4) | | | | 25 | 8 |
| He 2019 | II | HAIC +Sorafenib | 125 | 49 (41-55) ^§^ | 111/14 | 12 | 79 | 34 | 125 | 0 | 10.1 (7.7-13.2) ^§^ | 0 | 0 | 125 | 30 | 95 | 5922 (142.6-56200.5) ^§^ | 0 | 24 | | 54 | 47 | 87 | 38 |
|  |  | Sorafenib | 122 | 49 (40-56) ^§^ | 112/10 | 9 | 83 | 30 | 122 | 0 | 10.1 (8.3-12.1) ^§^ | 0 | 0 | 122 | 33 | 89 | 6666.5 (86.8-49609.8) ^§^ | 0 | 24 | | 53 | 45 | 80 | 42 |
| Kudo 2018 | III | HAIC +Sorafenib | 102 | 66.7±10.2 | 89/13 | 89 | 13 | 0 | 90 | 12 | NA | 0 | 32 | 70 | NA | NA | 440.5^♯^ | NA | NA | | NA | NA | NA | 75 |
|  |  | Sorafenib | 103 | 68.1±9.1 | 88/15 | 91 | 12 | 0 | 93 | 10 | NA | 0 | 27 | 76 | NA | NA | 195^♯^ | NA | NA | | NA | NA | NA | 77 |
| Kudo 2018 | III | Lenvatinib | 478 | 63(20-88) ^¶^ | 405/73 | 304 | 475 | 0 | 475 | 3 | NA | 0 | 104 | 374 | 207 | 270 | 133.1 (8.0-3730.6) ^¶^ | 369 | 109(vp1+2+3+4) | | | | 187 | 291 |
|  |  | Sorafenib | 476 | 62(22-88) ^¶^ | 401/75 | 301 | 471 | 0 | 471 | 5 | NA | 0 | 92 | 384 | 207 | 269 | 71.2 (5.2-1081.8) ^¶^ | 386 | 90(vp1+2+3+4) | | | | 181 | 295 |
| Chow 2018 | III | SIRT | 182 | 59.5 ± 12.9 | 147/35 | 135 | 47 | 0 | 165 | 14 | NA | 0 | 93 | 88 | NA | NA | NA | 126 | 56(vp1+2+3+4) | | | | 182 | 0 |
|  |  | Sorafenib | 178 | 57.7 ± 10.6 | 151/27 | 141 | 37 | 0 | 160 | 16 | NA | 1 | 97 | 80 | NA | NA | NA | 124 | 54(vp1+2+3+4) | | | | 178 | 0 |
| Choi 2018 | unclear | HAIC | 29 | 60.2 ± 7.3 | 27/2 | NA | NA | NA | 25 | 4 | 12(<10) 17(≥10) | NA | NA | NA | 10 | 19 | 130.8 (2.0-225971) ^¶^ | 0 | 0 | 0 | 11 | 18 | NA | NA |
|  |  | Sorafenib | 29 | 60.3 ± 9.5 | 25/4 | NA | NA | NA | 27 | 2 | 14(<10) 15 (≥10) | NA | NA | NA | 13 | 16 | 260.0 (3.6-84604.6) ^¶^ | 0 | 0 | 0 | 10 | 19 | NA | NA |
| Vilgrain 2017 | III | SIRT | 237 | 66(60-72)^§^ | 212/25 | 145 | 92 | 0 | 196 | 39 | NA | 9 | 66 | 162 | 110 | 127 | 87.0 (12.0-2082.0)^§^ | 94 | 29 | | 65 | 49 | NA | NA |
|  |  | Sorafenib | 222 | 65(58-73)^§^ | 202/20 | 139 | 83 | 0 | 187 | 35 | NA | 12 | 61 | 149 | 96 | 126 | 80.0 (8.0-1054.0)^§^ | 104 | 21 | | 59 | 38 | NA | NA |
| Meyer 2017 | III | TACE +Sorafenib | 157 | 65(57-71)^§^ | 139/18 | 98 | 58 | NA | 145 | 5 | 6 (4-8) | NA | NA | NA | 59 | 91 | 23 (5-241) ^§^ (KU/L) | NA | NA | NA | NA | NA | NA | NA |
|  |  | TACE +placecbo | 156 | 68(63-74)^§^ | 138/18 | 97 | 58 | NA | 148 | 3 | 5 (4-8) | NA | NA | NA | 40 | 107 | 25 (5-280) ^§^ (KU/L) | NA | NA | NA | NA | NA | NA | NA |
| Lencioni 2016 | II | TACE +Sorafenib | 154 | 64.5^♯^ | 135/19 | NA | NA | NA | 153 | 1 | NA | NA | 154 | NA | NA | NA | 113(<400) 41 (≥400) | 0 | 0 | 0 | 0 | 0 | 0 | 0 |
|  |  | TACE +placecbo | 153 | 63^♯^ | 126/27 | NA | NA | NA | 152 | 0 | NA | NA | 154 | NA | NA | NA | 112(<400) 41 (≥400) | 0 | 0 | 0 | 0 | 0 | 0 | 0 |
| Ikeda 2016 | II | HAIC +Sorafenib | 65 | 66 (25-79) ^¶^ | 56/9 | 50 | 15 | 0 | 57 | 8 | 5.2 (1.1-17.5) ^¶^ | 0 | 19 | 46 | 8 | 57 | 188 (2-749,412)^¶^ | 25 | 4 | 9 | 14 | 13 | 46 | 19 |
|  |  | Sorafenib | 41 | 64 (42-78) | 32/9 | 33 | 8 | 0 | 39 | 2 | 5.1 (1.0-20.0) ^¶^ | 0 | 16 | 15 | 4 | 38 | 223.5 (1.2-394,944)^¶^ | 28 | 0 | 4 | 7 | 6 | 28 | 13 |
| Piton 2015 | unclear | SIRT | 12 | 71.8 ± 7.2 | 8/4 | 12 | 0 | 0 | 10 | 2 | NA | 0 | 12 | NA | NA | NA | 3308 ± 10204 | 0 | 0 | 0 | 0 | 0 | 0 | 0 |
|  |  | TACE | 12 | 70.5 ± 9.0 | 10/2 | 12 | 0 | 0 | 9 | 3 | NA | 1 | 11 | NA | NA | NA | 164 ± 529 | 0 | 0 | 0 | 0 | 0 | 0 | 0 |
| Kudo 2011 | III | TACE +Sorafenib | 229 | 69^♯^ | 174/55 | 201 | 28 | 0 | 229 | 0 | NA | NA | NA | NA | 167 (≤3) | 62 (>3) | NA | 0 | 0 | 0 | 0 | 0 | 0 | 0 |
|  |  | TACE +placebo | 229 | 70^♯^ | 168/61 | 202 | 27 | 0 | 229 | 0 | NA | NA | NA | NA | 169 (≤3) | 60( >3) | NA | 0 | 0 | 0 | 0 | 0 | 0 | 0 |

Abbreviations: HAIC, hepatic arterial infusion chemotherapy; TACE, transarterial chemoembolization; SIRT, selective internal radiation therapy; ECOG, Eastern Cooperative Oncology Group; BCLC stage, Barcelona clinic liver cancer stage; AFP, alpha-fetoprotein; M, male; F, female; NA, not available.

Vp 1-4 are four types of portal vein tumour thrombus. vp1, it occurs distal to the second grade branch of the portal vein and does not involve the second grade branch; vp 2, it occurs in the secondary branch of the portal vein; vp 3, it occurs in the left or right branches of the portal vein; vp 4, it occurs in the main portal vein.

§, Parenthesis indicates median (interquartile ranges)

¶, Parenthesis indicates median (ranges);

♯, Parenthesis indicates median.

Supplementary Material S3. Additional information of characteristics of included studies in this analysis.

| study | Period | Region | Intervention | Sample size | HBV | HCV | Cirrhosis | | Previous therapy | | | | Survival | | |
| --- | --- | --- | --- | --- | --- | --- | --- | --- | --- | --- | --- | --- | --- | --- | --- |
|  |  |  |  |  |  |  | Yes | No | Absent | Resection | Ablation | TACE | median OS (95%CI) months | median PFS (95%CI) months | median TTP (95%CI) months |
| Zheng 2022 | Jun 2017 to Nov 2019 | China | Sorafenib+HAIC | 32 | 28 | 2 | NA | NA | 22 | 2 | 3 | 5 | 16.3 (0.0 to 35.5) | 9.0 (4.4 to 13.6) | NA |
|  |  |  | Sorafenib | 32 | 29 | 3 | NA | NA | 28 | 0 | 1 | 3 | 6.5 (4.4 to 8.6) | 2.5 (1.3 to 3.7) | NA |
| Peng 2022 | Jun 2019 to Jul 2021 | China | TACE+Lenvatinib | 170 | 148 | 4 | NA | NA | 170 | 0 | 0 | 0 | 17.8 (16.1 to 19.5) | 10.6 (9.5 to 11.7) | NA |
|  |  |  | Lenvatinib | 168 | 144 | 6 | NA | NA | 168 | 0 | 0 | 0 | 11.5 (10.3 to 12.7) | 6.4 (5.8 to 7.0) | NA |
| Lyu 2022 | May 2017 to May 2020 | China | HAIC | 130 | 120 | 2 | NA | NA | 113 | 1 | 3 | 14 | 13.9 (10.6 to 17.2) | 7.8 (6.0 to 9.6) | NA |
|  |  |  | Sorafenib | 132 | 114 | 4 | NA | NA | 113 | 3 | 2 | 16 | 8.2 (7.5 to 9.0) | 4.3 (3.0 to 5.6) | NA |
| Li 2022 | Oct 2016 to Nov 2018 | China | HAIC | 159 | 140 | 2 | NA | NA | NA | NA | NA | NA | 23.1 (18.5 to 27.7) | 9.6 ( 7.4 to 11.9) | NA |
|  |  |  | TACE | 156 | 141 | 3 | NA | NA | NA | NA | NA | NA | 16.1 (14.3 to 17.9) | 5.4 ( 3.8 to 7.0) | NA |
| Elisabeth 2022 | Sep 2011 to Mar 2018 | Belgium | SIRT | 38 | NA | NA | NA | NA | NA | NA | NA | NA | 30.2 | 11.8 | 17.1 |
|  |  |  | TACE | 34 | NA | NA | NA | NA | NA | NA | NA | NA | 15.6 | 9.1 | 9.5 |
| Chen 2022 | Oct 2015 to Apr 2019 | China | TACE+Sorafenib | 29 | 15 | 9 | 24 | 5 | NA | NA | NA | NA | Not reached | 24.8 (1.6 to 48.0) | NA |
|  |  |  | TACE | 30 | 13 | 15 | 26 | 4 | NA | NA | NA | NA | 31.0 (17.8 to 44.2) | 14.5 (9.5 to 20.1) | NA |
| Qin 2021 | Mar 2016 to Apr 2018 | China | Dorafenib | 328 | 293 | 7 | NA | NA | 67 | NA | NA | NA | 12.1 (10.3 to 13.4) | 3.7 (3.0 to 3.7) | NA |
|  |  |  | Sorafenib | 331 | 301 | 5 | NA | NA | 70 | NA | NA | NA | 10.3 (9.2 to 12.0) | 3.6 (2.4 to 3.7) | NA |
| Ding 2021 | Dec 2018 to May 2020 | China | TACE+lenvatinib | 32 | 30 | 1 | NA | NA | NA | NA | NA | NA | 14.5 (8.4 to 20.6) | NA | 4.7 (2.0 to 7.4 ) |
|  |  |  | TACE+Sorafenib | 32 | 29 | 3 | NA | NA | NA | NA | NA | NA | 10.8 (7.7 to 13.9) | NA | 3.1 (2.7 to 3.5 ) |
| Kudo 2020 | Feb 2011 to Mar 2016 | Japan | TACE+Sorafenib | 80 | 20 | 38 | NA | NA | 45 | 0 | 0 | 35 | Not calculated | 25.2 | 26.7 |
|  |  |  | TACE | 76 | 2 | 53 | NA | NA | 48 | 0 | 0 | 28 | Not calculated | 13.5 | 16.4 |
| Ricke 2019 | Jan 2011 to Apr 2016 | Europe and Turkey | SIRT +Sorafenib | 114 | 12 | 28 | 89 | 25 | NA | NA | NA | NA | 14.0 (11.5 to17.0) | NA | NA |
|  |  |  | Sorafenib | 174 | 21 | 37 | 138 | 36 | NA | NA | NA | NA | 11.1 (9.8 to13.8) | NA | NA |
| Park 2019 | Jan 2013 to Dec 2015 | South Korea | TACE+Sorafenib | 170 | 134 | 8 | 116 | 54 | NA | 30 | 34 | 118 | 12.8 (90% CI, 11.5 to 15) | 5.2 (90% CI, 3.7 to 5.6) | 5.3 (90% CI, 3.7 to 5.7) |
|  |  |  | Sorafenib | 169 | 120 | 16 | 116 | 53 | NA | 31 | 40 | 127 | 10.8 (90% CI, 8.7 to 12.7) | 3.6 (90% CI, 2.6 to 3.7) | 3.5 (90% CI, 2.1 to 3.7) |
| Kondo 2019 | Aug 2011 to Nov 2014 | Japan | HAIC+Sorafenib | 35 | 3 | 21 | NA | NA | 9 | 0 | 2 | 24 | 10.0 (7.0 to18.8) | NA | 2.8 (1.7 to 5.5) |
|  |  |  | Sorafenib | 33 | 4 | 20 | NA | NA | 12 | 0 | 1 | 20 | 15.2 (8.2 to 19.7) | NA | 3.9 (2.3 to 6.8) |
| He 2019 | Apr 2016 to Oct 2017 | China | HAIC+Sorafenib | 125 | 100 | 6 | NA | NA | NA | NA | NA | NA | 13.37 (10.27 to 16.46) | 7.03 (6.05 to 8.02) |  |
|  |  |  | Sorafenib | 122 | 99 | 7 | NA | NA | NA | NA | NA | NA | 7.13 (6.28 to 7.98) | 2.6 (2.15 to3.05) |  |
| Kudo 2018 | Nov 2010 to Jun 2014 | Japan | HAIC+Sorafenib | 102 | 22 | 47 | NA | NA | 102 | 0 | 0 | 0 | 11.8(9.1 to 14.5) | 4.8( 3.4 to 6.2) | 5.3 (3.9 to 6.7) |
|  |  |  | Sorafenib | 103 | 22 | 46 | NA | NA | 103 | 0 | 0 | 0 | 11.5 (8.2 to 14.8) | 3.5 (2.6 to 4.4) | 3.5 (2.5 to 4.6) |
| Kudo 2018 | Mar 2013 to Jul 2015 | global | Lenvatinib | 478 | 251 | 91 | 356 | 122 | 327 | NA | NA | NA | 13.6 (12.1 to 14.9) | 7.4 (6.9 to 8.8) | 8.9 (7.4 to 9.2) |
|  |  |  | Sorafenib | 476 | 228 | 126 | 364 | 112 | 344 | NA | NA | NA | 12.3 (10.4 to 13.9) | 3.7 (3.6 to 4.6) | 3.7 (3.6 to 5.4) |
| Chow 2018 | Jul 2010 to May 2016 | Asia-Pacific region | SIRT | 182 | 93 | 26 | NA | NA | NA | NA | NA | NA | 8.8 (7.5 to 10.8) | 5.8 (3.7 to 6.3) | 6.1 (4.2 to 6.6) |
|  |  |  | Sorafenib | 178 | 104 | 19 | NA | NA | NA | NA | NA | NA | 10.0 (8.6 to 13.8) | 5.1 (3.9 to 5.6) | 5.4 (4.1 to 5.7) |
| Choi 2018 | Jan 2013 to Oct 2015 | Korea | HACI | 29 | 18 | 5 | NA | NA | 26 | 3 | 0 | 0 | 14.9 (7.479 to 22.321) | NA | 4.4 (0.550 to 8.250) |
|  |  |  | Sorafenib | 29 | 21 | 0 | NA | NA | 20 | 9 | 0 | 0 | 7.2 (5.434 to 8.966) | NA | 2.7 (2.017 to 3.383) |
| Vilgrain 2017 | Dec 2011 to Mar 2015 | France | SIRT | 237 | 55 | 13 | 211 | 26 | NA | NA | NA | NA | 8.0 (6.7 to 9.9) | 4.1 ( 3.8 to 4.6) | NA |
|  |  |  | Sorafenib | 222 | 49 | 15 | 201 | 21 | NA | NA | NA | NA | 9.9 (8.7 to 11.4) | 3.7 (3.3 to 5.4) | NA |
| Meyer 2017 | Nov 2010 to Dec 2015 | China | TACE+Sorafenib | 157 | 9 | 25 | 129 | 28 | NA | 11 | NA | NA | 631.0 (473.0 to 879.0) days | 238.0 (221.0 to 281.0) days | 326.0 (240.0 to 410.0) |
|  |  |  | TACE+placecbo | 156 | 9 | 21 | 122 | 34 | NA | 20 | NA | NA | 598.0 (500.0 to 697.0) days | 235.0 (209.0 to 322.0)days | 320.0 (234.0 to 400.0) days |
| Lencioni 2016 | NA | global | TACE+Sorafenib | 154 | 55 | 39 | 139 | 15 | NA | NA | NA | NA | Not reached | NA | 169 (166 to 219) days |
|  |  |  | TACE+placecbo | 153 | 50 | 41 | 131 | 22 | NA | NA | NA | NA | Not reached | NA | 166 (113 to 168) days |
| Ikeda 2016 | Jun 2011 to Dec 2013 | Japan | HAIC+Sorafenib | 65 | 22 | 18 | NA | NA | 32 | 17 | 8 | 23 | 10.6 | NA | 3.1 |
|  |  |  | Sorafenib | 41 | 9 | 20 | NA | NA | 20 | 6 | 7 | 14 | 8.7 | NA | 2.8 |
| Piton 2015 | Apr 2010 to Jul 2012 | Germany | SIRT | 12 | 0 | 5 | NA | NA | 5 | 3 | 4 | 0 | 437 ± 72 days | 266 ± 55 days | 353 ± 69 days |
|  |  |  | TACE | 12 | 1 | 4 | NA | NA | 6 | 5 | 1 | 0 | 583 ± 119 days | 237 ± 49 days | 315 ± 69 days |
| Kudo 2011 | Apr 2006 to Jul 2009 | Japan and South Korea | TACE+Sorafenib | 229 | 47 | 139 | 159 | 70 | 119 | 16 | 27 | 0 | 29.7 | NA | 5.4 (3.8 to 7.2) |
|  |  |  | TACE+placebo | 229 | 52 | 148 | 154 | 75 | 110 | 28 | 22 | 0 | Not reached | NA | 3.7 (3.5 to 4.0) |

Abbreviations: HAIC, hepatic arterial infusion chemotherapy; TACE, transarterial chemoembolization; SIRT, selective internal radiation therapy; NA, not available; HBV, hepatitis virus B; HCV, hepatitis virus C; OS, overall survival; PFS, progression-free survival; TTP, time to progression.

Supplementary Material S4. Treatment ranking probability in patients with unresectable HCC

| Intervention | Rank 1 | Rank 2 | Rank 3 | Rank 4 | Rank 5 | Rank 6 | Rank 7 | Rank 8 | Rank 9 | Rank 10 |
| --- | --- | --- | --- | --- | --- | --- | --- | --- | --- | --- |
| OS |  |  |  |  |  |  |  |  |  |  |
| Donafenib | 0.0474 | 0.0719 | 0.1093 | 0.1553 | 0.1308 | 0.0994 | 0.0831 | 0.0775 | 0.0864 | 0.1390 |
| HAIC | 0.3991 | 0.3262 | 0.1552 | 0.0686 | 0.0291 | 0.0123 | 0.0056 | 0.0026 | 0.0009 | 0.0004 |
| HAIC_Sorafenib | 0.0904 | 0.2282 | 0.3250 | 0.1891 | 0.0883 | 0.0429 | 0.0211 | 0.0101 | 0.0038 | 0.0012 |
| Lenvatinib | 0.0035 | 0.0189 | 0.0455 | 0.0963 | 0.1186 | 0.1153 | 0.1114 | 0.1194 | 0.1380 | 0.2332 |
| SIRT | 0.0021 | 0.0120 | 0.0380 | 0.0925 | 0.1585 | 0.1773 | 0.1639 | 0.1494 | 0.1308 | 0.0756 |
| SIRT_Sorafenib | 0.0824 | 0.0911 | 0.1142 | 0.1407 | 0.1198 | 0.0865 | 0.0730 | 0.0703 | 0.0809 | 0.1411 |
| Sorafenib | 0.0000 | 0.0002 | 0.0039 | 0.0258 | 0.0824 | 0.1649 | 0.2247 | 0.2320 | 0.1822 | 0.0839 |
| TACE | 0.0003 | 0.0028 | 0.0122 | 0.0350 | 0.0698 | 0.1076 | 0.1365 | 0.1691 | 0.2164 | 0.2503 |
| TACE_Lenvatinib | 0.3733 | 0.2384 | 0.1579 | 0.0966 | 0.0526 | 0.0308 | 0.0202 | 0.0141 | 0.0119 | 0.0043 |
| TACE_Sorafenib | 0.0014 | 0.0103 | 0.0387 | 0.1001 | 0.1501 | 0.1631 | 0.1606 | 0.1558 | 0.1488 | 0.0711 |
| PFS |  |  |  |  |  |  |  |  |  |  |
| Donafenib | 0.0187 | 0.0447 | 0.0966 | 0.1262 | 0.1261 | 0.1312 | 0.1143 | 0.1302 | 0.2119 |  |
| HAIC | 0.0090 | 0.0606 | 0.2220 | 0.2630 | 0.1920 | 0.1154 | 0.0708 | 0.0470 | 0.0201 |  |
| HAIC_Sorafenib | 0.3262 | 0.4964 | 0.1245 | 0.0338 | 0.0107 | 0.0049 | 0.0022 | 0.0010 | 0.0004 |  |
| Lenvatinib | 0.0072 | 0.1103 | 0.2872 | 0.1821 | 0.1341 | 0.0907 | 0.0652 | 0.0619 | 0.0613 |  |
| SIRT | 0.0030 | 0.0137 | 0.0441 | 0.0905 | 0.1374 | 0.1719 | 0.1850 | 0.1945 | 0.1599 |  |
| Sorafenib | 0.0001 | 0.0011 | 0.0062 | 0.0308 | 0.0962 | 0.2087 | 0.3056 | 0.2561 | 0.0955 |  |
| TACE | 0.0007 | 0.0029 | 0.0099 | 0.0271 | 0.0628 | 0.1168 | 0.1429 | 0.2119 | 0.4250 |  |
| TACE_Lenvatinib | 0.6222 | 0.2173 | 0.0625 | 0.0315 | 0.0192 | 0.0131 | 0.0116 | 0.0126 | 0.0101 |  |
| TACE_Sorafenib | 0.0130 | 0.0530 | 0.1471 | 0.2149 | 0.2215 | 0.1472 | 0.1024 | 0.0849 | 0.0159 |  |
| TTP |  |  |  |  |  |  |  |  |  |  |
| HAIC | 0.7567 | 0.1638 | 0.0336 | 0.0182 | 0.0115 | 0.0081 | 0.0081 |  |  |  |
| HAIC_Sorafenib | 0.0069 | 0.0739 | 0.2010 | 0.2422 | 0.2468 | 0.1467 | 0.0824 |  |  |  |
| SIRT | 0.0212 | 0.1544 | 0.3419 | 0.2415 | 0.1445 | 0.0741 | 0.0224 |  |  |  |
| Sorafenib | 0.0003 | 0.0072 | 0.0387 | 0.1137 | 0.2552 | 0.4207 | 0.1641 |  |  |  |
| TACE | 0.0001 | 0.0013 | 0.0064 | 0.0336 | 0.1015 | 0.1554 | 0.7016 |  |  |  |
| TACE_Lenvatinb | 0.2116 | 0.5559 | 0.1034 | 0.0493 | 0.0354 | 0.0286 | 0.0158 |  |  |  |
| TACE_Sorafenib | 0.0031 | 0.0435 | 0.2749 | 0.3014 | 0.2051 | 0.1663 | 0.0056 |  |  |  |
| AE |  |  |  |  |  |  |  |  |  |  |
| Donafenib | 0.2192 | 0.1348 | 0.1277 | 0.1224 | 0.1135 | 0.0928 | 0.0799 | 0.0541 | 0.0348 | 0.0209 |
| HAIC | 0.2161 | 0.2109 | 0.1974 | 0.1581 | 0.1006 | 0.0583 | 0.0351 | 0.0161 | 0.0058 | 0.0017 |
| HAIC_Sorafenib | 0.0062 | 0.0124 | 0.0191 | 0.0276 | 0.0424 | 0.0755 | 0.1608 | 0.2673 | 0.2186 | 0.1701 |
| Lenvatinib | 0.0554 | 0.0564 | 0.0628 | 0.0764 | 0.0941 | 0.1211 | 0.1941 | 0.1506 | 0.1103 | 0.0790 |
| SIRT | 0.2319 | 0.2910 | 0.2167 | 0.1310 | 0.0671 | 0.0349 | 0.0173 | 0.0070 | 0.0026 | 0.0005 |
| SIRT_Sorafenib | 0.1791 | 0.1290 | 0.1170 | 0.1138 | 0.1043 | 0.0969 | 0.0981 | 0.0752 | 0.0493 | 0.0373 |
| Sorafenib | 0.0024 | 0.0150 | 0.0556 | 0.1434 | 0.2683 | 0.3166 | 0.1533 | 0.0371 | 0.0078 | 0.0007 |
| TACE | 0.0775 | 0.1366 | 0.1846 | 0.1975 | 0.1607 | 0.1198 | 0.0829 | 0.0351 | 0.0049 | 0.0005 |
| TACE_Lenvatinib | 0.0119 | 0.0117 | 0.0140 | 0.0174 | 0.0236 | 0.0332 | 0.0550 | 0.0935 | 0.1628 | 0.5769 |
| TACE_Sorafenib | 0.0004 | 0.0022 | 0.0052 | 0.0124 | 0.0256 | 0.0509 | 0.1236 | 0.2641 | 0.4033 | 0.1124 |
| ORR mRECIST |  |  |  |  |  |  |  |  |  |  |
| HAIC | 0.2483 | 0.3276 | 0.2099 | 0.1272 | 0.0589 | 0.0261 | 0.0020 | 0.0000 |  |  |
| HAIC_Sorafenib | 0.1129 | 0.1958 | 0.2250 | 0.1504 | 0.1839 | 0.1280 | 0.0039 | 0.0001 |  |  |
| Lenvatinib | 0.0151 | 0.0683 | 0.1046 | 0.1803 | 0.1898 | 0.3849 | 0.0557 | 0.0013 |  |  |
| SIRT | 0.0021 | 0.0013 | 0.0013 | 0.0017 | 0.0027 | 0.0044 | 0.0106 | 0.9761 |  |  |
| Sorafenib | 0.0000 | 0.0008 | 0.0056 | 0.0143 | 0.0397 | 0.0843 | 0.8340 | 0.0212 |  |  |
| TACE | 0.0267 | 0.0730 | 0.1478 | 0.2256 | 0.2594 | 0.2197 | 0.0475 | 0.0004 |  |  |
| TACE_Lenvatinib | 0.5557 | 0.2052 | 0.1241 | 0.0623 | 0.0354 | 0.0126 | 0.0048 | 0.0002 |  |  |
| TACE_Sorafenib | 0.0392 | 0.1280 | 0.1817 | 0.2383 | 0.2304 | 0.1402 | 0.0415 | 0.0007 |  |  |
| DCR mRECIST |  |  |  |  |  |  |  |  |  |  |
| HAIC | 0.1381 | 0.4468 | 0.2105 | 0.1235 | 0.0565 | 0.0194 | 0.0053 |  |  |  |
| HAIC_Sorafenib | 0.0328 | 0.1055 | 0.2063 | 0.1976 | 0.1867 | 0.2146 | 0.0563 |  |  |  |
| Lenvatinib | 0.0130 | 0.1445 | 0.1924 | 0.1848 | 0.1962 | 0.1482 | 0.1210 |  |  |  |
| Sorafenib | 0.0006 | 0.0045 | 0.0209 | 0.0764 | 0.1751 | 0.2354 | 0.4872 |  |  |  |
| TACE | 0.0155 | 0.0631 | 0.1565 | 0.2004 | 0.1959 | 0.2023 | 0.1664 |  |  |  |
| TACE_Lenvatinib | 0.7833 | 0.1233 | 0.0474 | 0.0251 | 0.0131 | 0.0052 | 0.0025 |  |  |  |
| TACE_Sorafenib | 0.0168 | 0.1124 | 0.1660 | 0.1922 | 0.1765 | 0.1749 | 0.1613 |  |  |  |
| ORR RECIST |  |  |  |  |  |  |  |  |  |  |
| Donafenib | 0.0172 | 0.0300 | 0.0450 | 0.0544 | 0.0882 | 0.1416 | 0.1722 | 0.1864 | 0.2651 |  |
| HAIC | 0.4447 | 0.3023 | 0.1161 | 0.0627 | 0.0368 | 0.0201 | 0.0109 | 0.0044 | 0.0020 |  |
| HAIC_Sorafenib | 0.3613 | 0.2863 | 0.1321 | 0.0923 | 0.0625 | 0.0374 | 0.0181 | 0.0075 | 0.0026 |  |
| Lenvatinib | 0.0024 | 0.0105 | 0.0202 | 0.0350 | 0.0666 | 0.1628 | 0.2563 | 0.2419 | 0.2043 |  |
| SIRT | 0.0218 | 0.0538 | 0.0938 | 0.1149 | 0.1945 | 0.2197 | 0.1725 | 0.0894 | 0.0397 |  |
| Sorafenib | 0.0000 | 0.0001 | 0.0007 | 0.0030 | 0.0140 | 0.0651 | 0.1758 | 0.3549 | 0.3865 |  |
| TACE | 0.0256 | 0.1172 | 0.2531 | 0.2598 | 0.1854 | 0.0972 | 0.0410 | 0.0156 | 0.0052 |  |
| TACE_Lenvatinib | 0.0965 | 0.0939 | 0.1094 | 0.1028 | 0.1532 | 0.1553 | 0.1118 | 0.0864 | 0.0907 |  |
| TACE_Sorafenib | 0.0306 | 0.1059 | 0.2297 | 0.2750 | 0.1987 | 0.1009 | 0.0415 | 0.0137 | 0.0041 |  |
| DCR RECIST |  |  |  |  |  |  |  |  |  |  |
| Donafenib | 0.0089 | 0.0198 | 0.0368 | 0.0691 | 0.0791 | 0.1308 | 0.2469 | 0.1960 | 0.2127 |  |
| HAIC | 0.1234 | 0.3377 | 0.3670 | 0.0879 | 0.0427 | 0.0227 | 0.0108 | 0.0051 | 0.0027 |  |
| HAIC_Sorafenib | 0.2315 | 0.3876 | 0.2528 | 0.0620 | 0.0352 | 0.0199 | 0.0069 | 0.0030 | 0.0012 |  |
| Lenvatinib | 0.0012 | 0.0169 | 0.0466 | 0.1249 | 0.1746 | 0.3132 | 0.1473 | 0.0967 | 0.0786 |  |
| SIRT | 0.0009 | 0.0039 | 0.0091 | 0.0163 | 0.0284 | 0.0512 | 0.1084 | 0.1997 | 0.5821 |  |
| Sorafenib | 0.0000 | 0.0003 | 0.0023 | 0.0090 | 0.0285 | 0.0914 | 0.3419 | 0.4353 | 0.0913 |  |
| TACE | 0.0036 | 0.0185 | 0.0669 | 0.2436 | 0.3133 | 0.2131 | 0.0837 | 0.0381 | 0.0191 |  |
| TACE_Lenvatinib | 0.6228 | 0.1773 | 0.1001 | 0.0374 | 0.0249 | 0.0137 | 0.0093 | 0.0087 | 0.0058 |  |
| TACE_Sorafenib | 0.0077 | 0.0381 | 0.1185 | 0.3497 | 0.2734 | 0.1440 | 0.0448 | 0.0173 | 0.0066 |  |

Abbreviations: HAIC, hepatic arterial infusion chemotherapy; TACE, transarterial chemoembolization; SIRT, selective internal radiation therapy.

Supplementary Material S5. Ranking probabilities of outcomes in the studies.The dark-to-light color indicates the order of the rank. Dark color reflects a better PFS and TTP, lower AE, and higher ORR(mRECIST), DCR(mRECIST), ORR(RECIST), and DCR(RECIST). The size of the bar is proportional to the probability of interventions in each treatment.

PFS
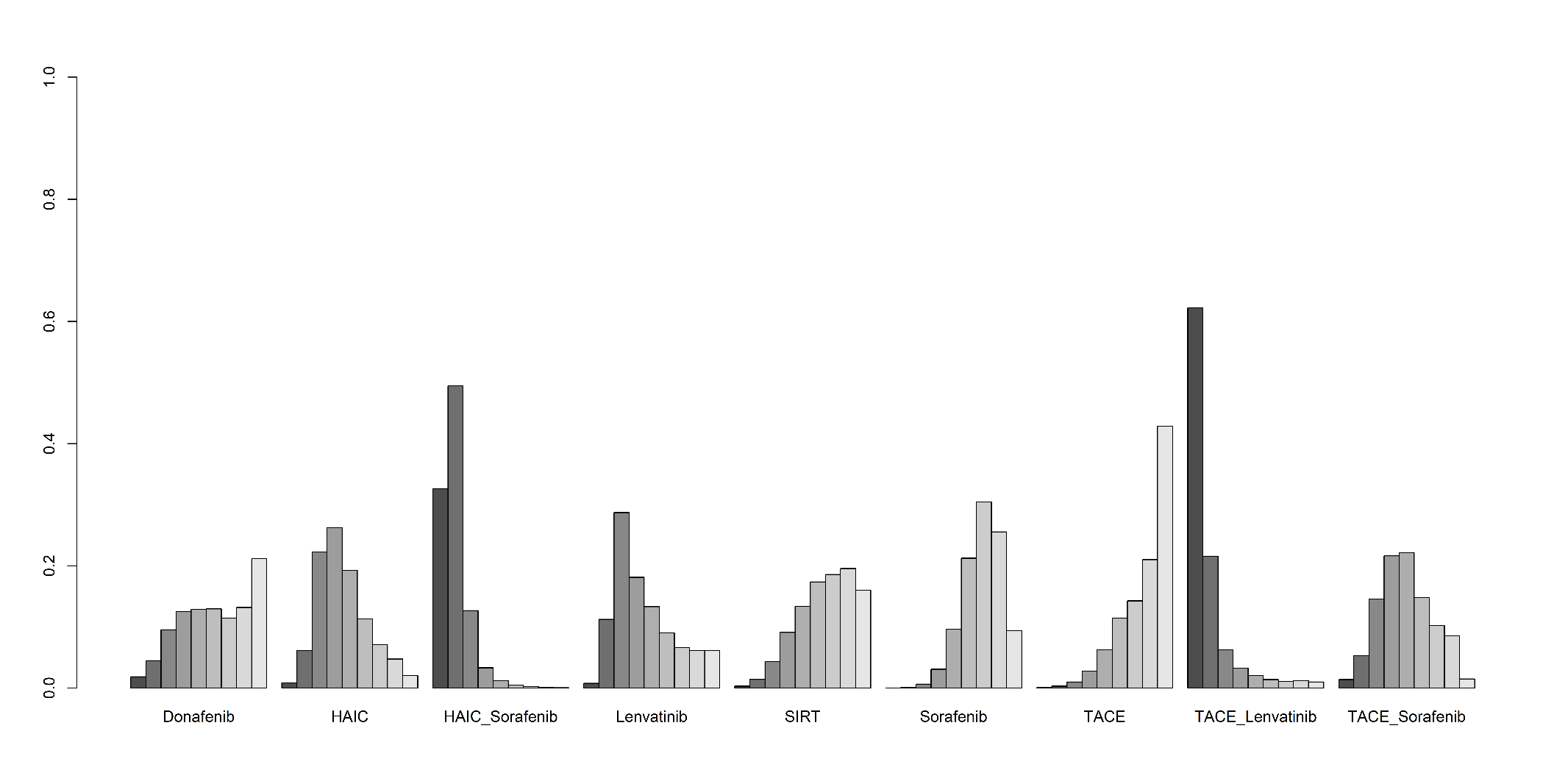


TTP


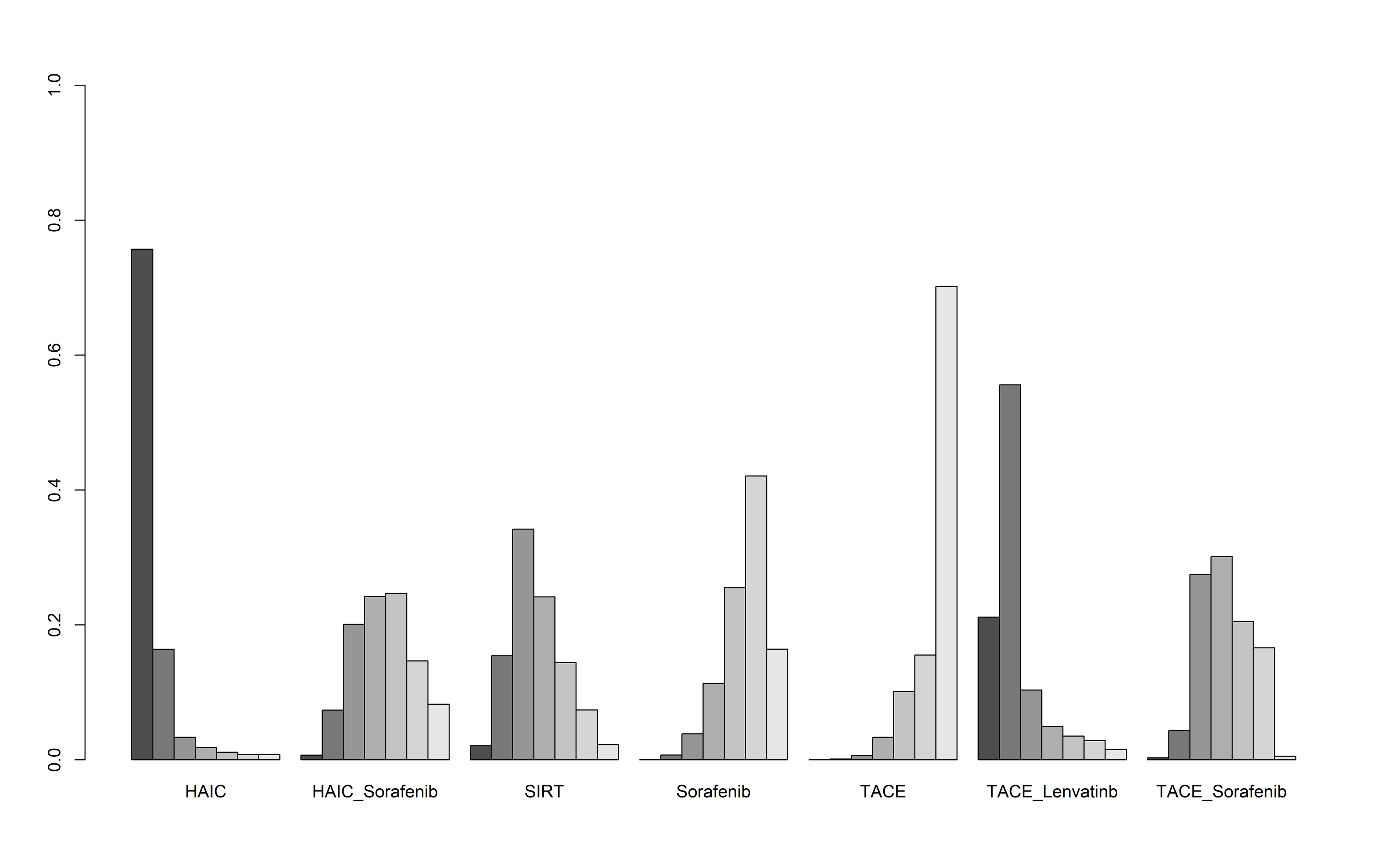


AE


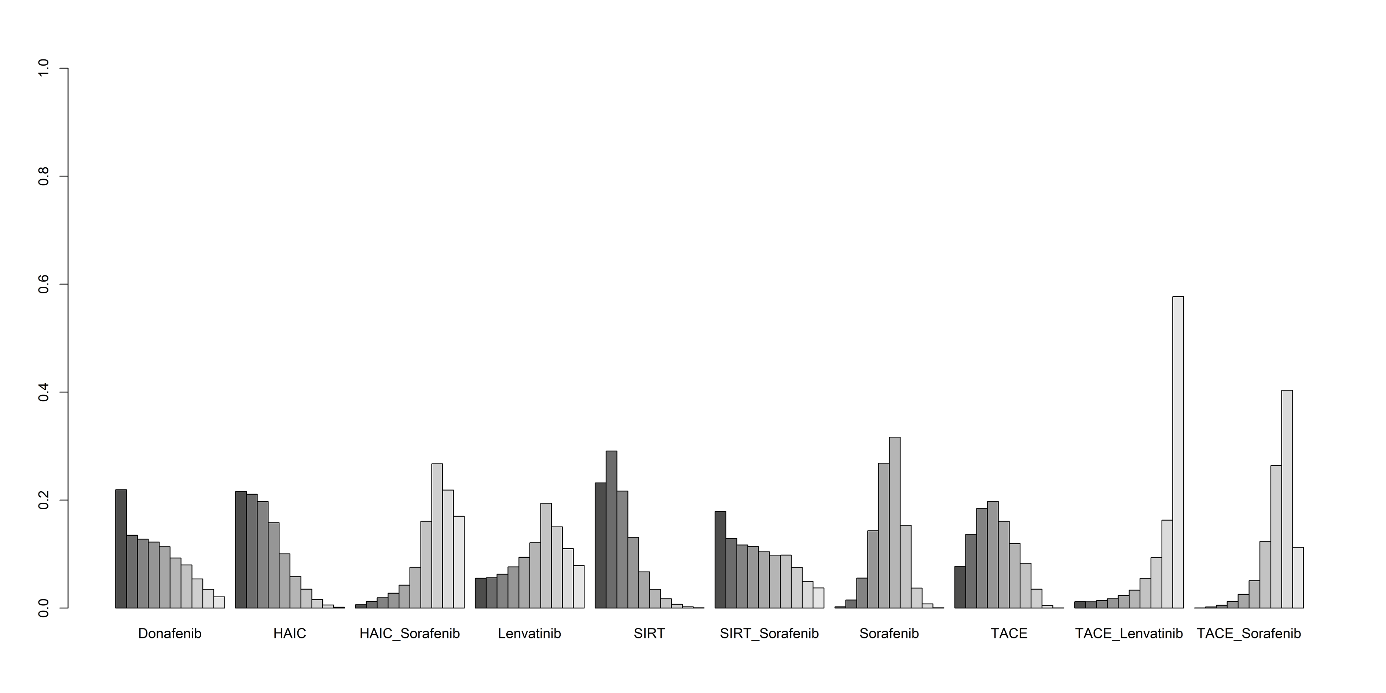


ORR(mRECIST)


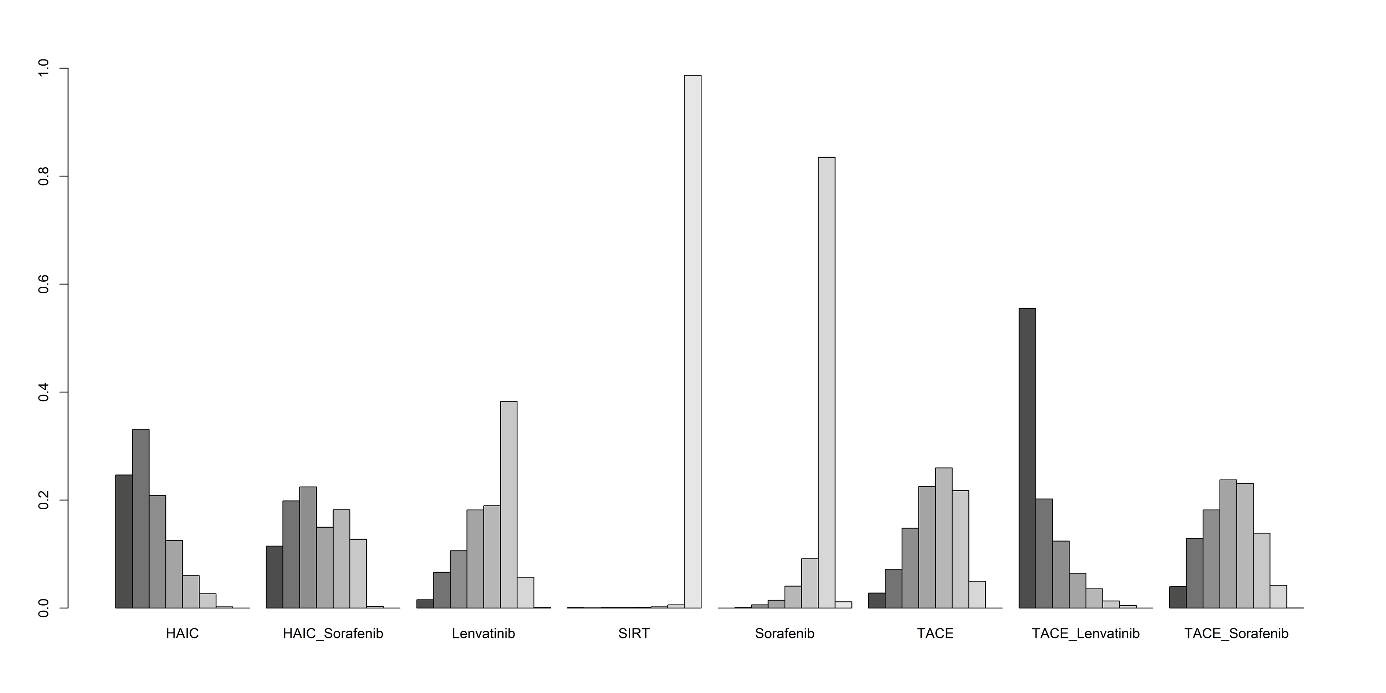


DCR(mRECIST)


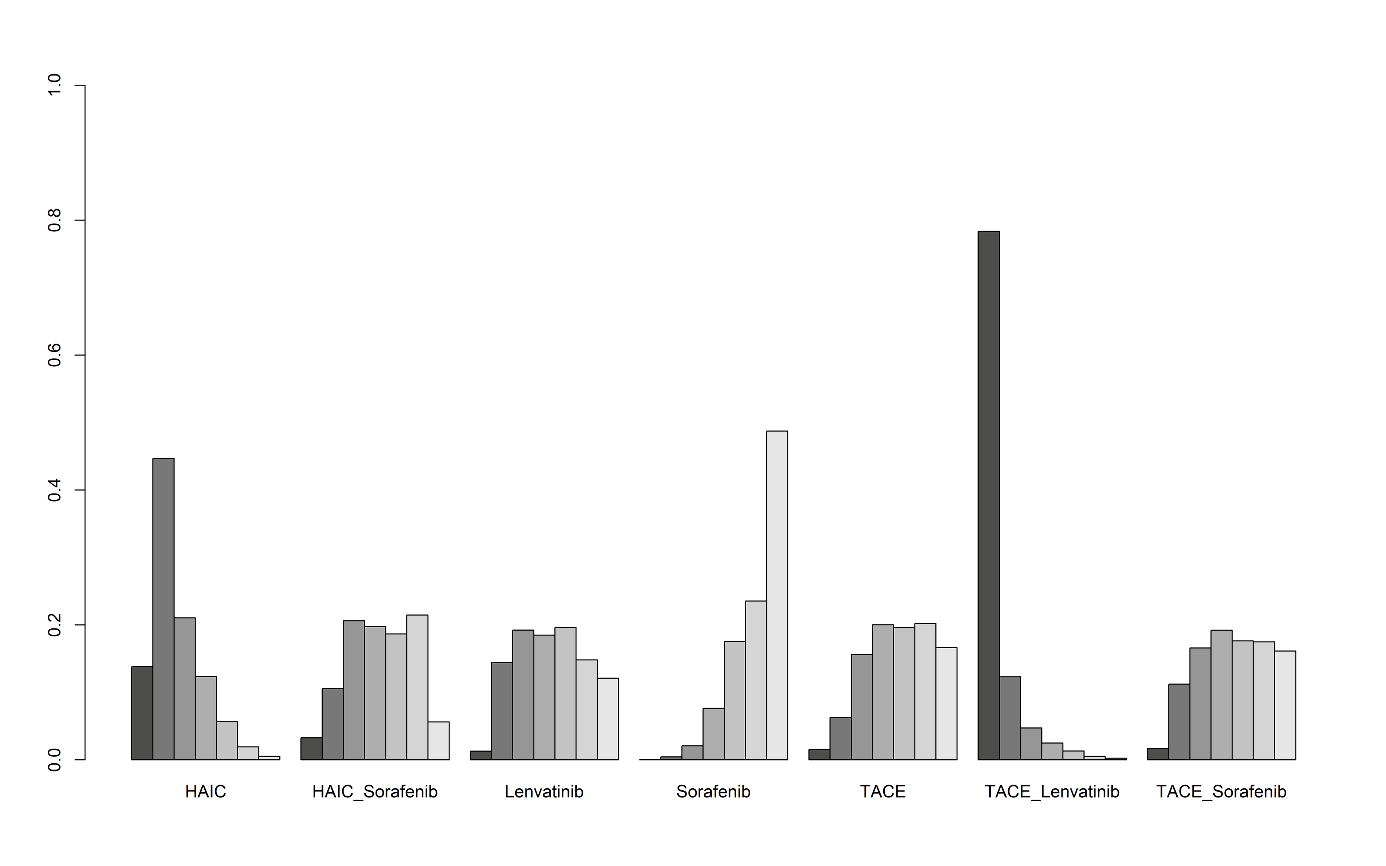


ORR(RECIST)


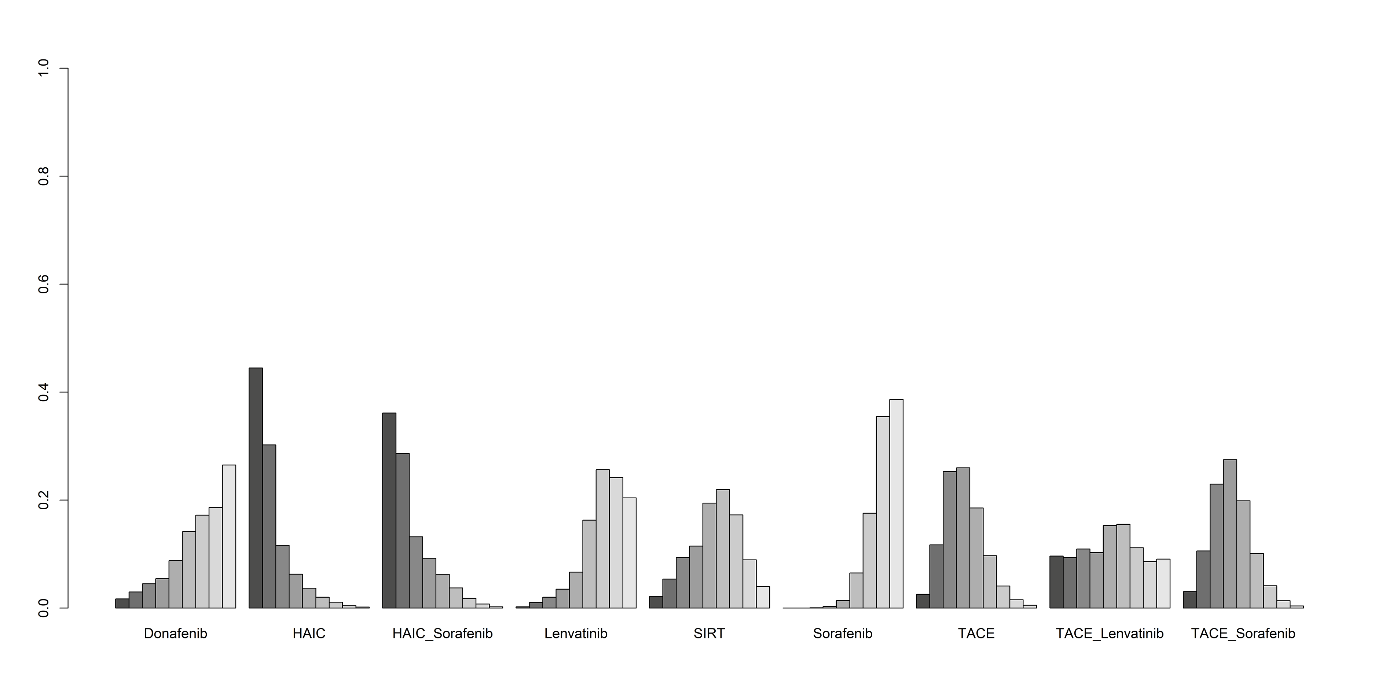


DCR(RECIST)


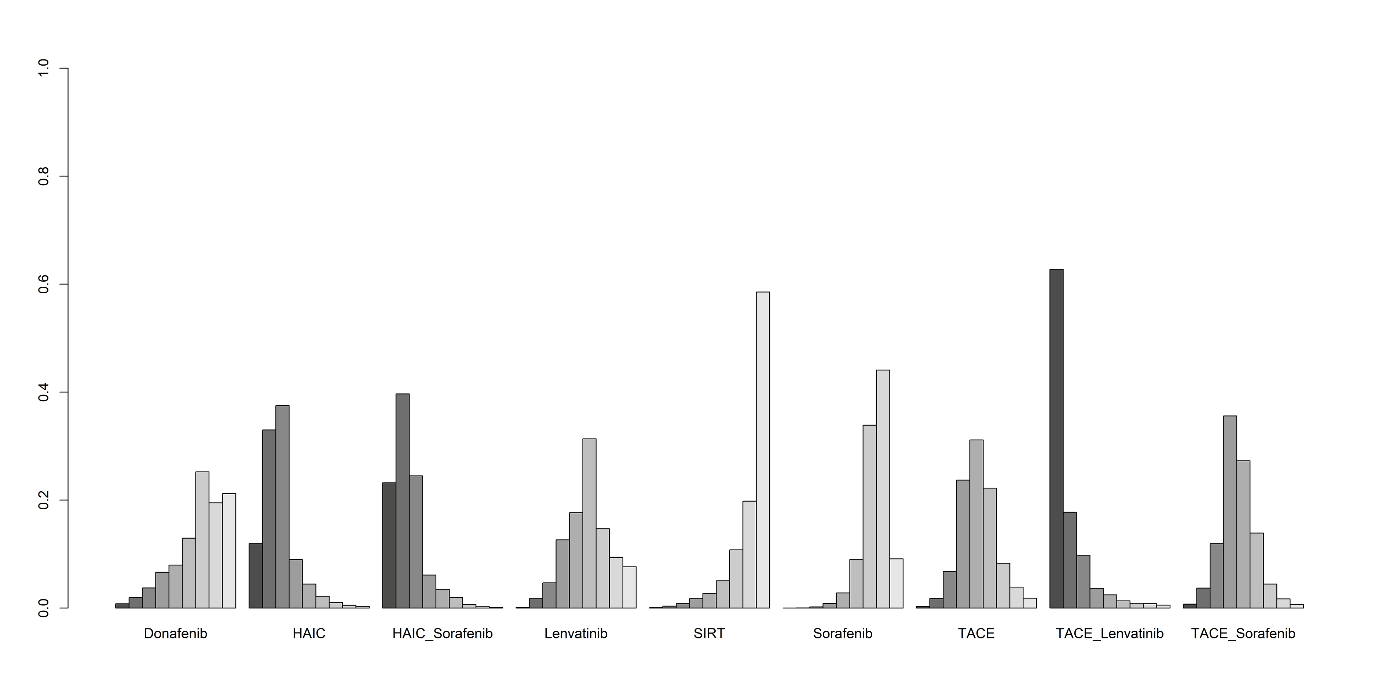


Supplementary Material S6. Quality assessment of the included studies


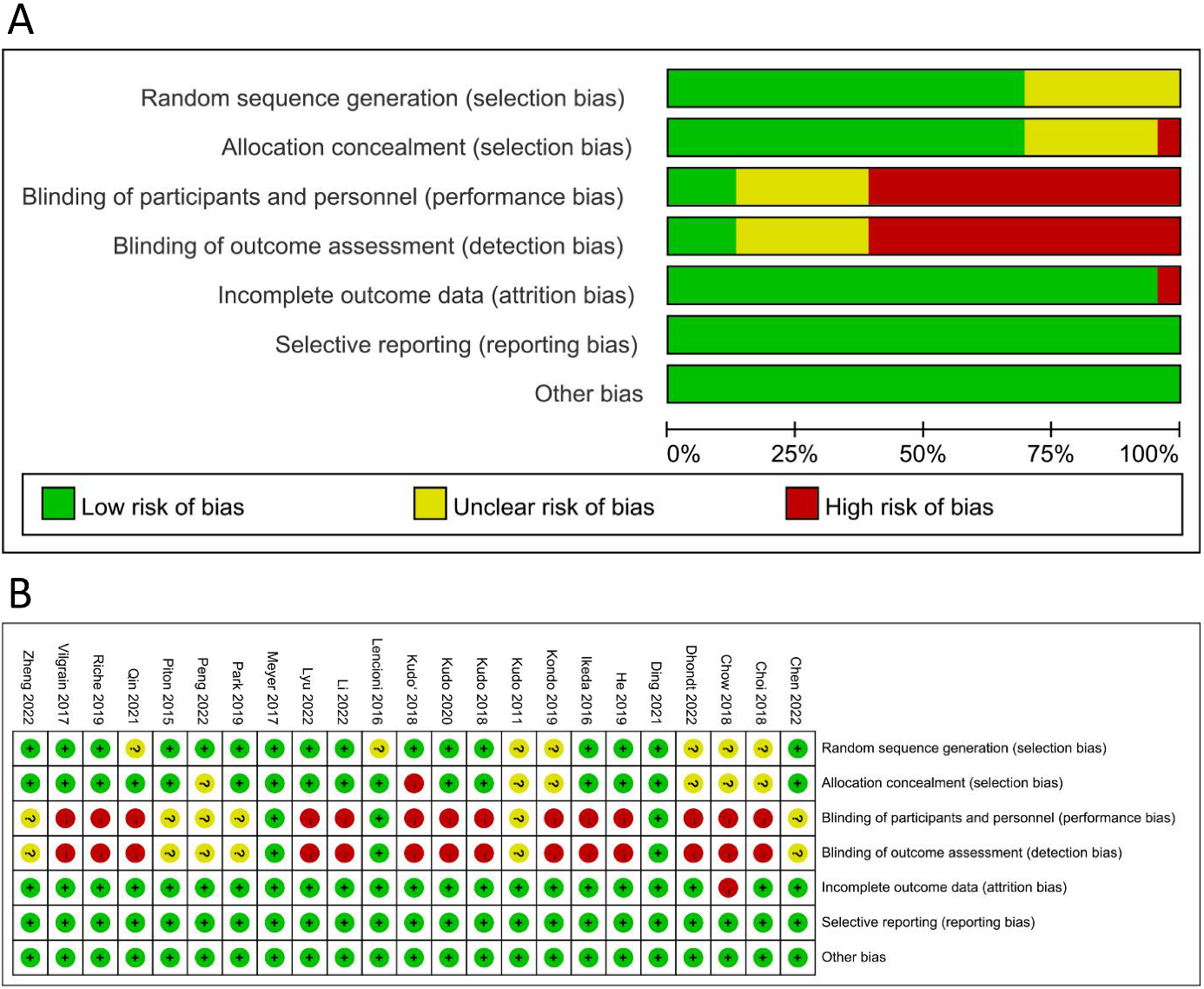


Supplementary Material S7. Results of global inconsistency analysis

Random-effect model was used to reported outcomes. Deviance Information Criteria [DIC] was adopted to detect global inconsistency. The difference values of DICs between consistency and inconsistency model are all less than 1, indicating that data is consistent. Further, values of I^2^ <50% was considered no considerable heterogeneity.

| Intervention | DIC | | I^2^ | |
| --- | --- | --- | --- | --- |
|  | consistency model (random) | inconsistency model (random) | consistency model (random) | inconsistency model (random) |
| OS | 39.88158 | 40.29928 | 7% | 7% |
| PFS | 29.1523 | 29.77858 | 8% | 8% |
| TTP | 23.03424 | 22.63483 | 13% | 13% |
| ORR RECIST | 47.49009 | 48.02111 | 4% | 5% |
| ORR mRECIST | 53.14147 | 53.24452 | 3% | 2% |
| DCR RECIST | 47.50724 | 48.12866 | 6% | 6% |
| DCR mRECIST | 47.82172 | 47.98319 | 6% | 6% |
| AE | 63.93552 | 63.55437 | 7% | 5% |
|  |  |  |  |  |

Abbreviations:OS, overall survival; PFS, progression-free survival; TTP, time to progression; ORR, object response rate; DCR, disease control rate; RECIST, Response Evaluation Criteria In Solid Tumors; AE, adverse effect.

Supplementary Material S8. Results of convergence

Supplementary Material S8.1 Brooks-Gelman-Rubin plot

OS
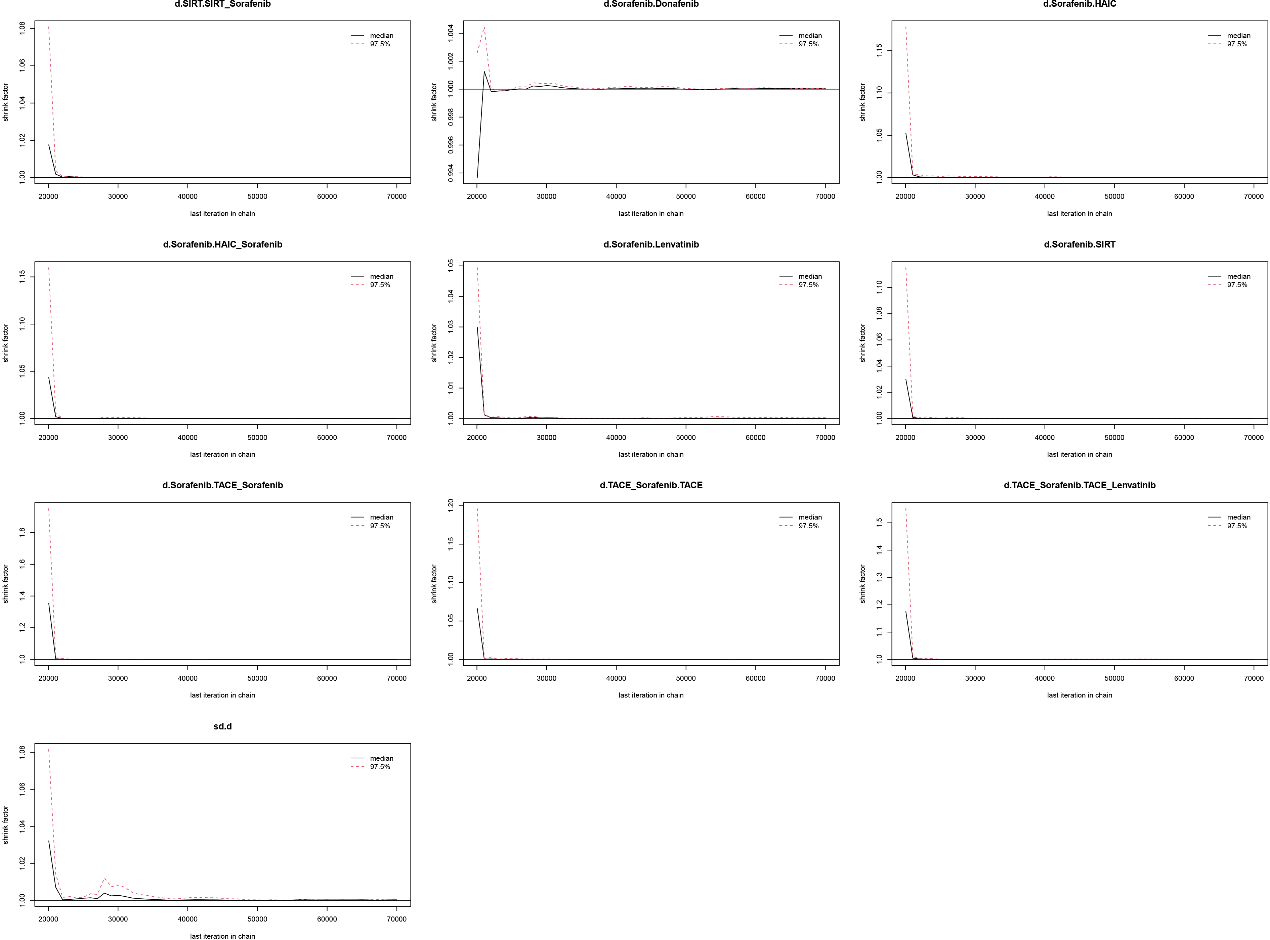


PFS
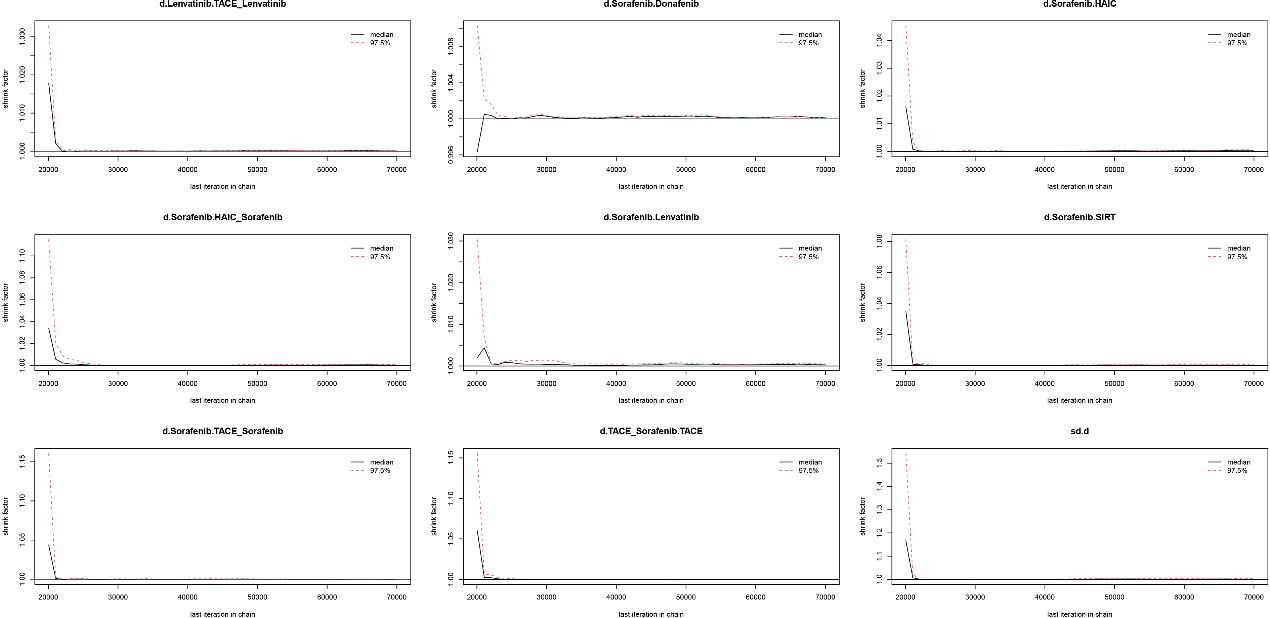


TTP
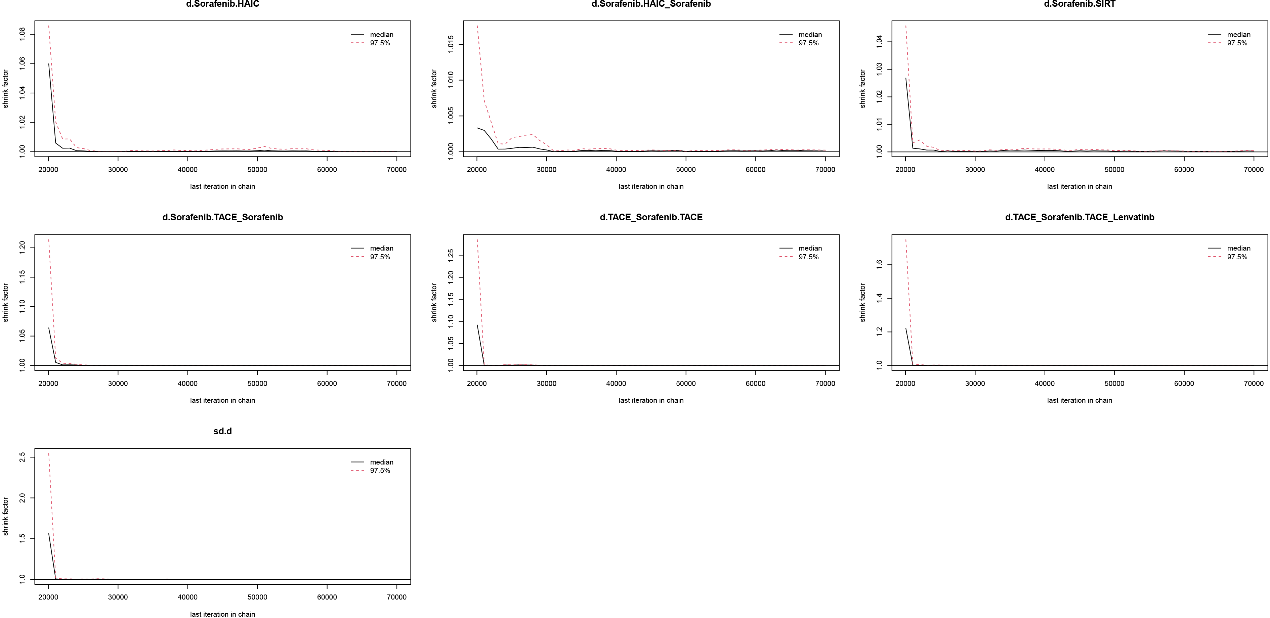


AE
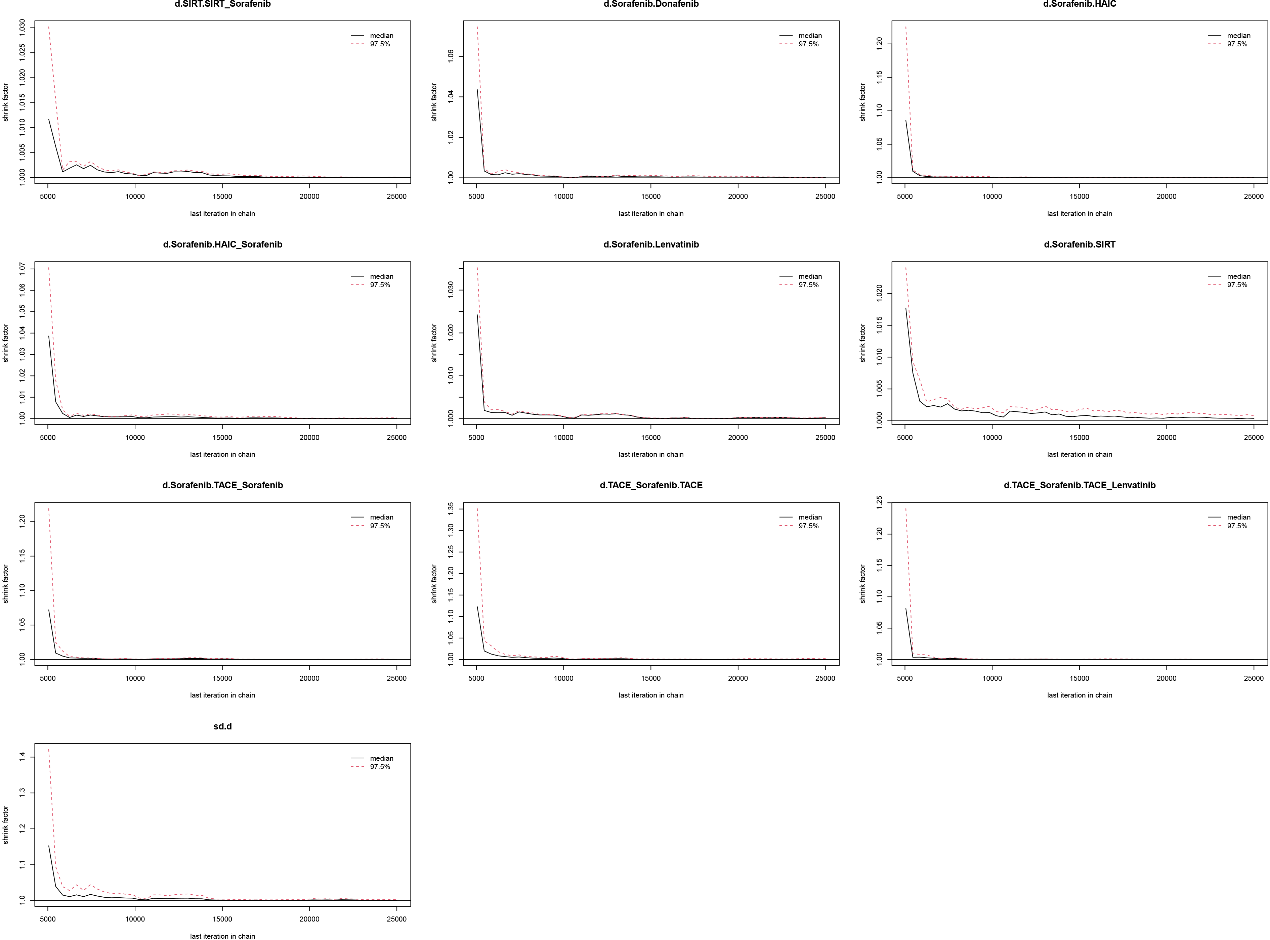


ORR(mRECIST)
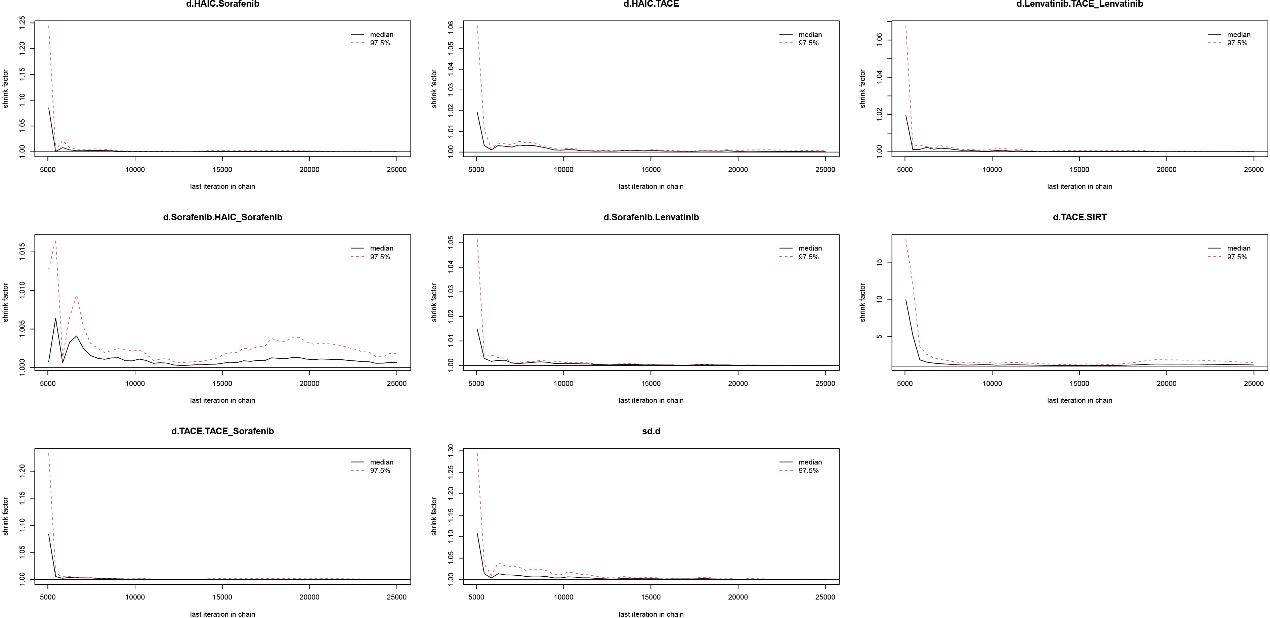


DCR(mRECIST)
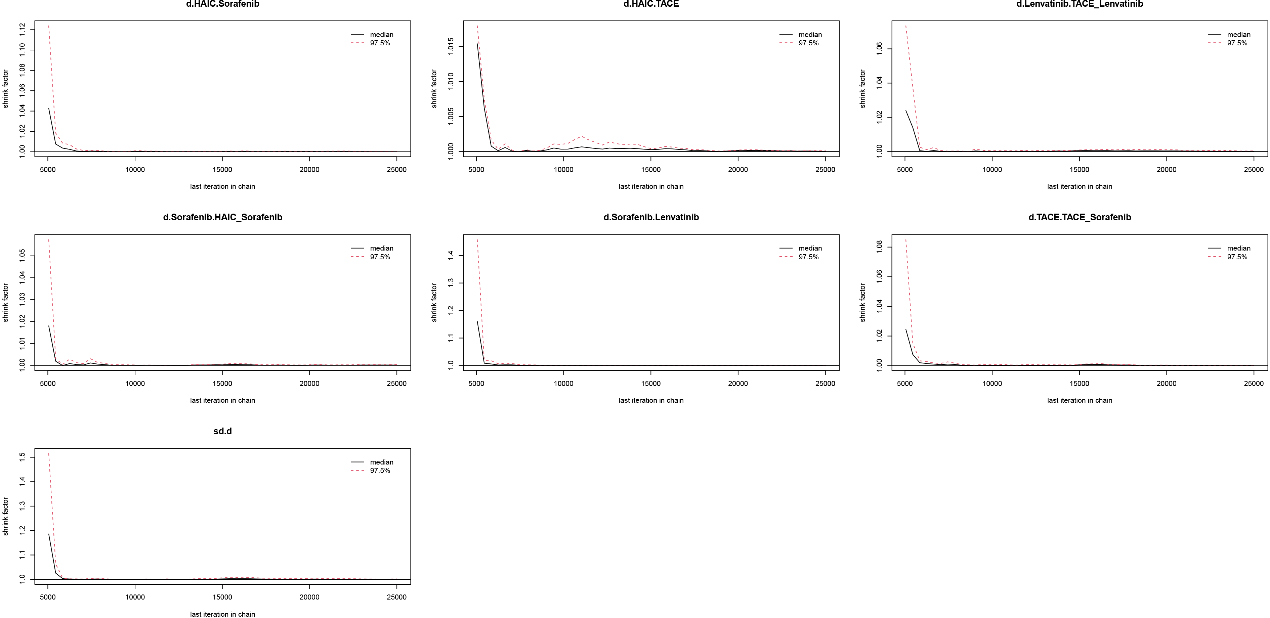


ORR(RECIST)
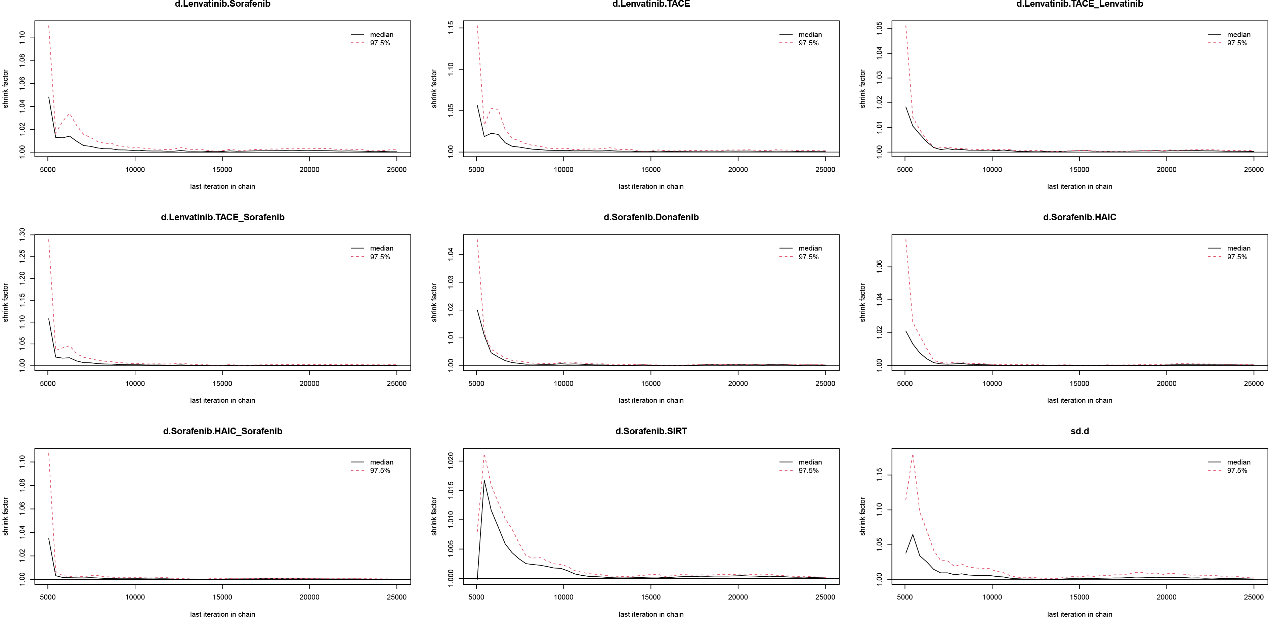
DCR(RECIST)
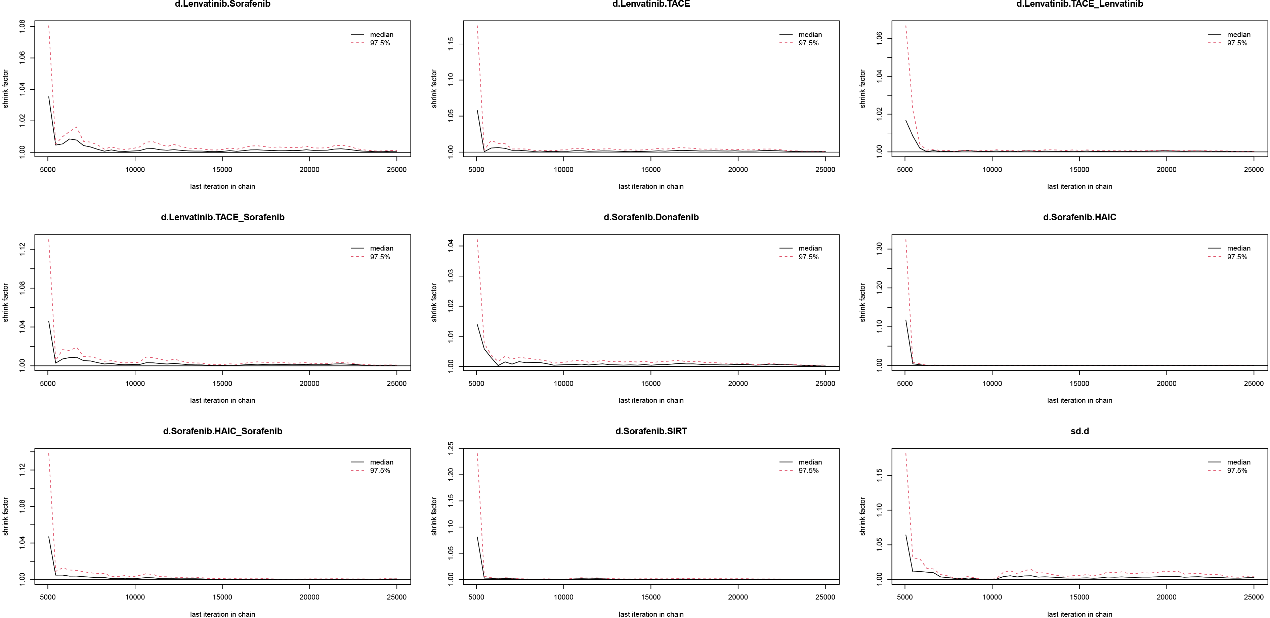


Supplementary Material S8.2 Trace plot and density plot. As can be seen from the trajectory diagrams, when the number of iterations reaches more than 5000, MCMC chain fluctuation is stable and has good overlap. It can be seen from the density diagram that when the number of iterations reaches 20,000, Bandwith tends to 0 and reaches stability, which comprehensively indicates that the model converges well.

OS
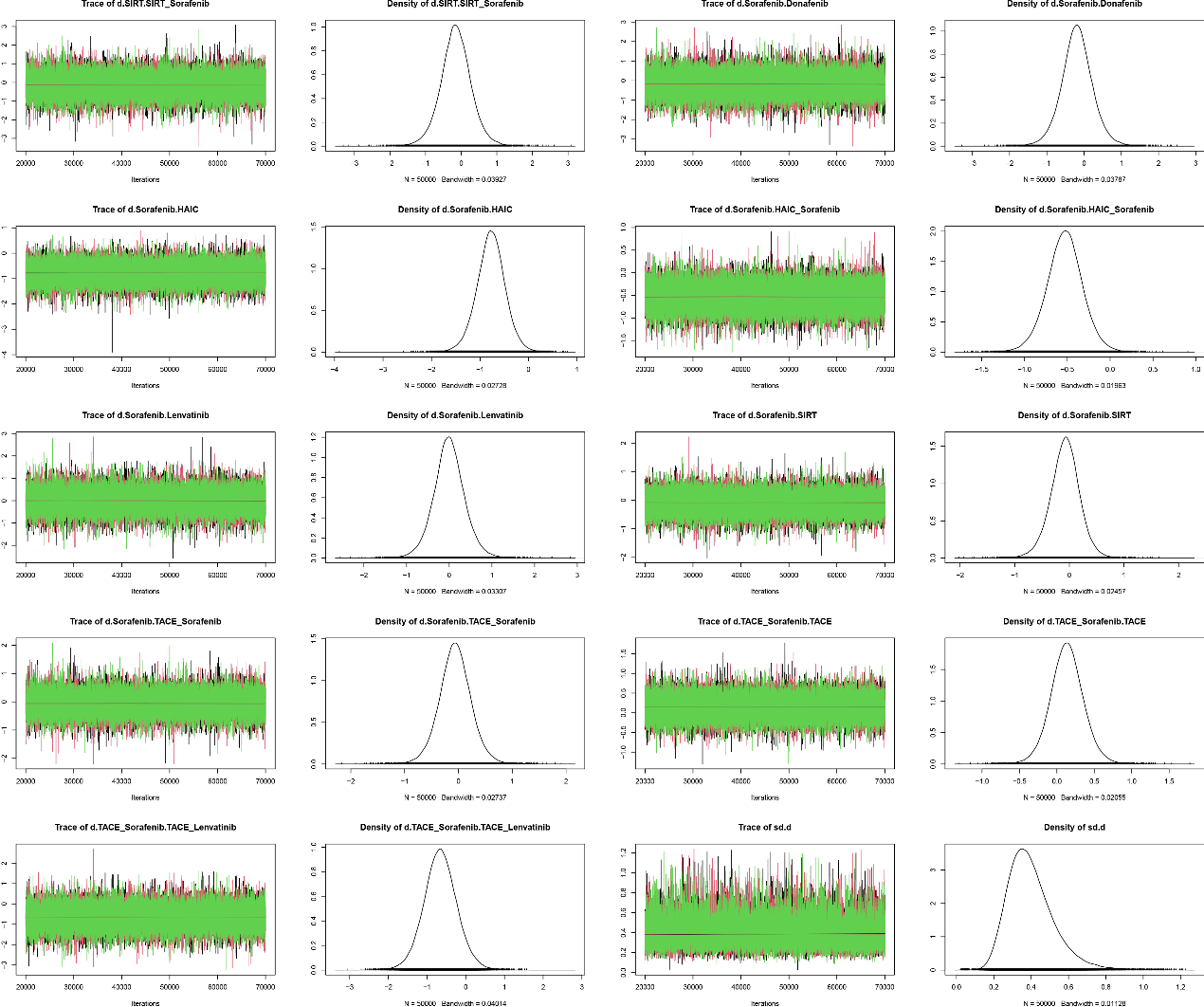


PFS
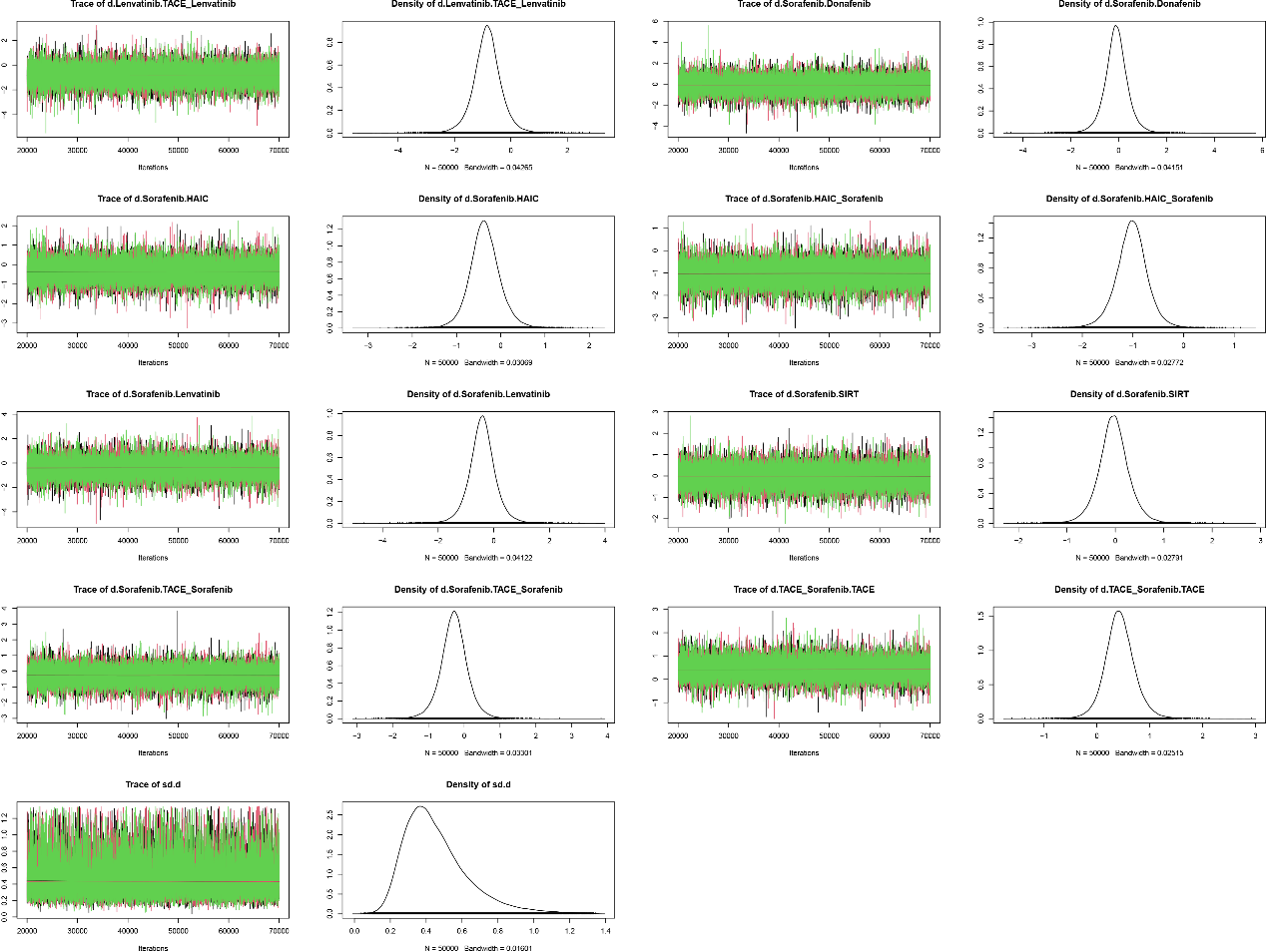


TTP
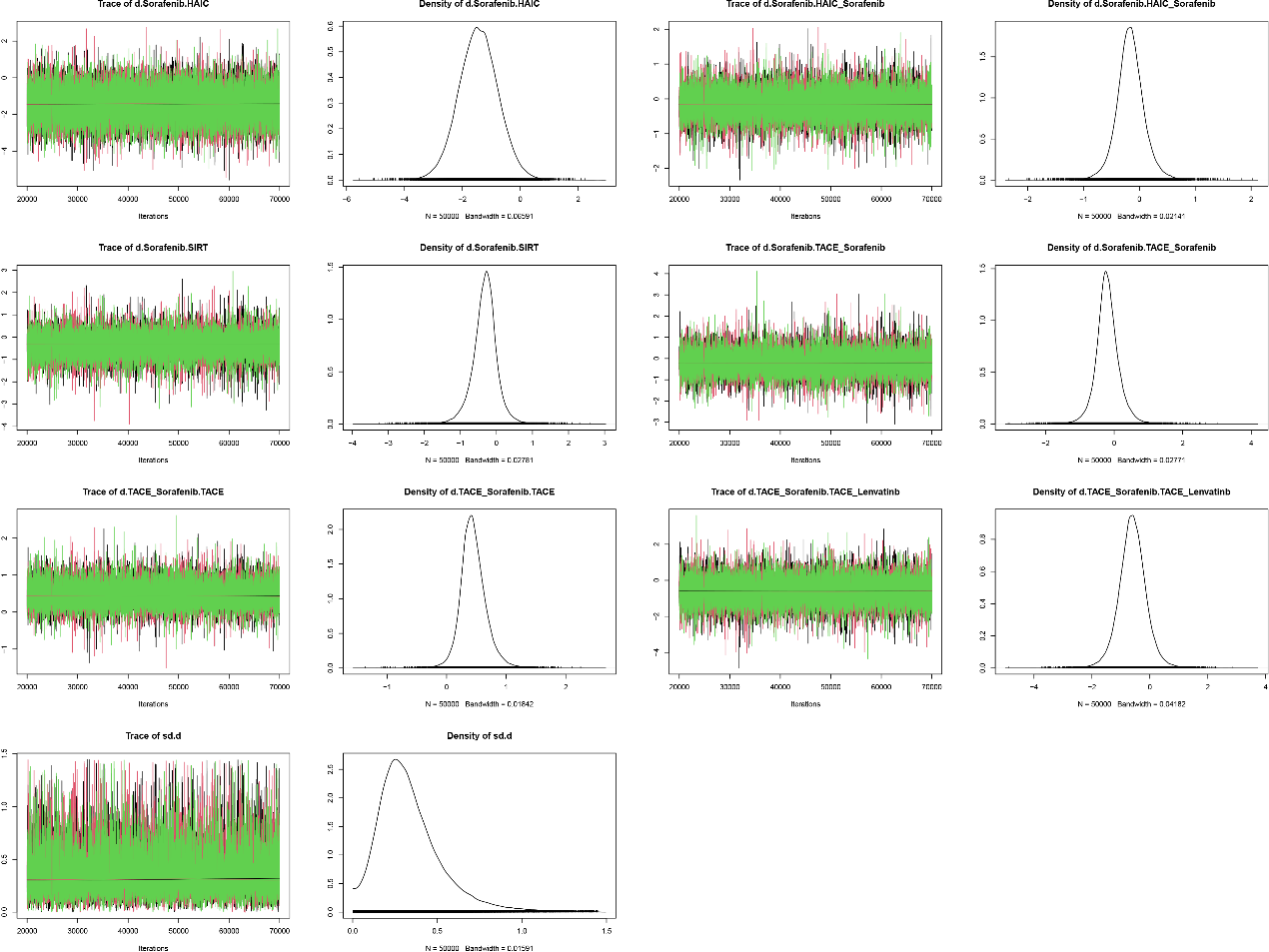


AE
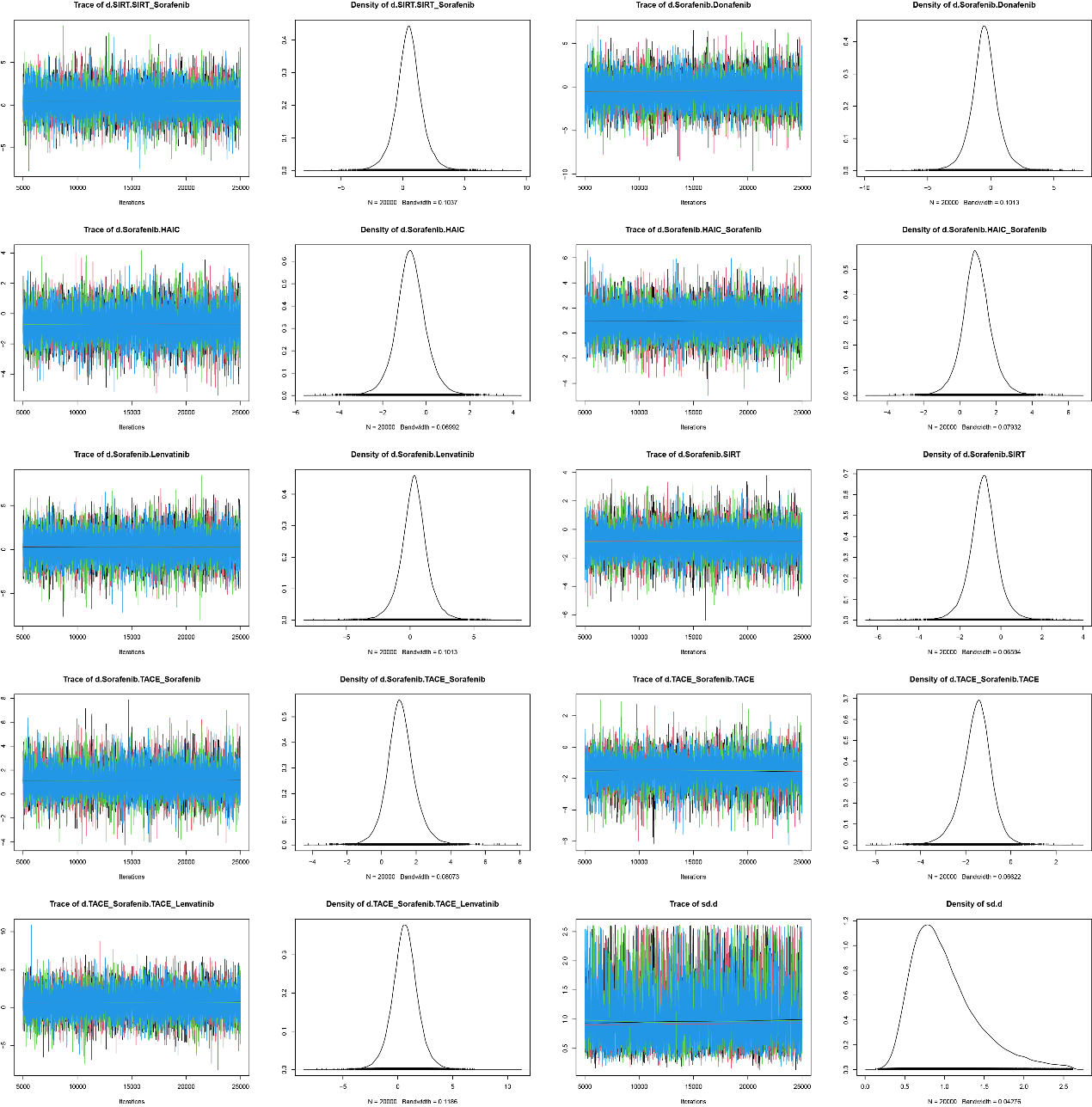


ORR(mRECIST)
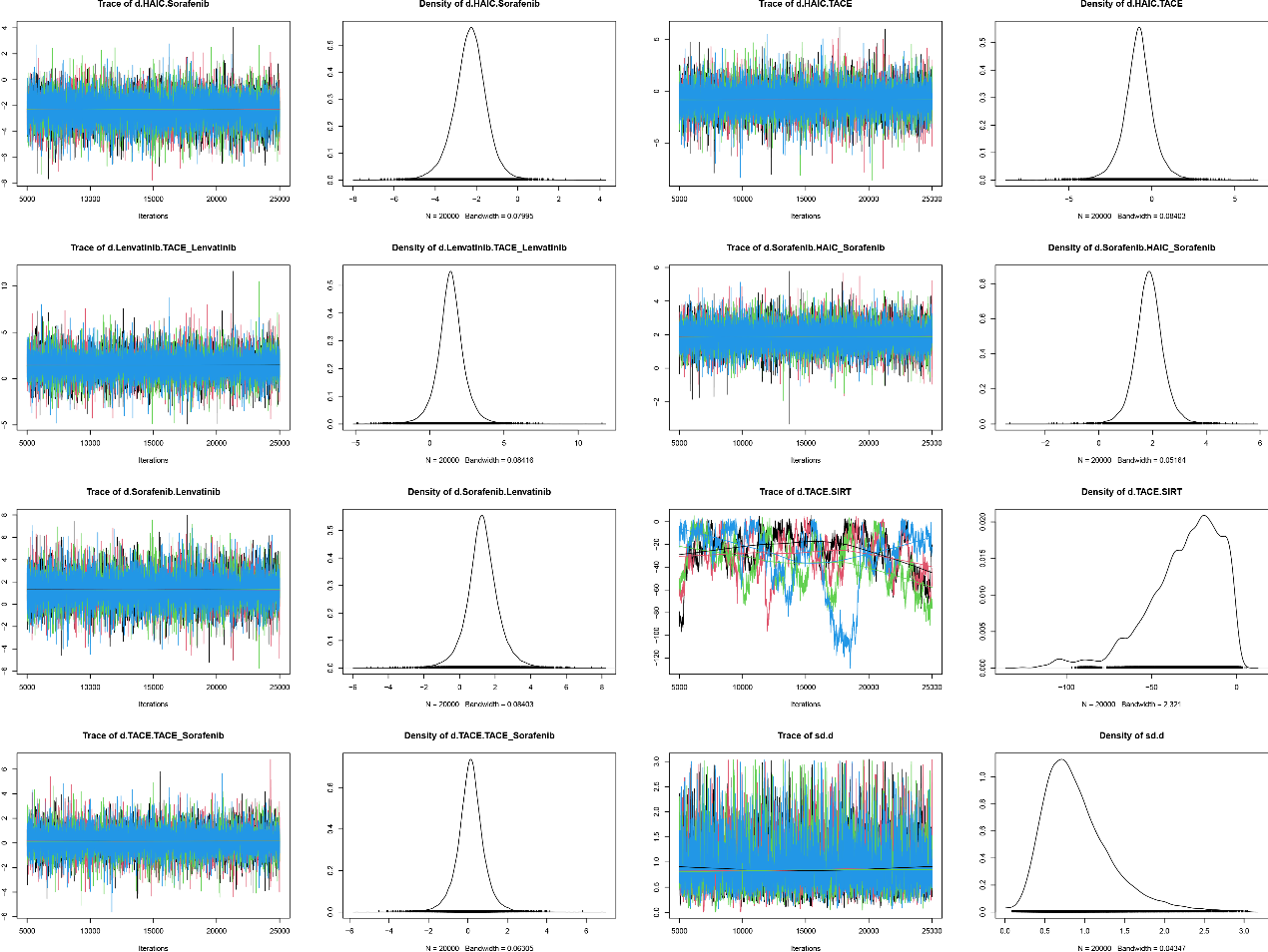


DCR(mRECIST)
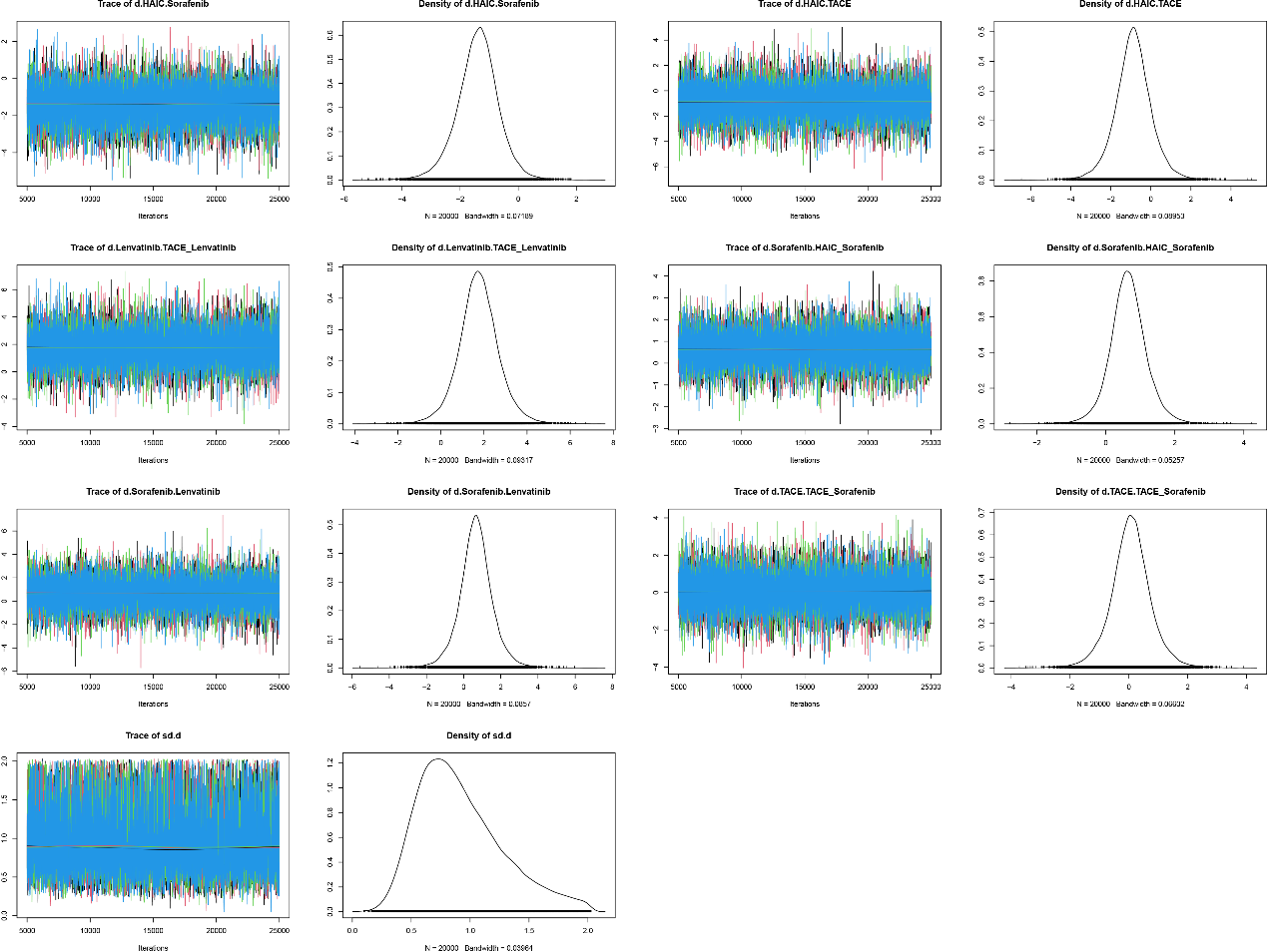


ORR(RECIST)
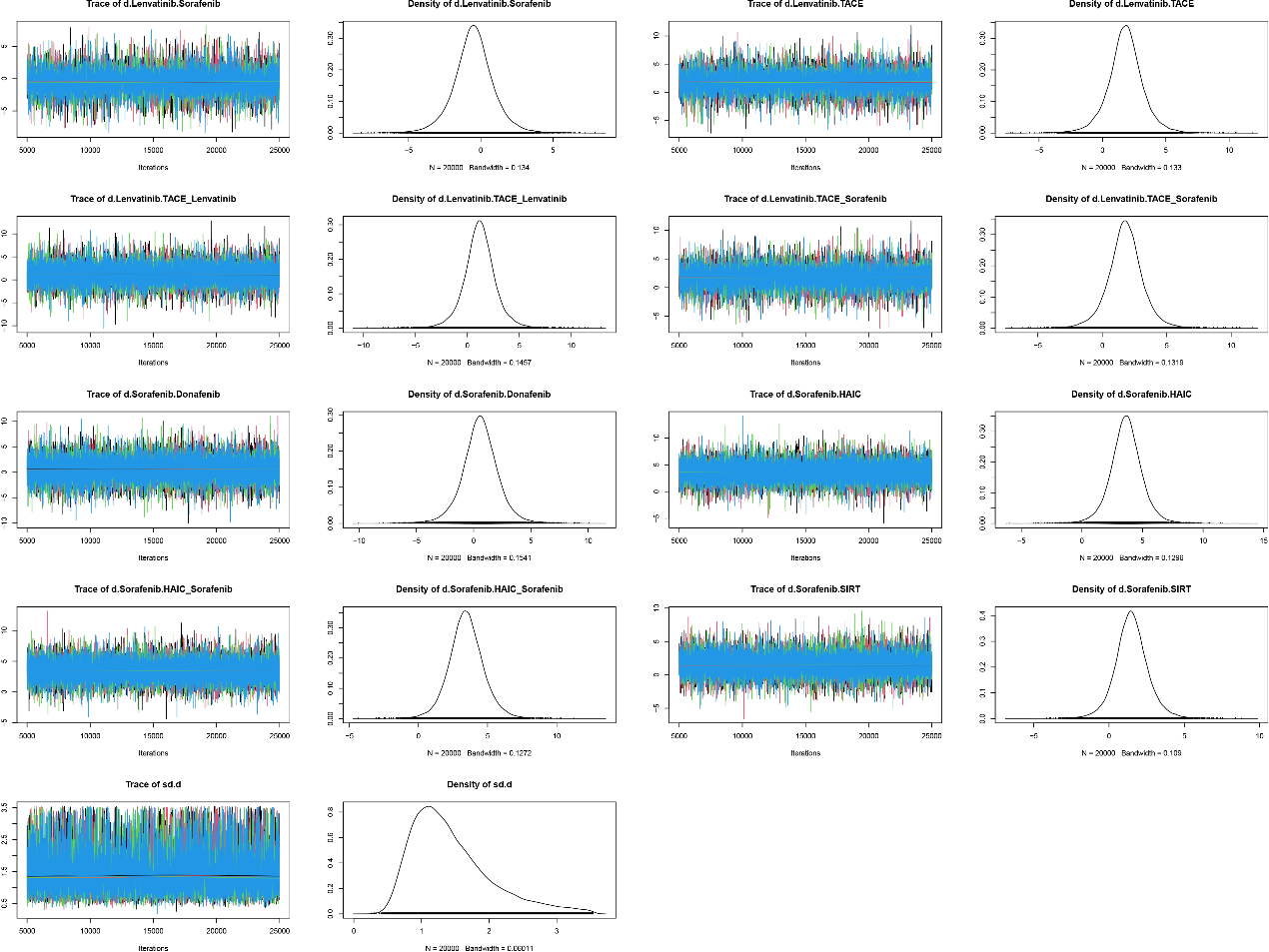


DCR(RECIST)
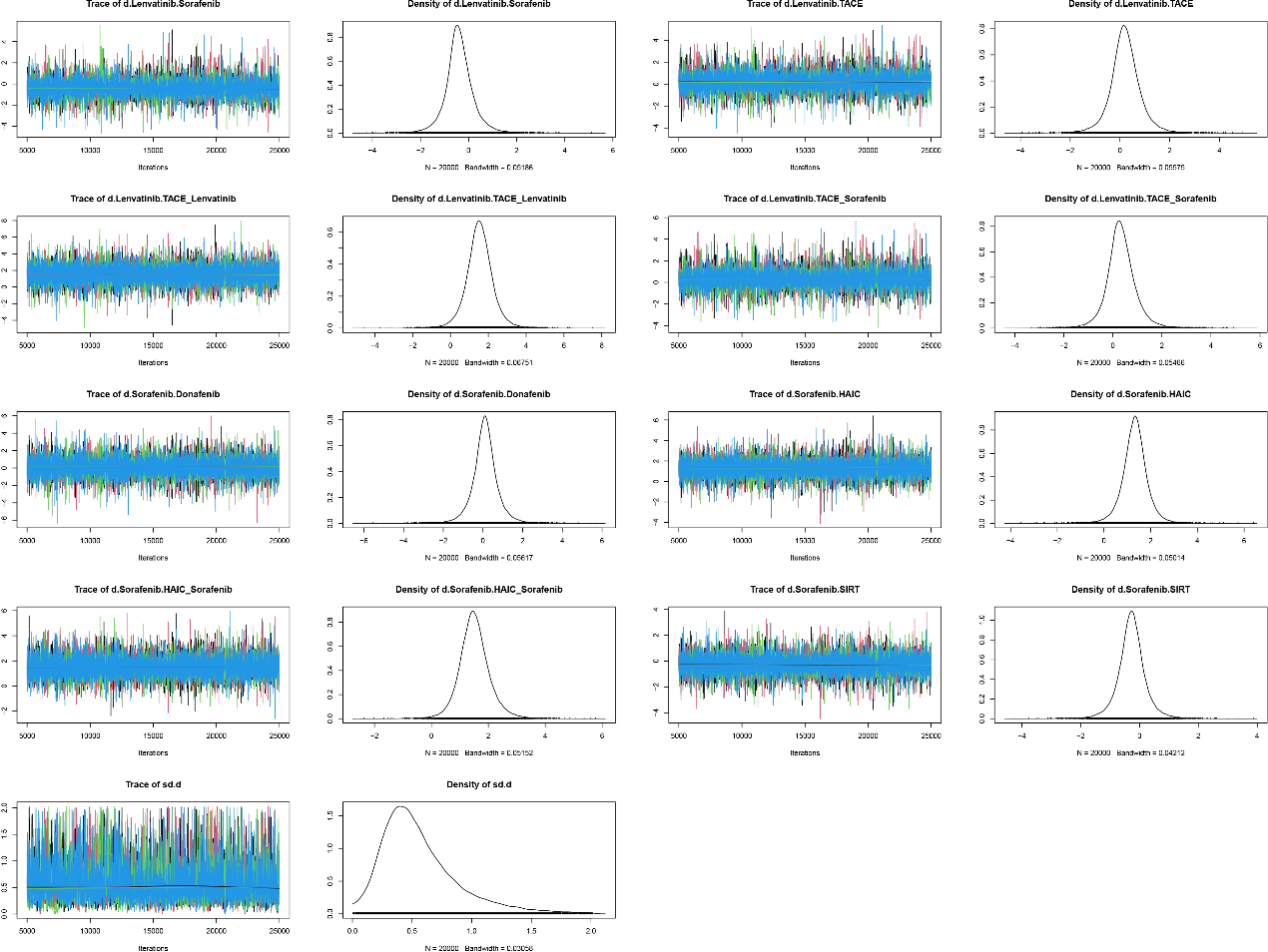


Supplementary Material S9. Results of publication bias (the Funnel Plot of Enrolled Trials)

OS
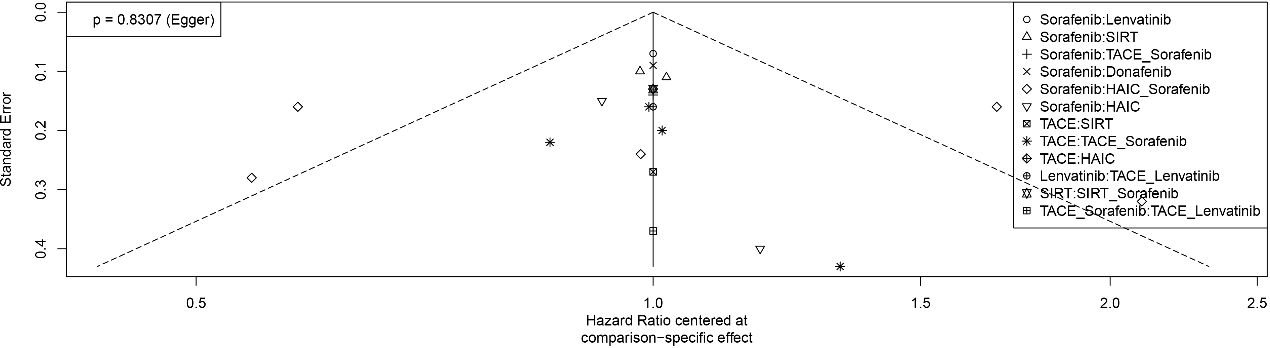


PFS
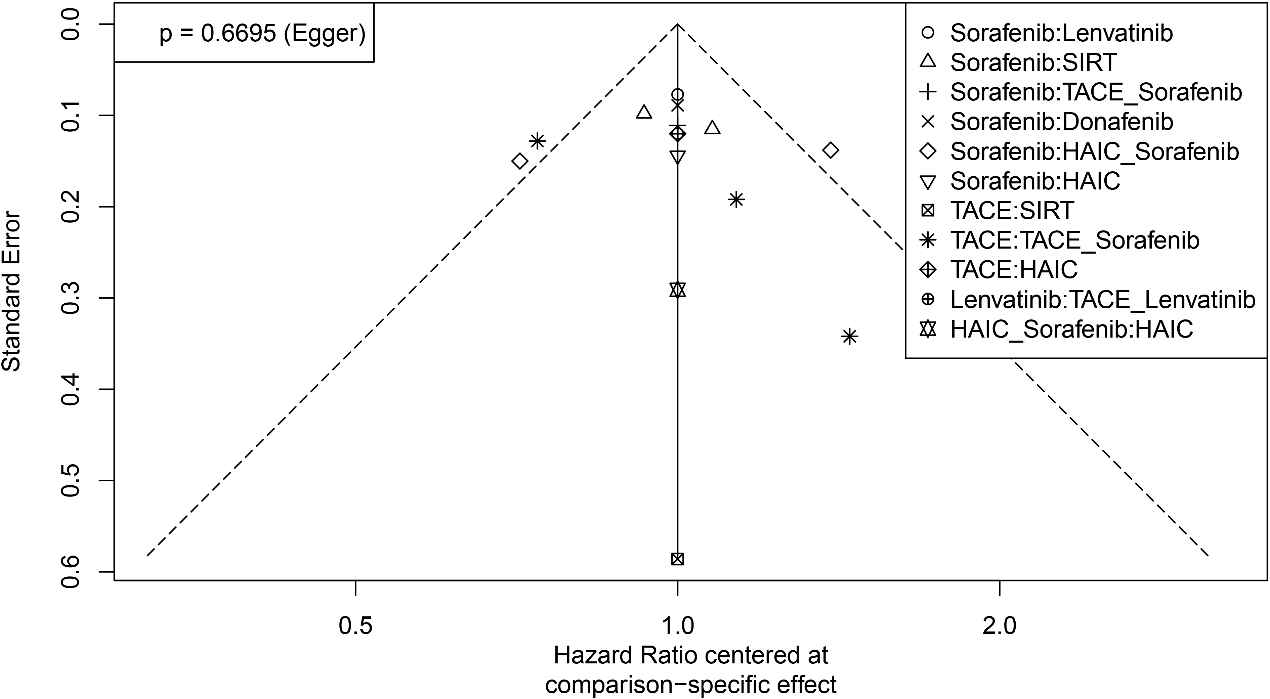


TTP
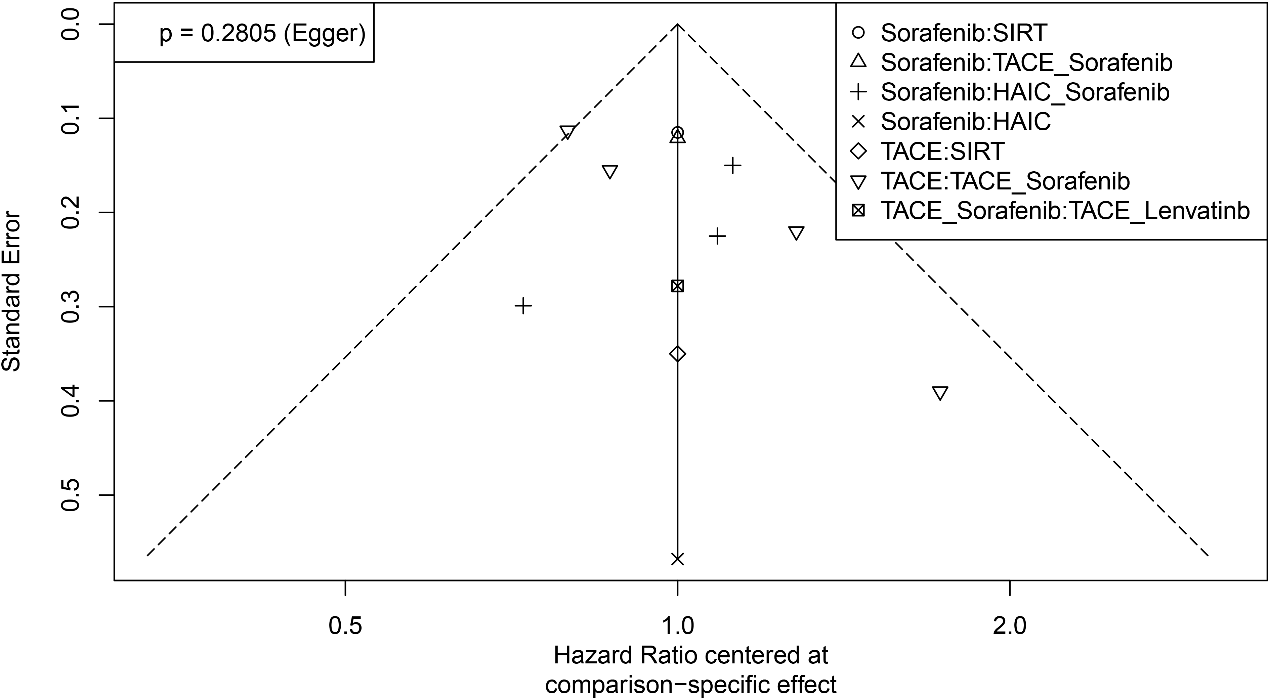


AE
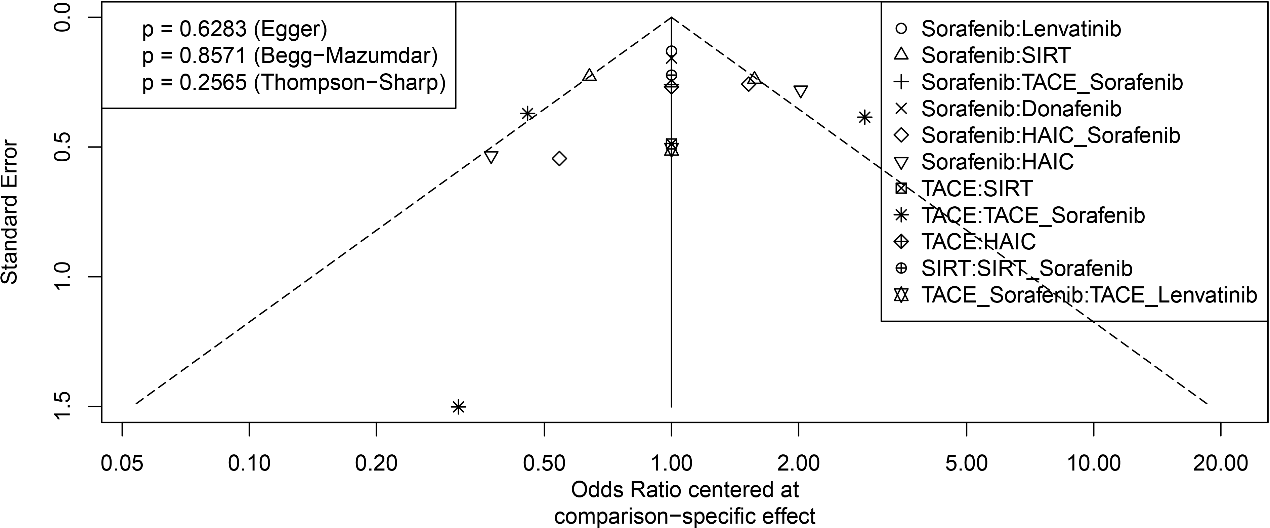


ORR(mRECIST)
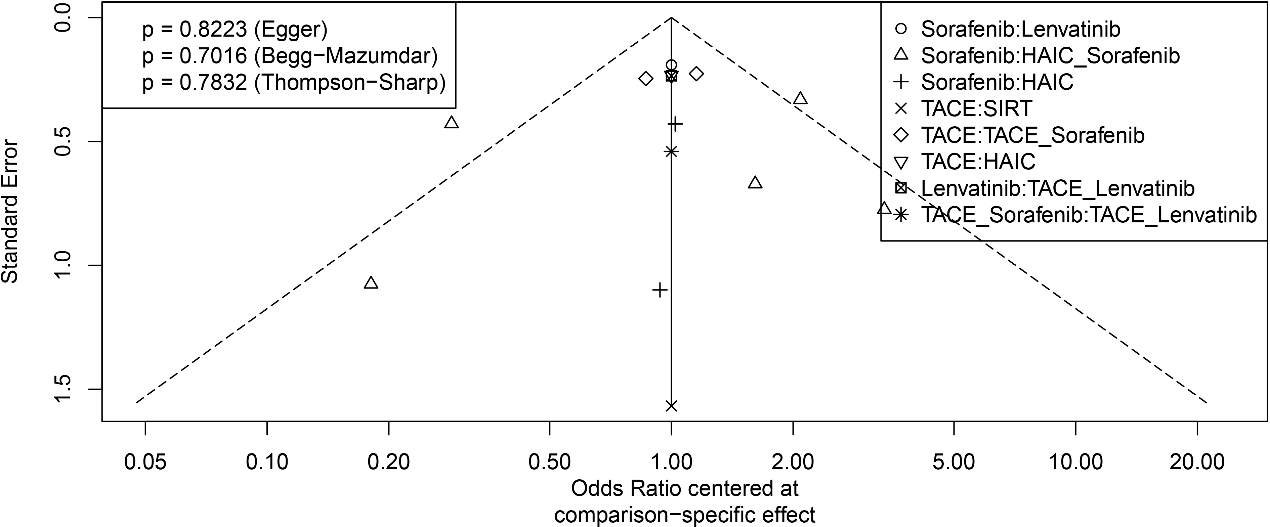


DCR(mRECIST)
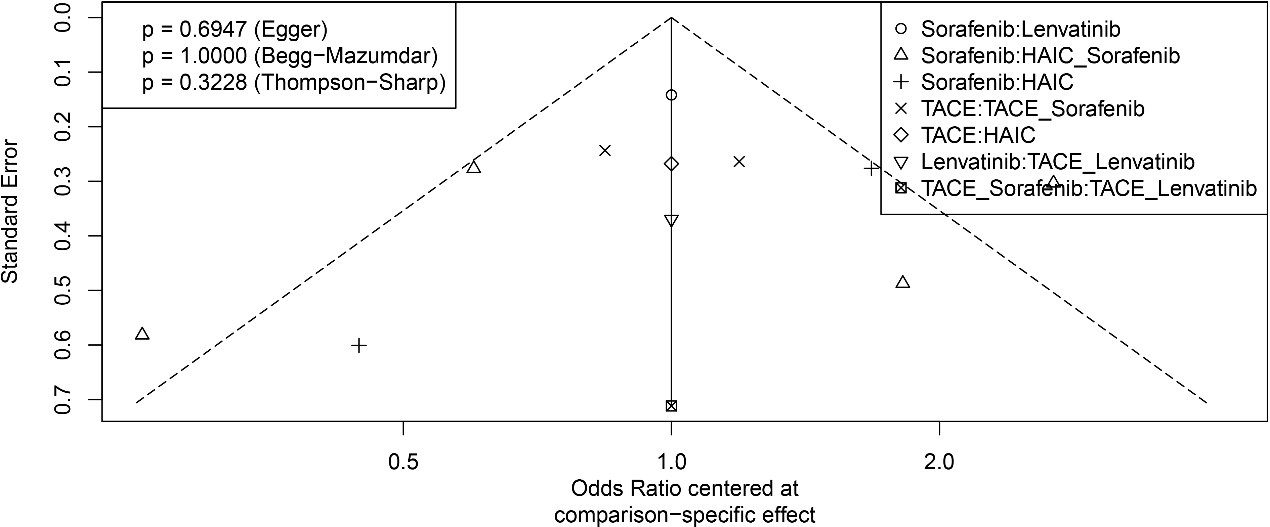


ORR(RECIST)
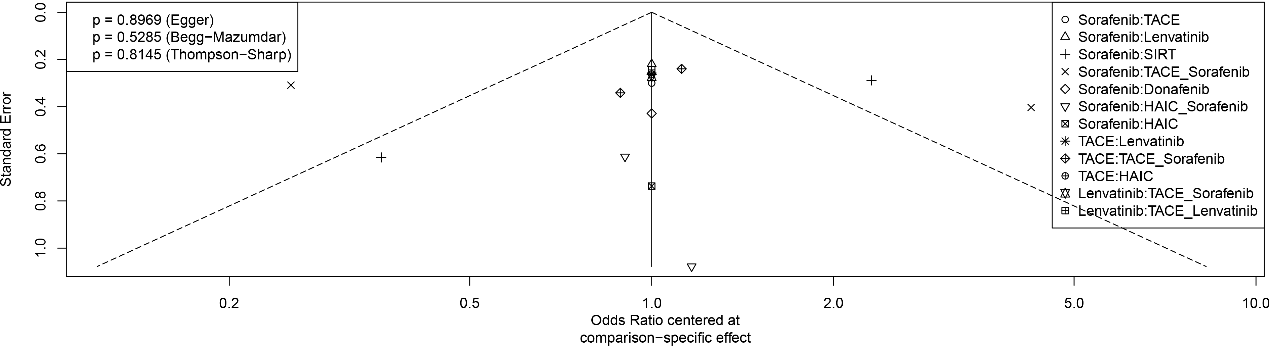


DCR(RECIST)
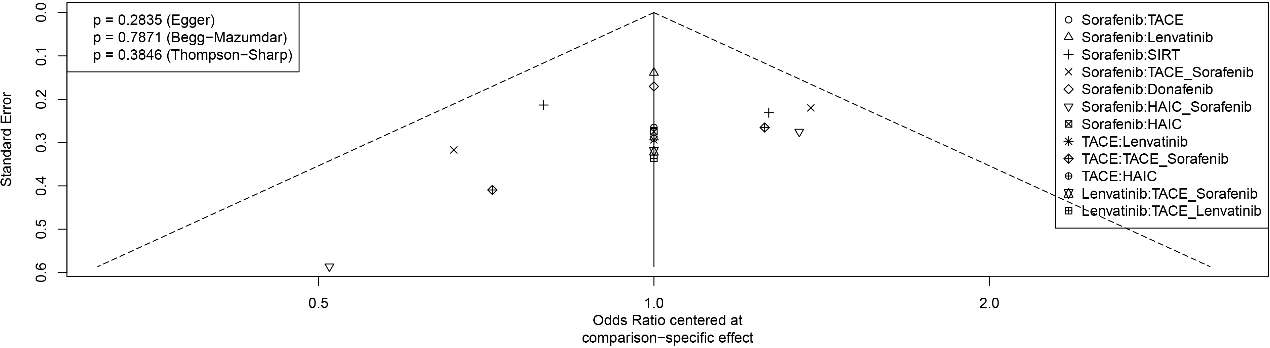


Supplementary Material S10. Node-splitting method for assessing local inconsistency between direct and indirect evidence

A node-splitting method was used to check the local consistency between direct and indirect evidence, and the geometry of the treatment network was established using R software.

OS
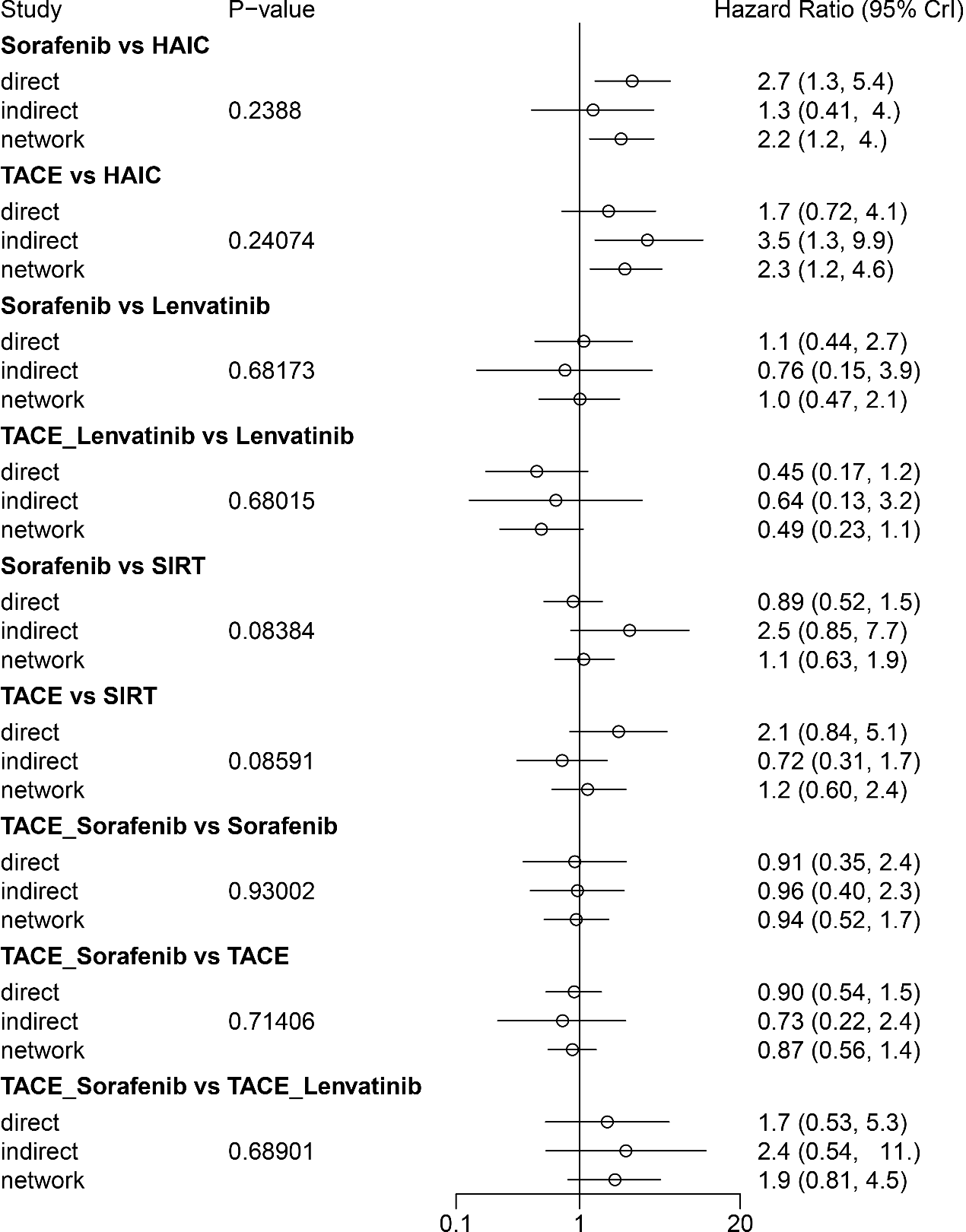


PFS
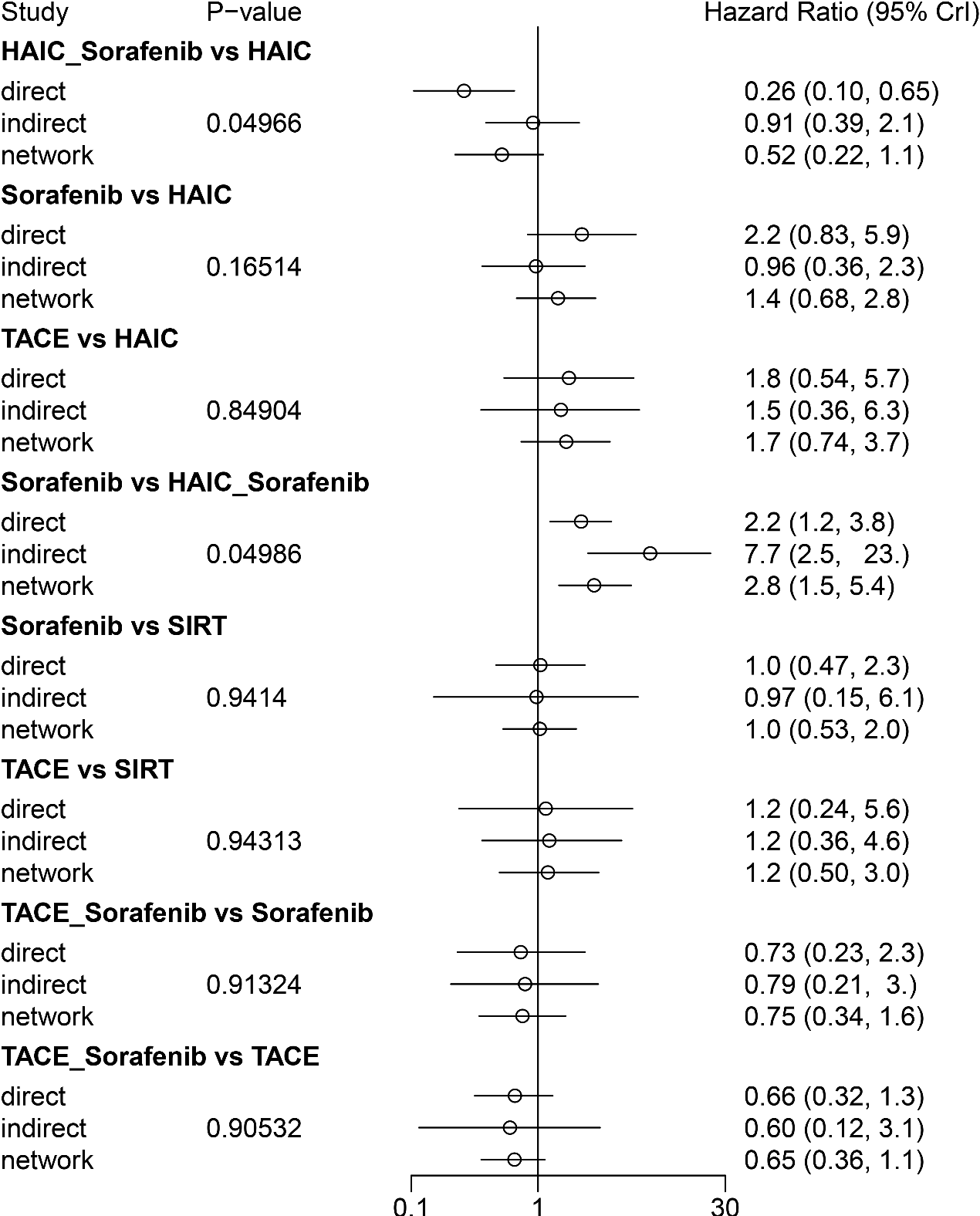


TTP
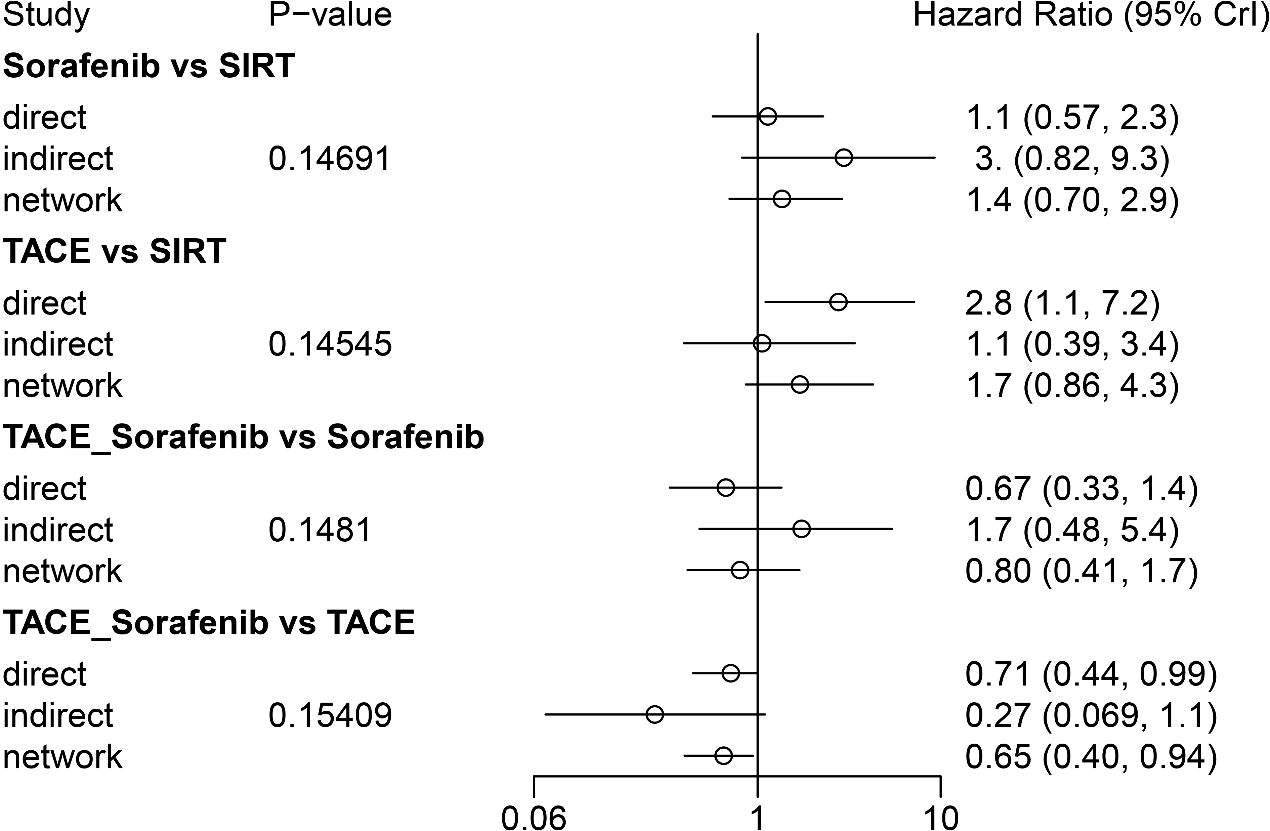


AE
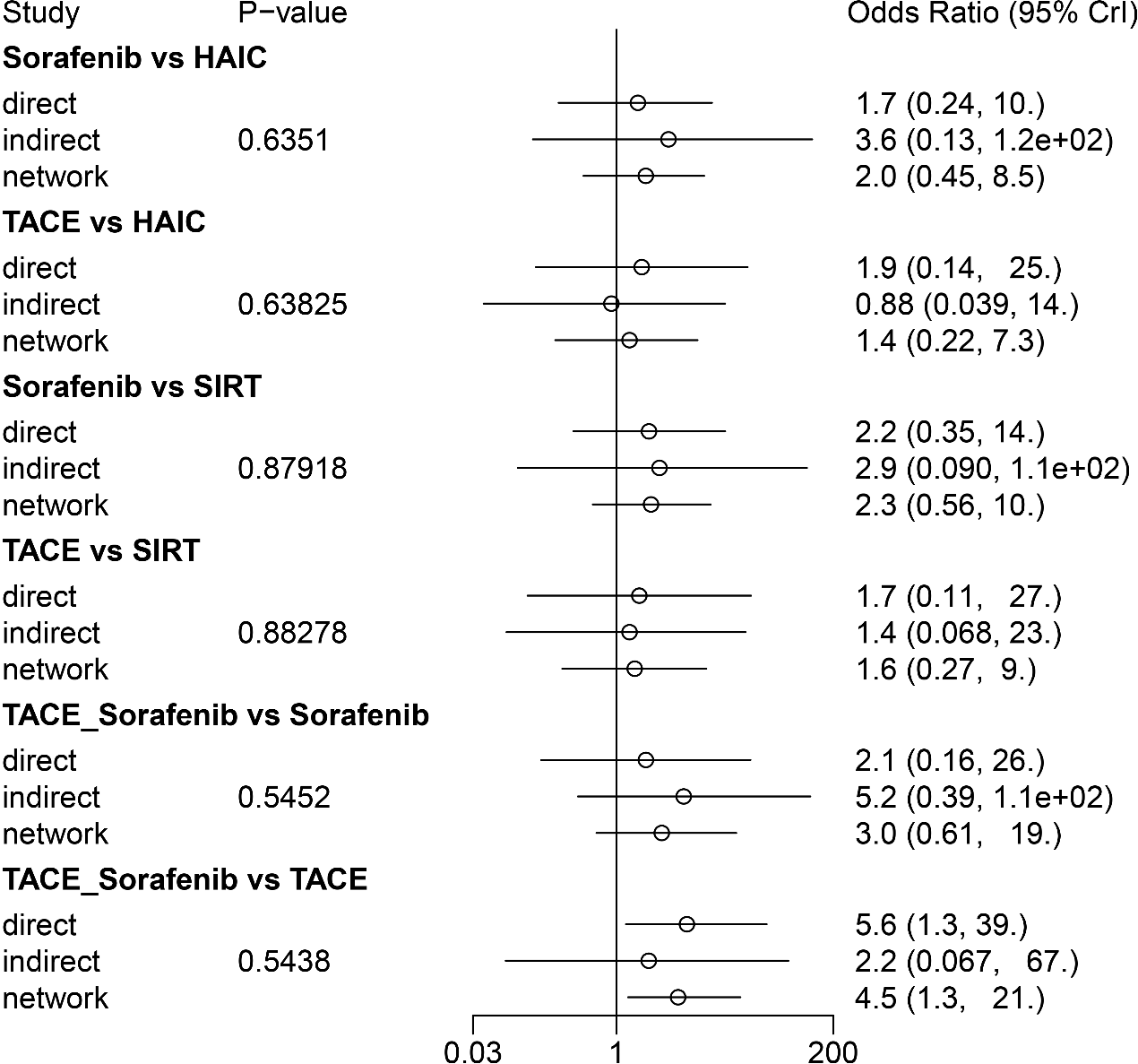


ORR(mRECIST) )
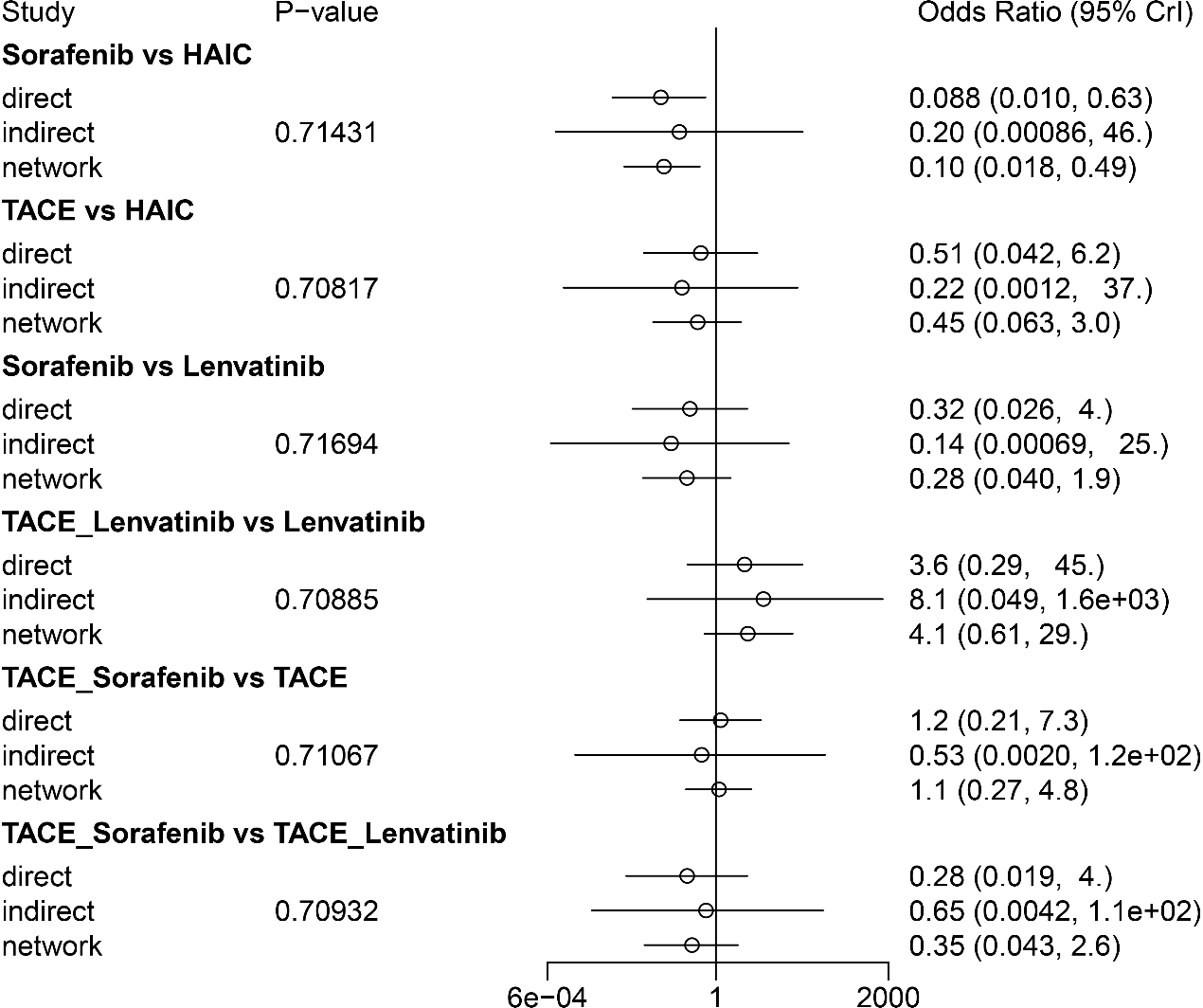


DCR(mRECIST)
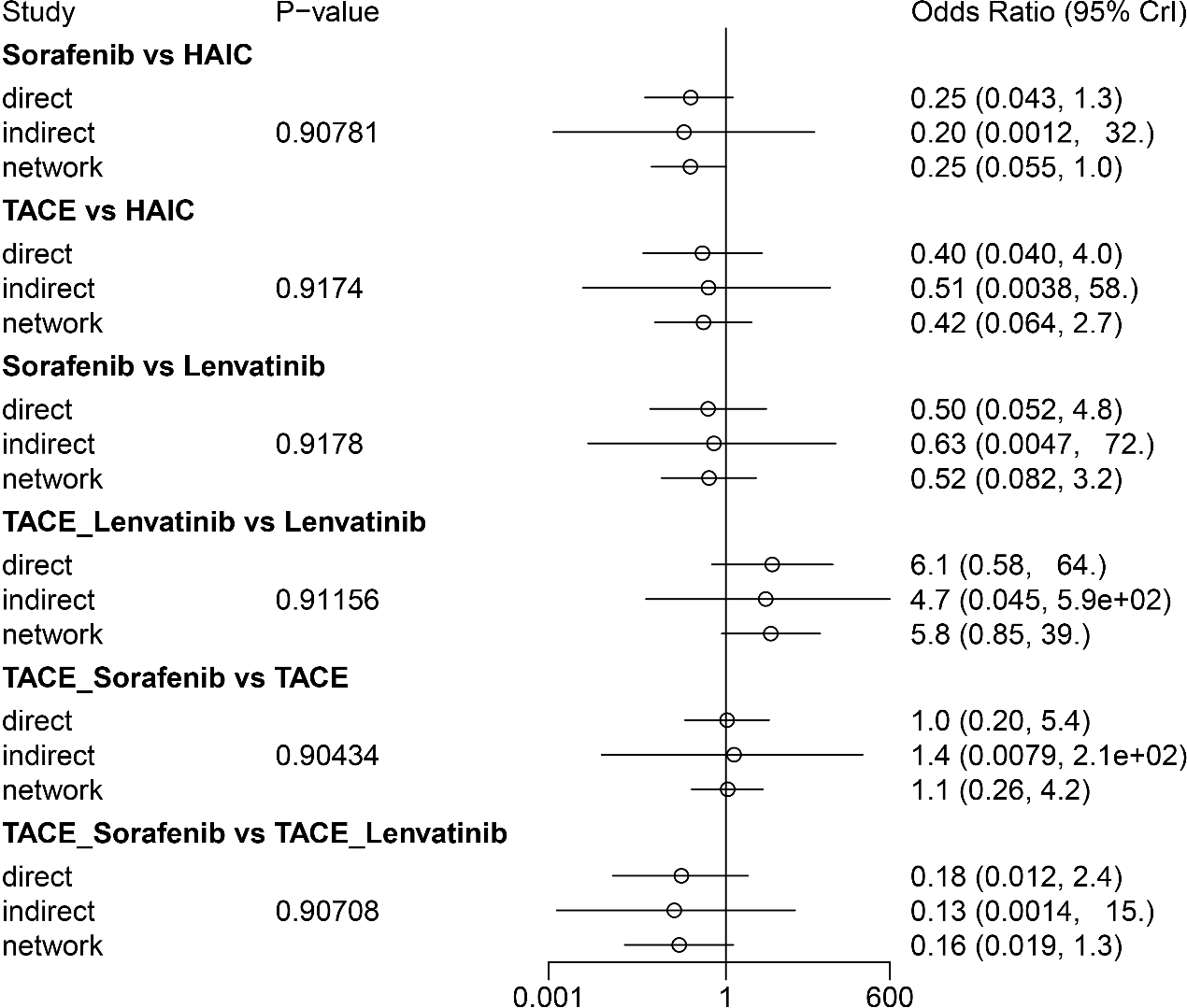


ORR(RECIST)
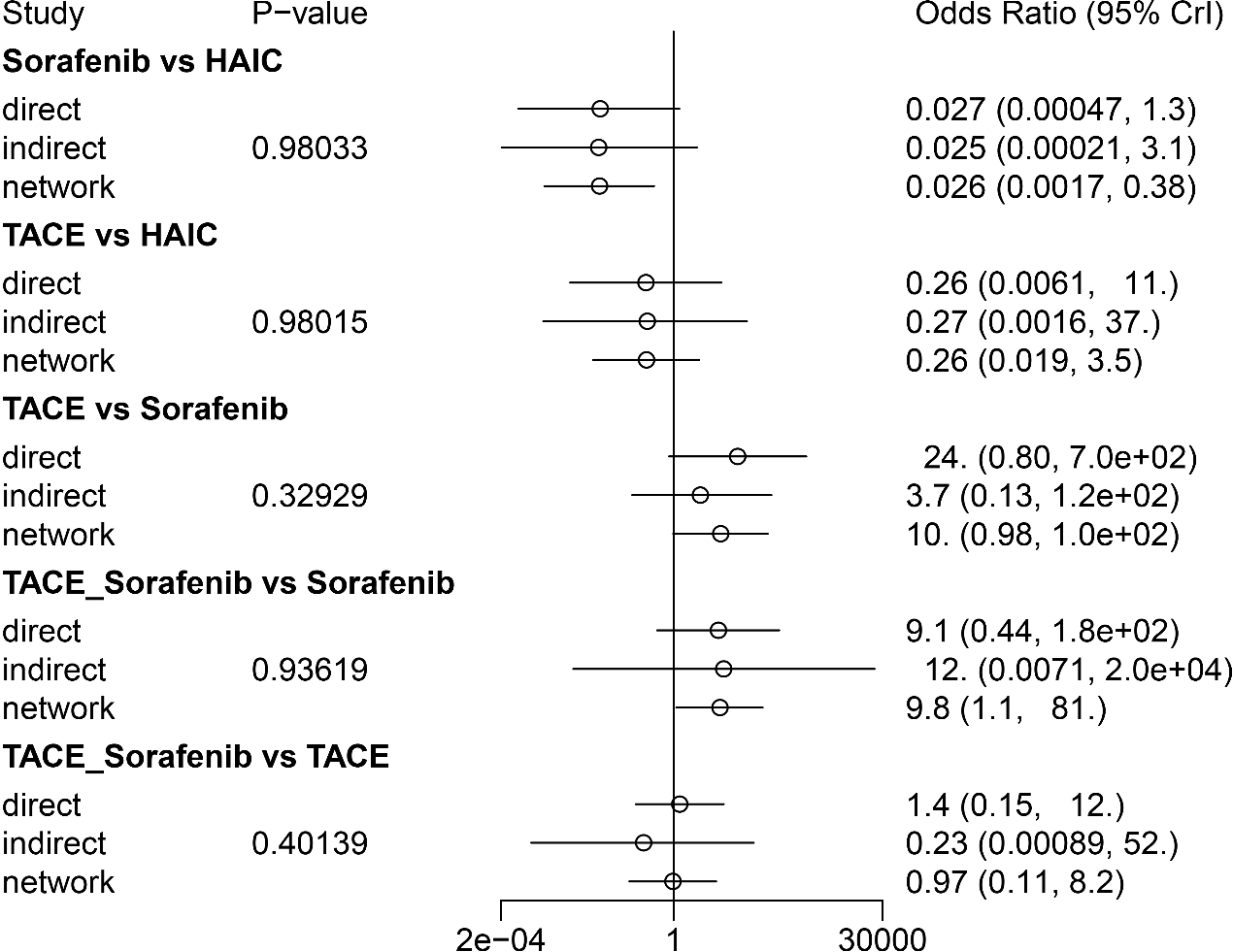


DCR(RECIST)
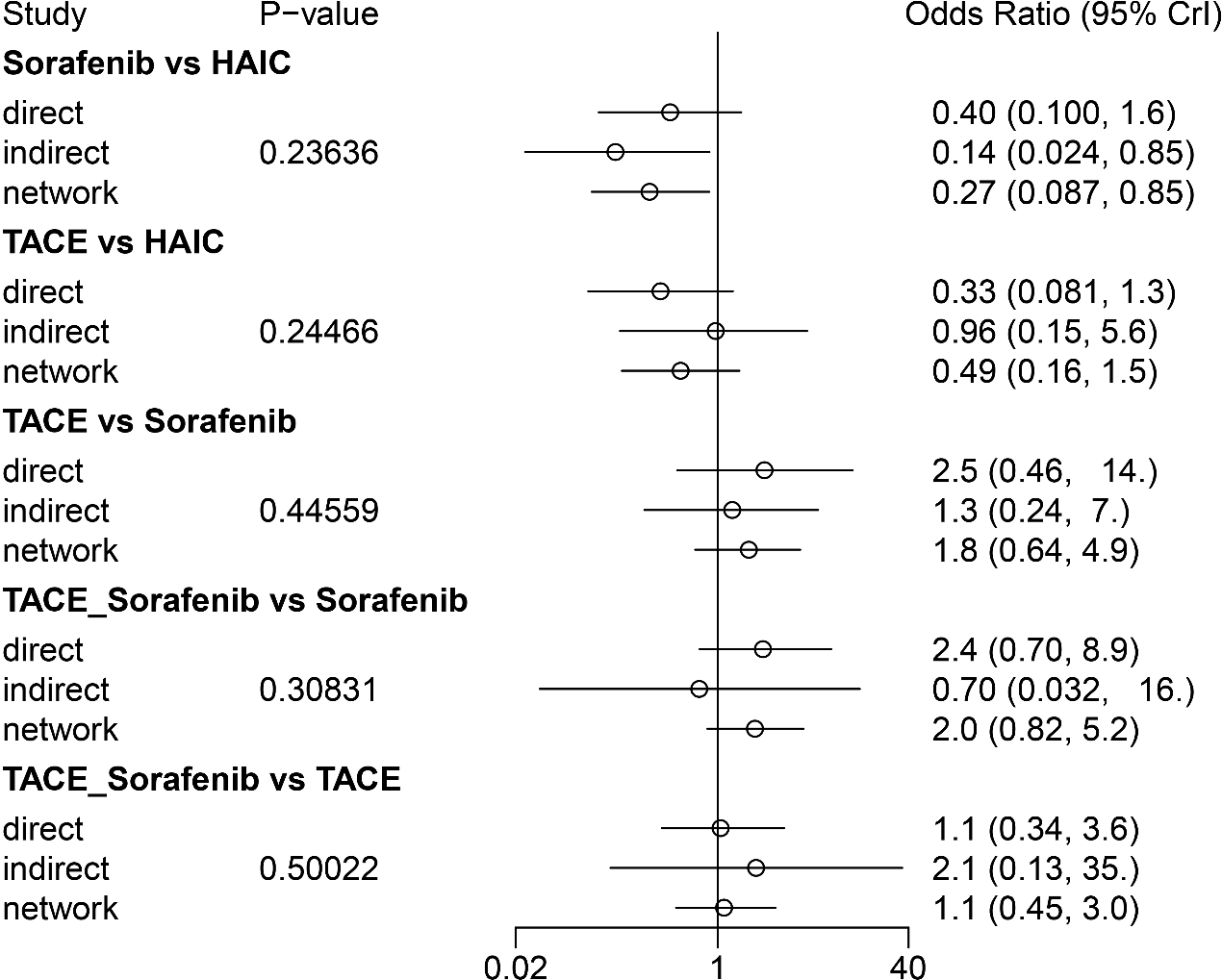
Supplementary Material S11. Heterogeneity.

The “mtc.anohe” command in the “gemtc” package was used to evaluate heterogeneity, which was documented using the variance parameter I^2^. Further,a value >50% was considered indicative of considerable heterogeneity.

OS


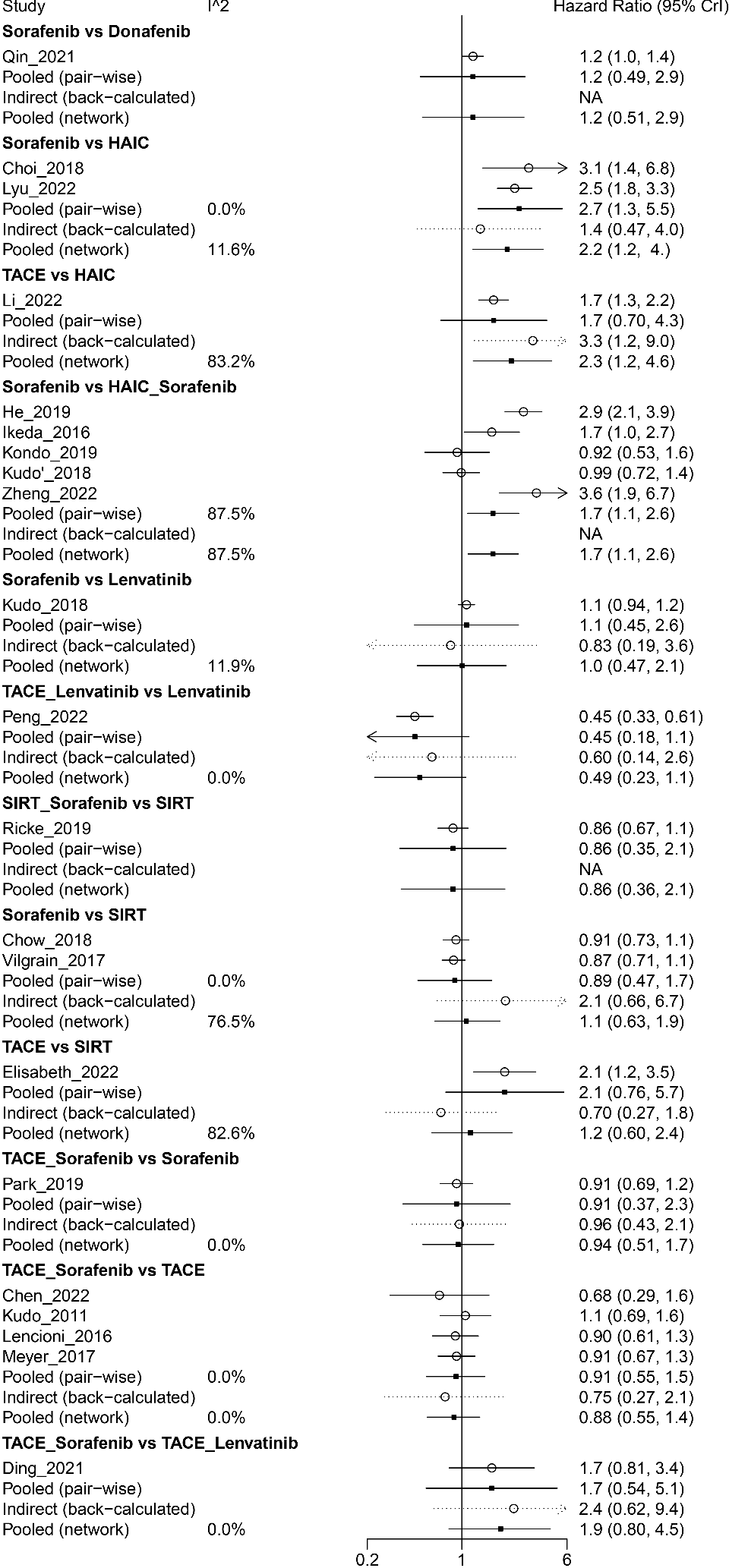


PFS


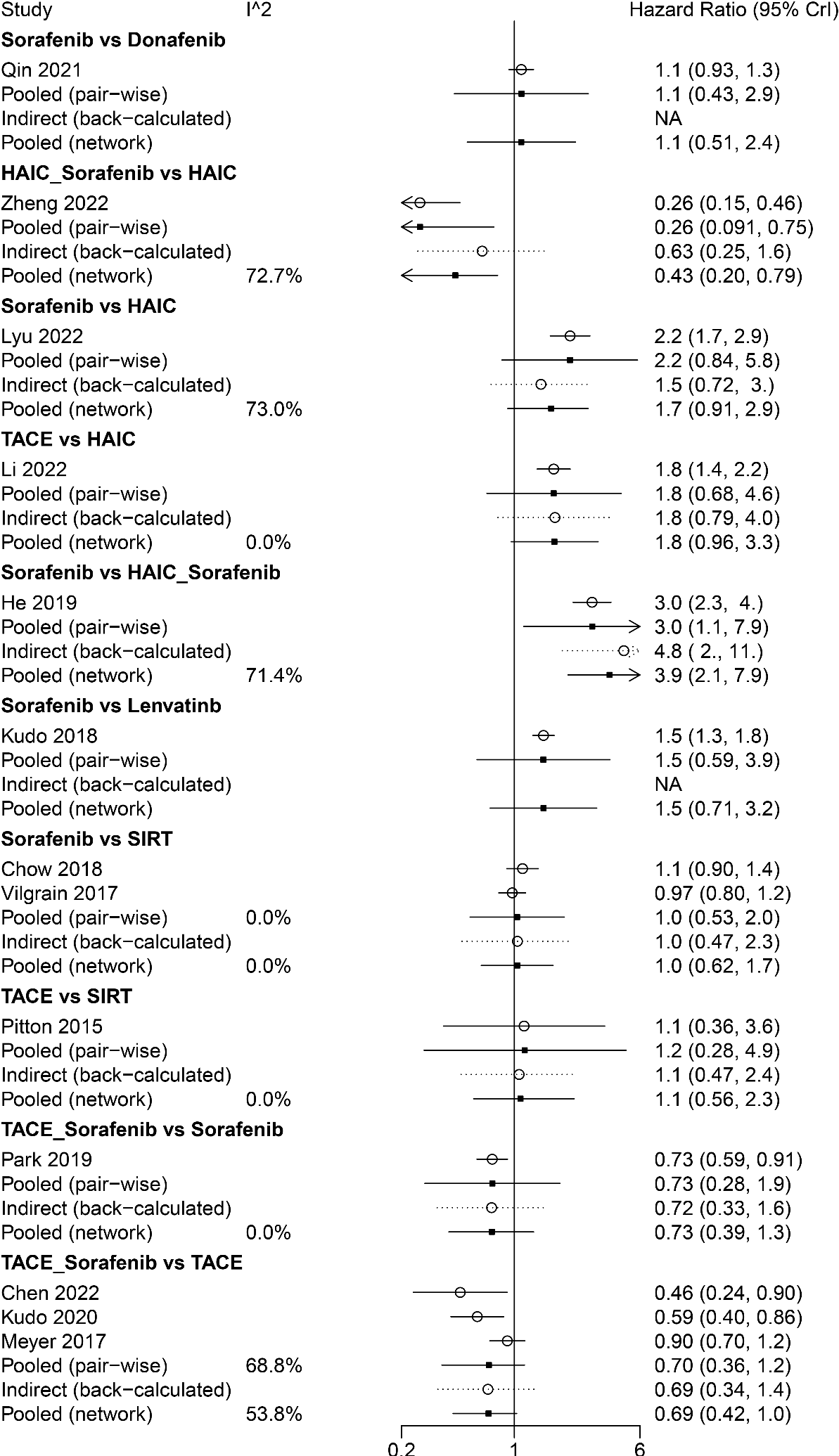


TTP


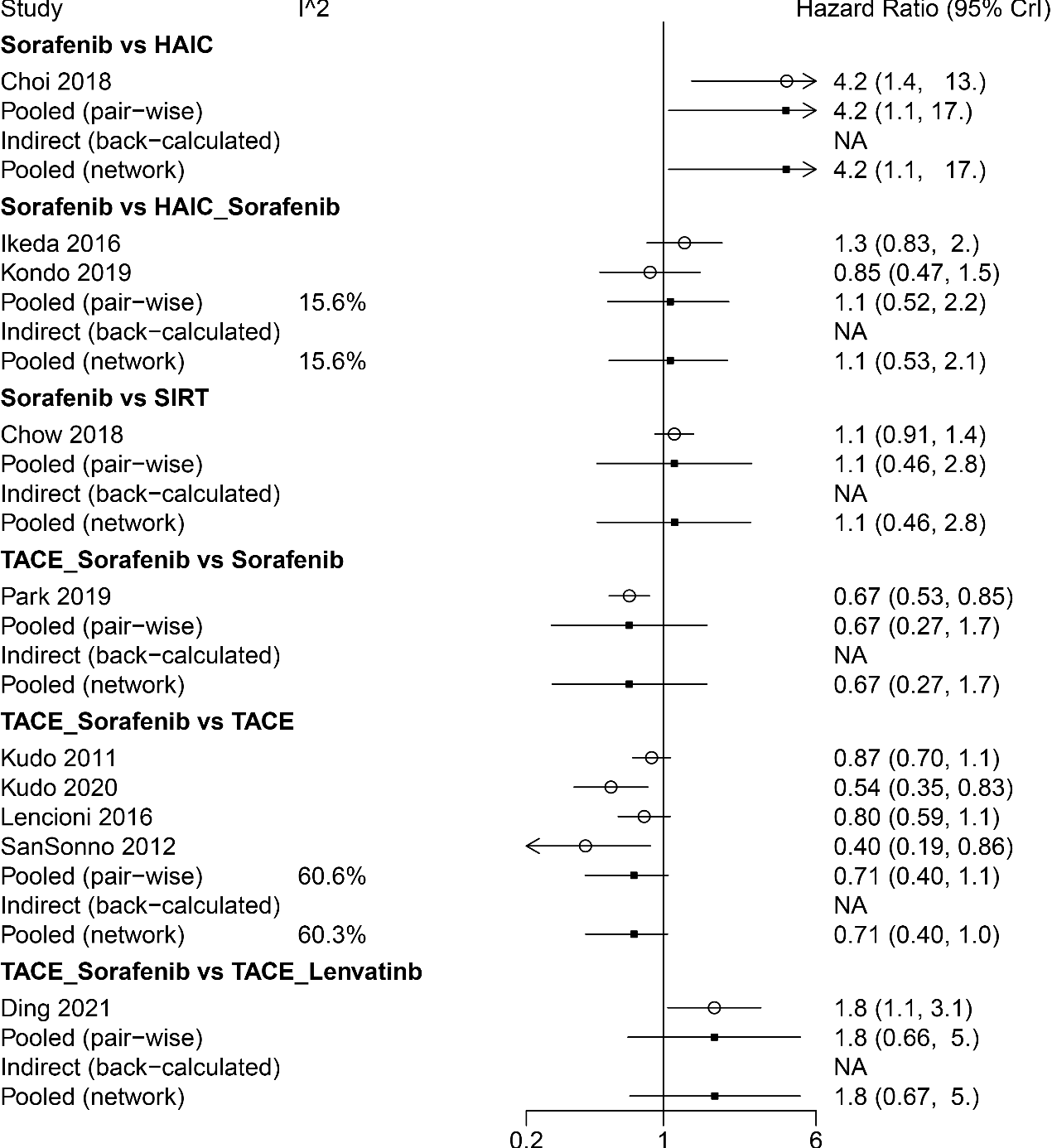


AE


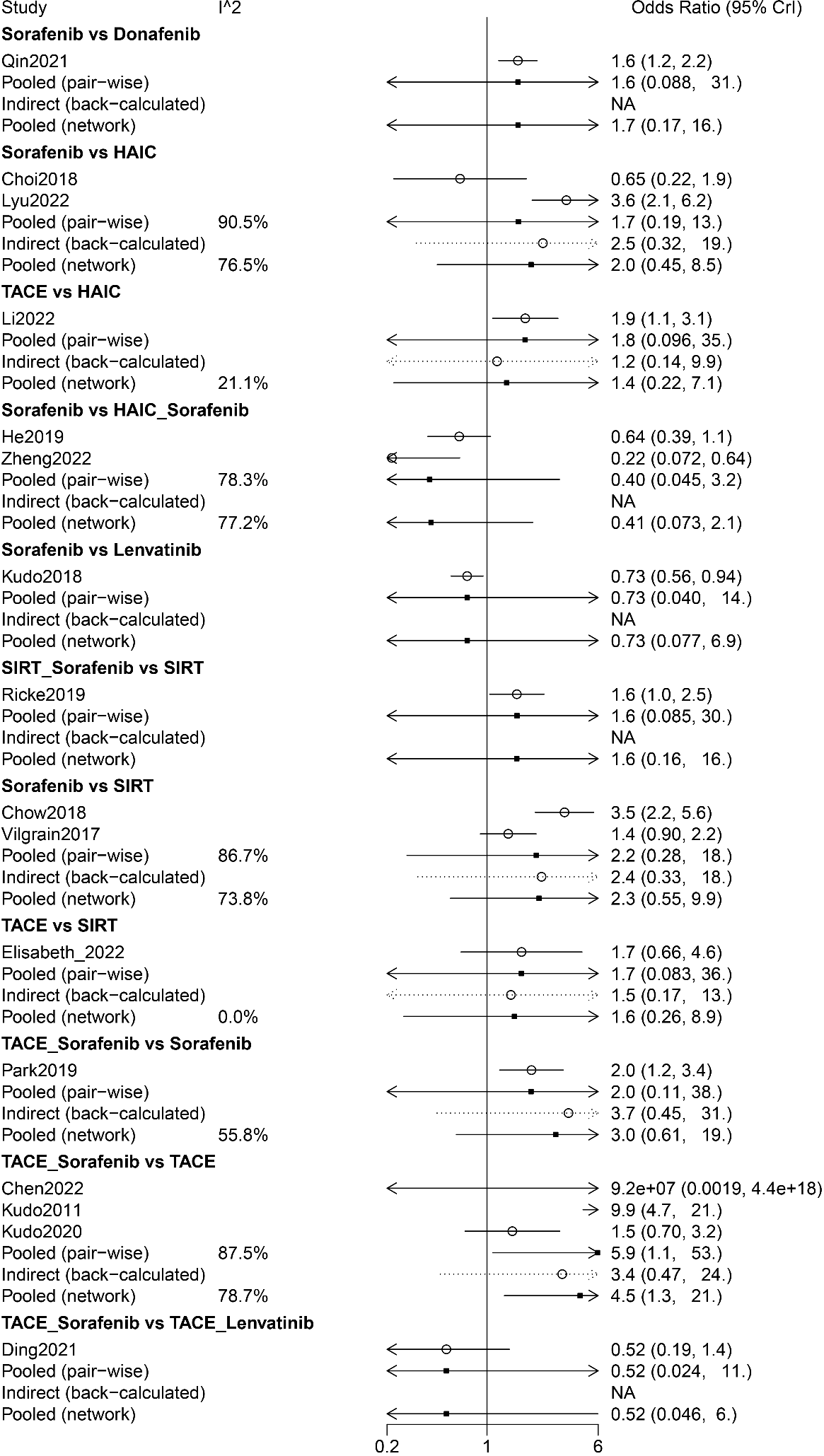


ORR(mRECIST)


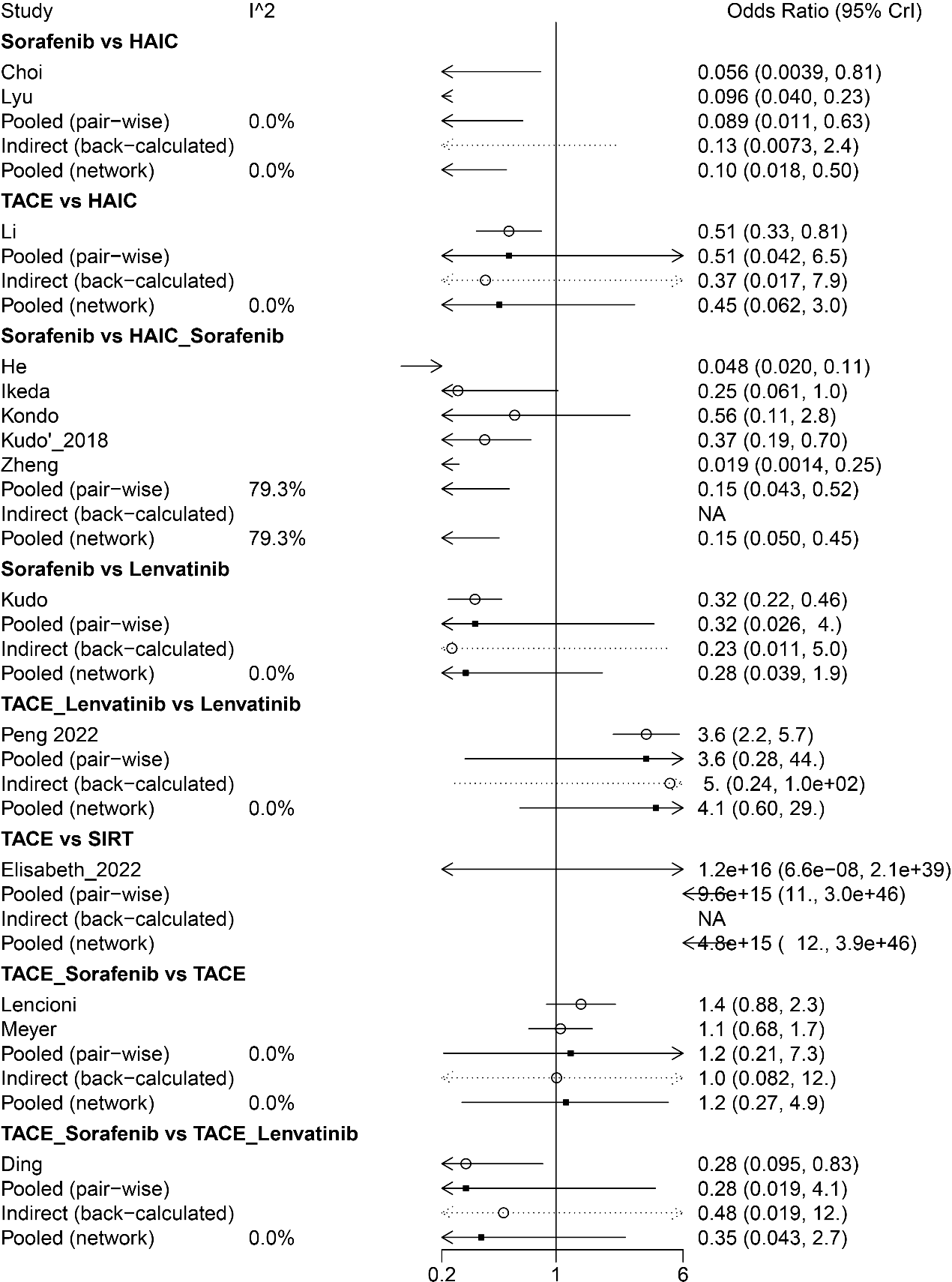


DCR(mRECIST)


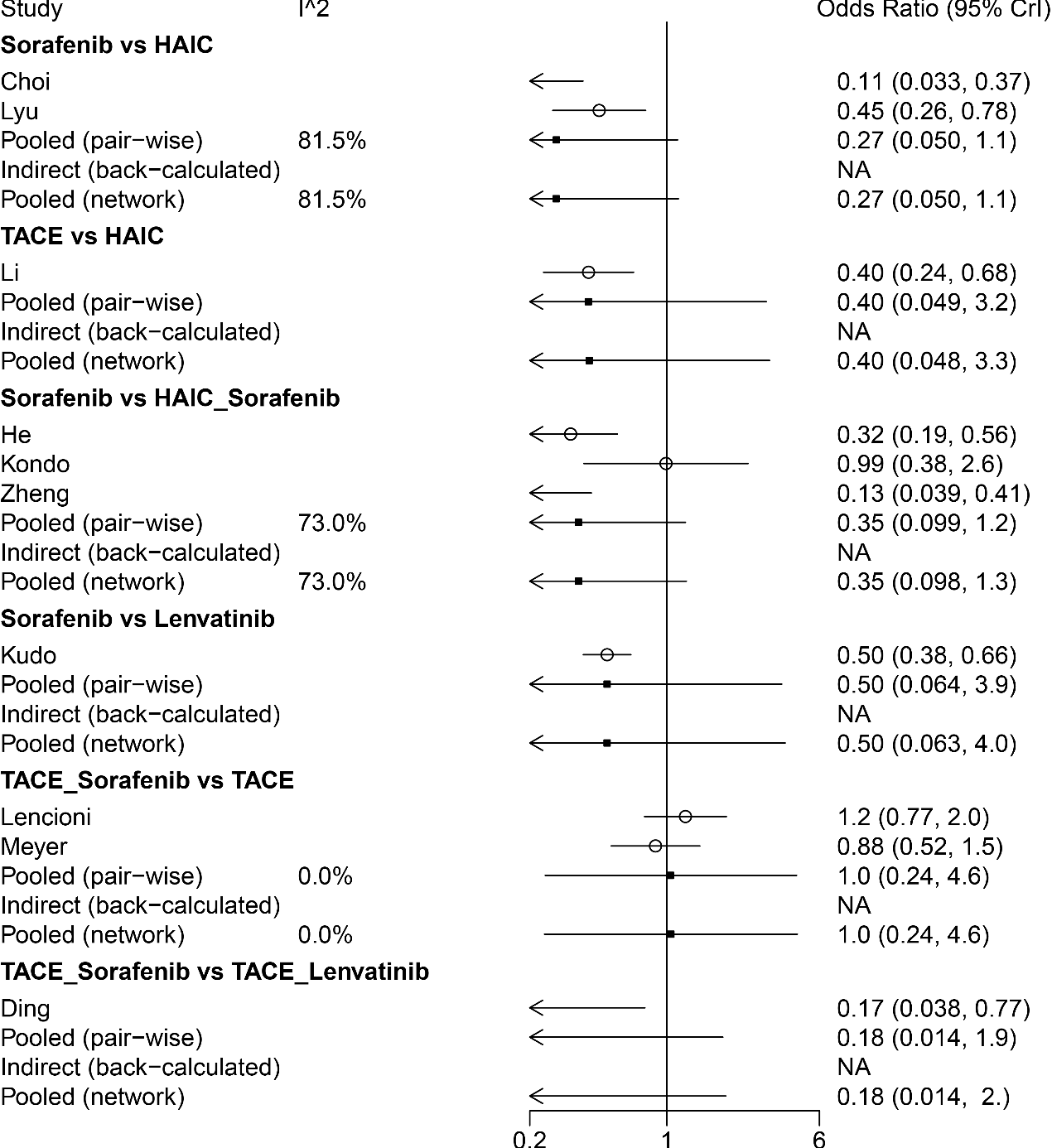


ORR(RECIST)


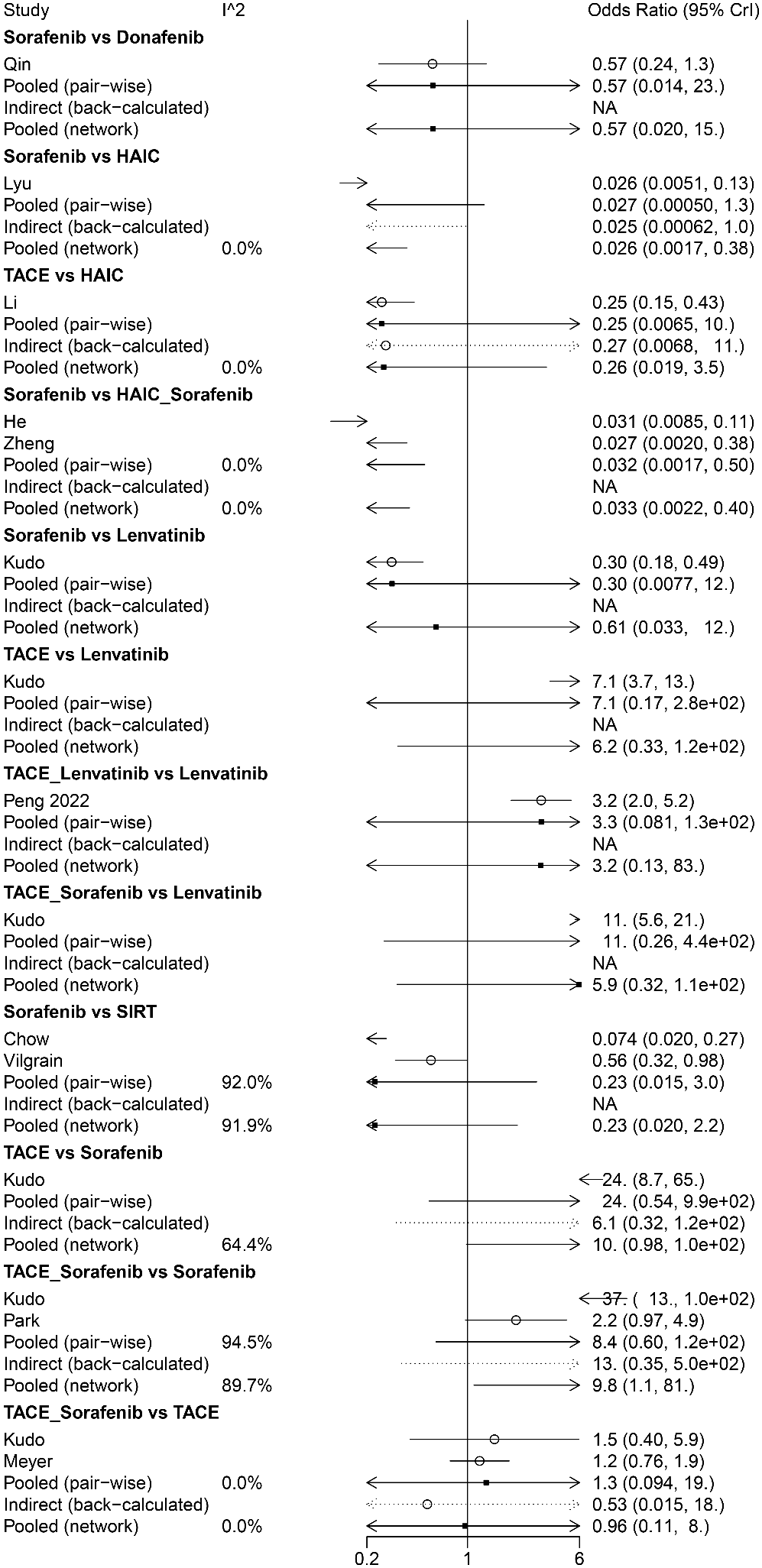


DCR(RECIST)


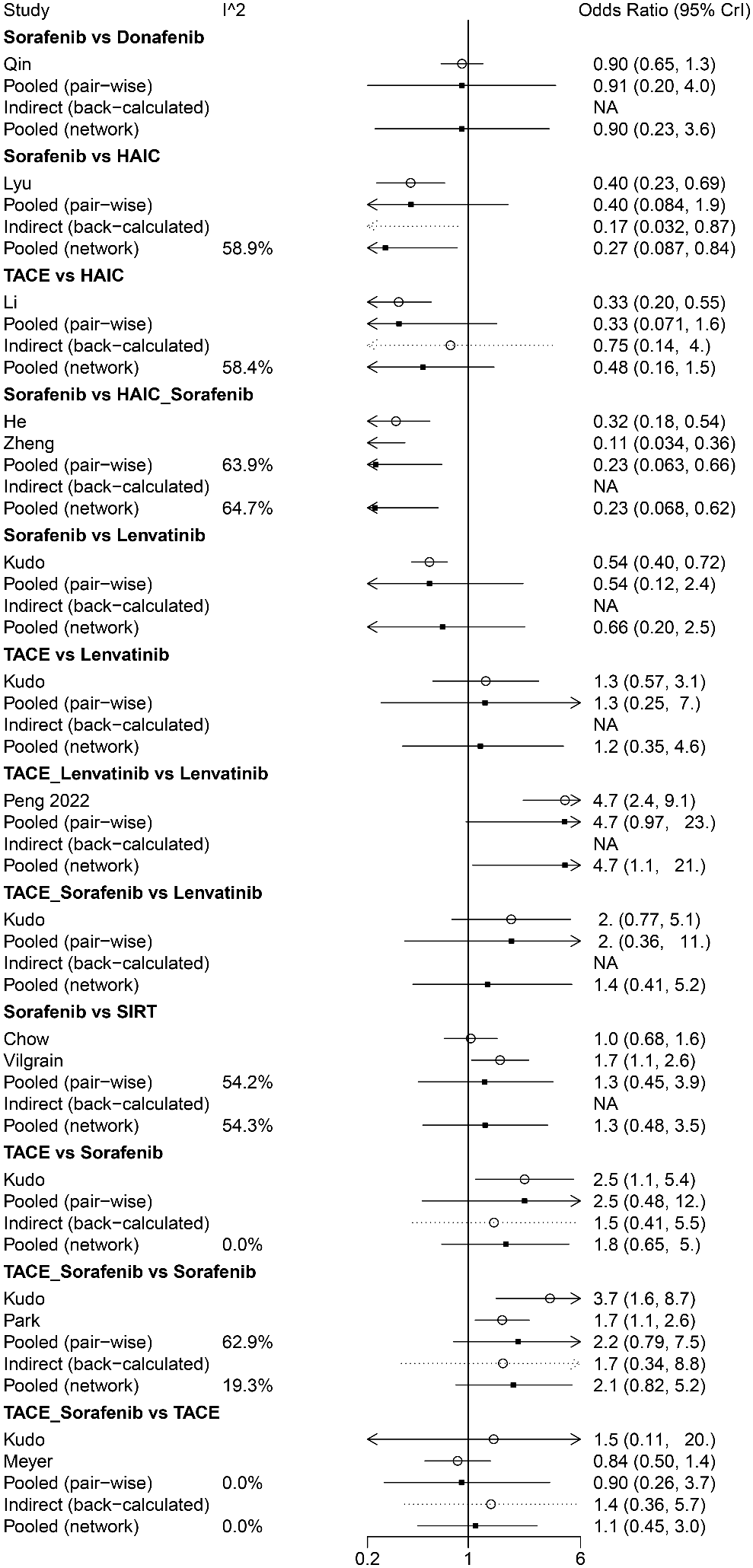


Supplementary Material S12. Forest plot of the outcomes.

PFS
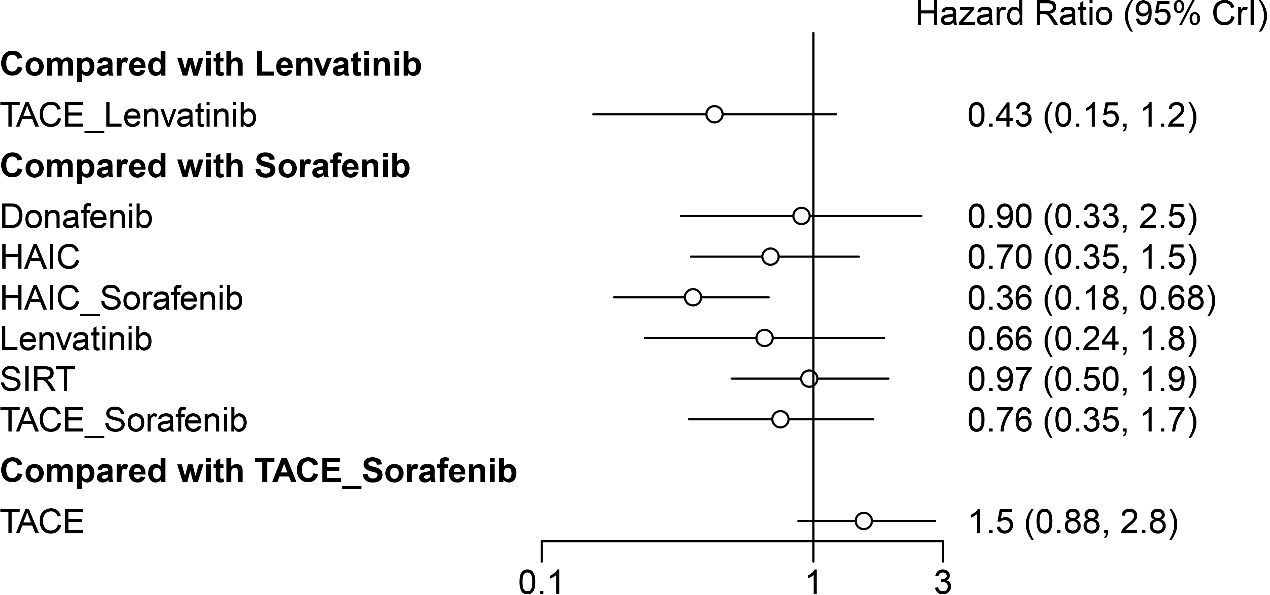


TTP
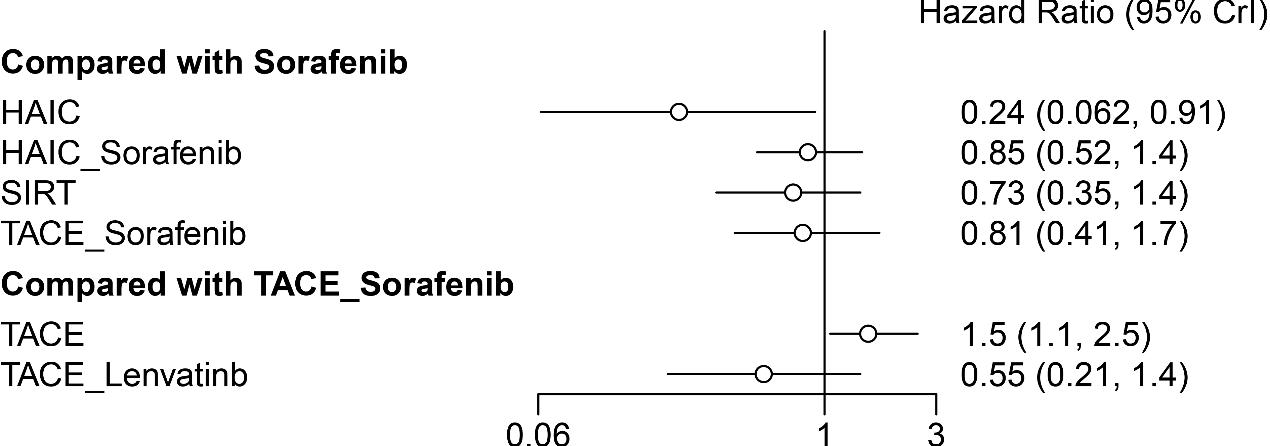


AE
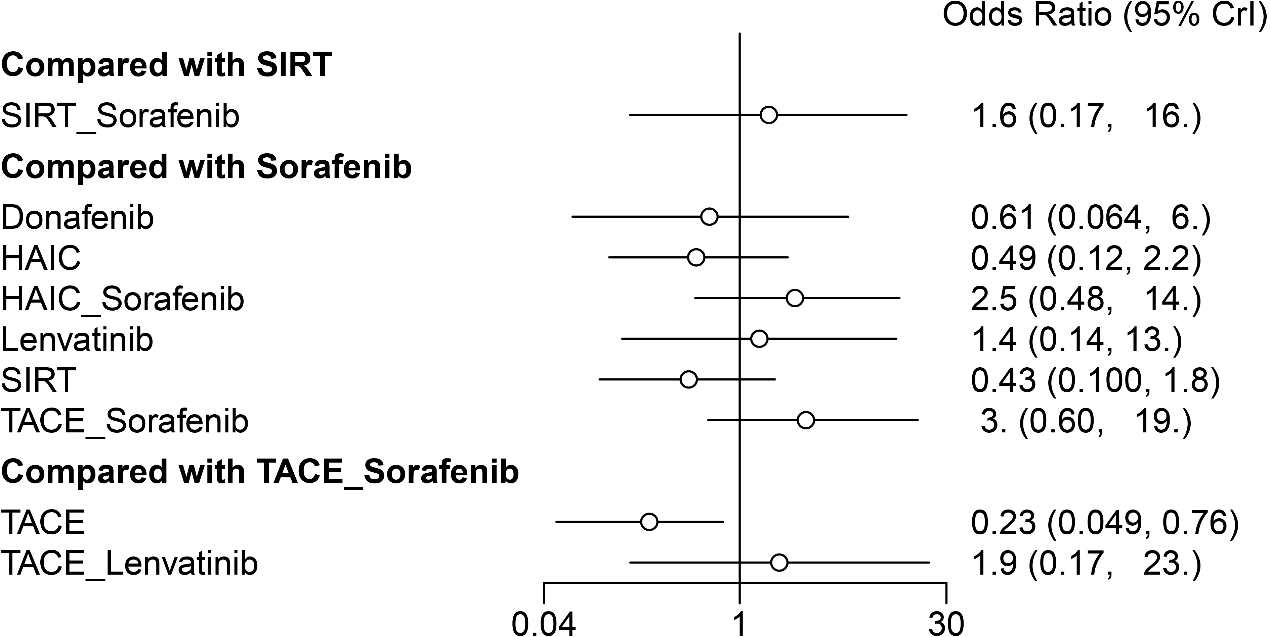


ORR(mRECIST)
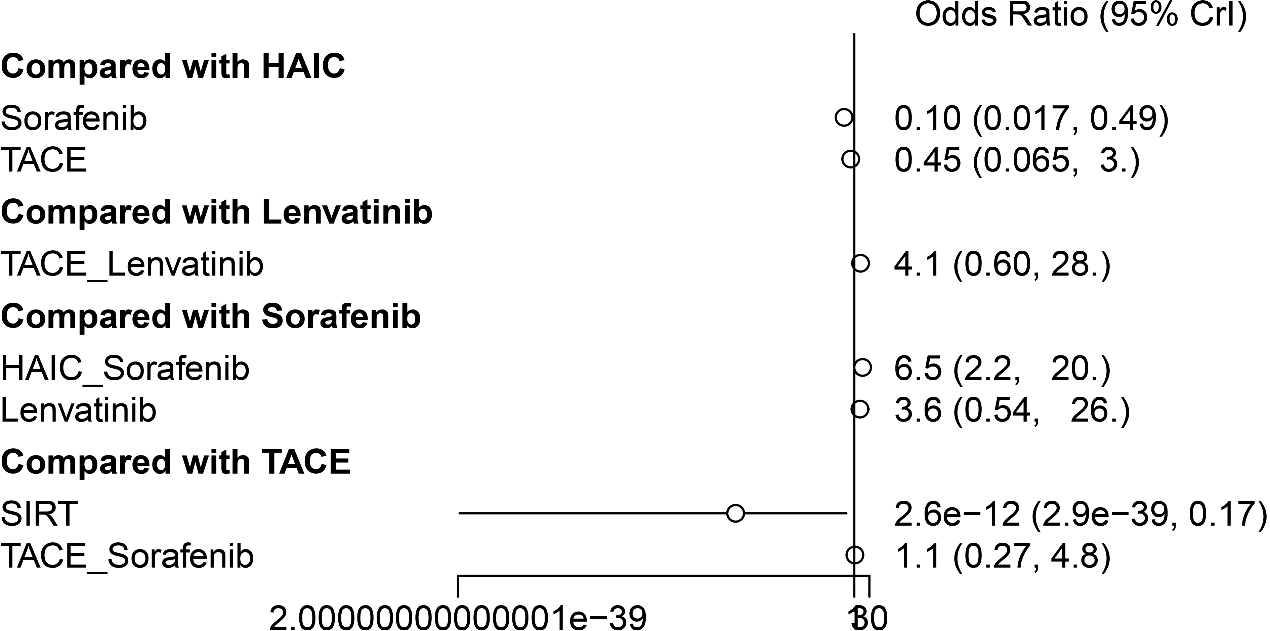


DCR(mRECIST)
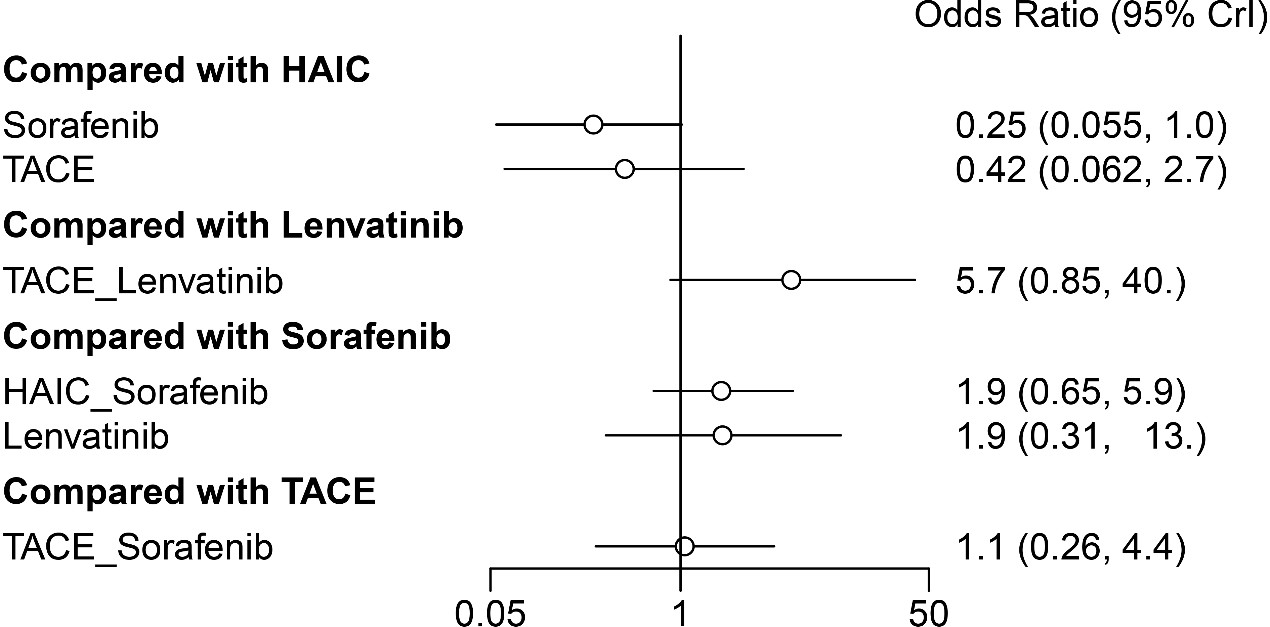


ORR(RECIST)
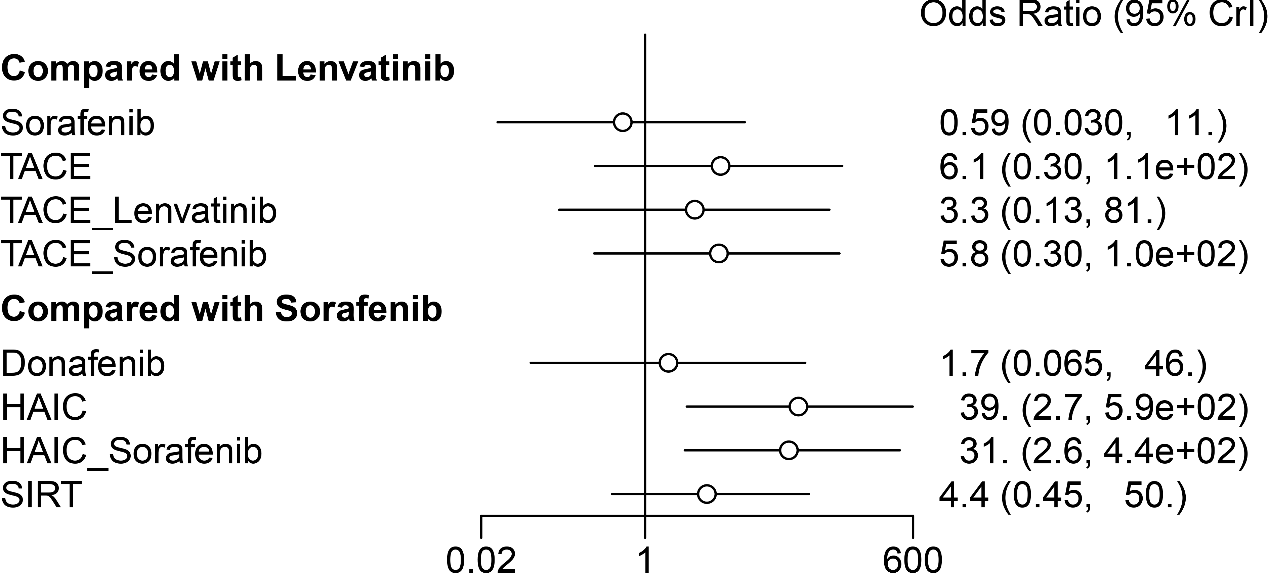


DCR(RECIST)
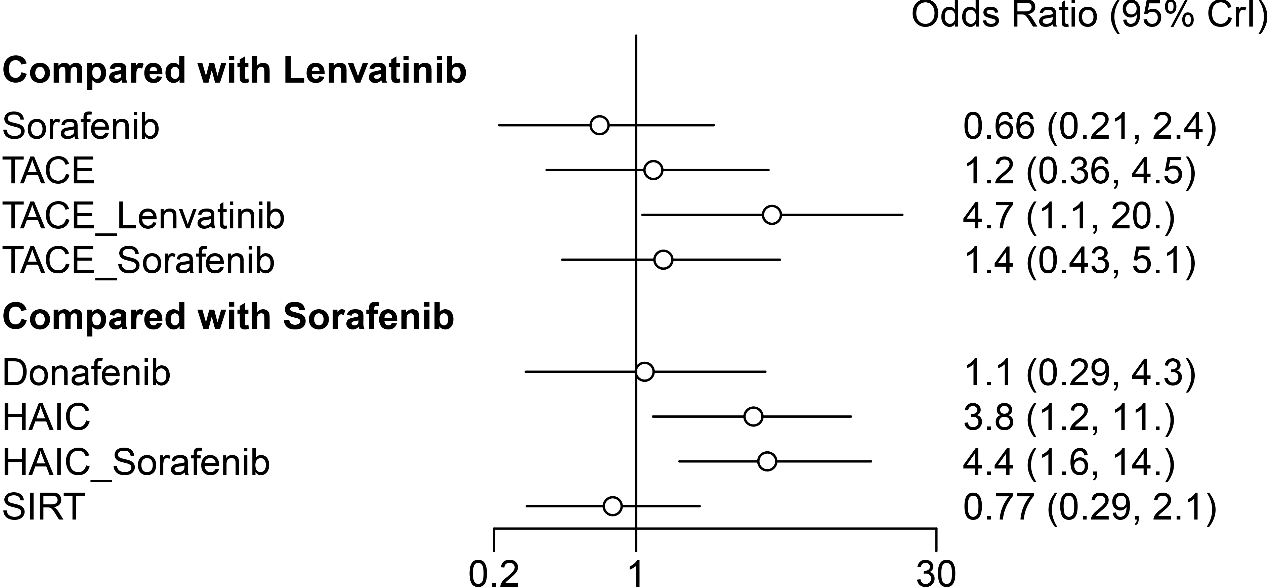


Supplementary Material S13. Forest plot of the outcomes (compared with sorafenib).

PFS
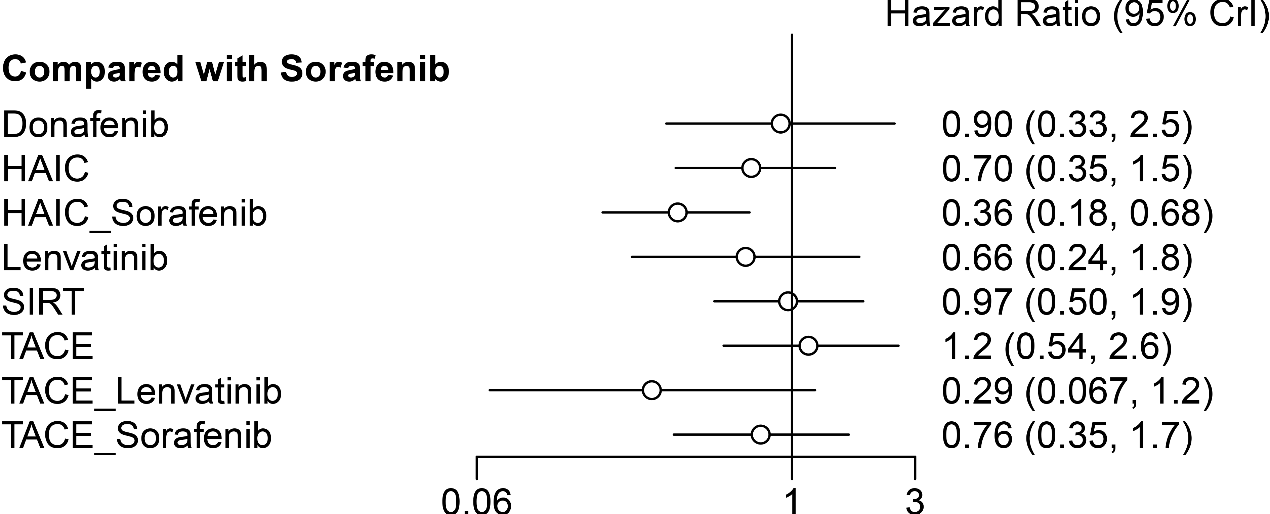


TTP
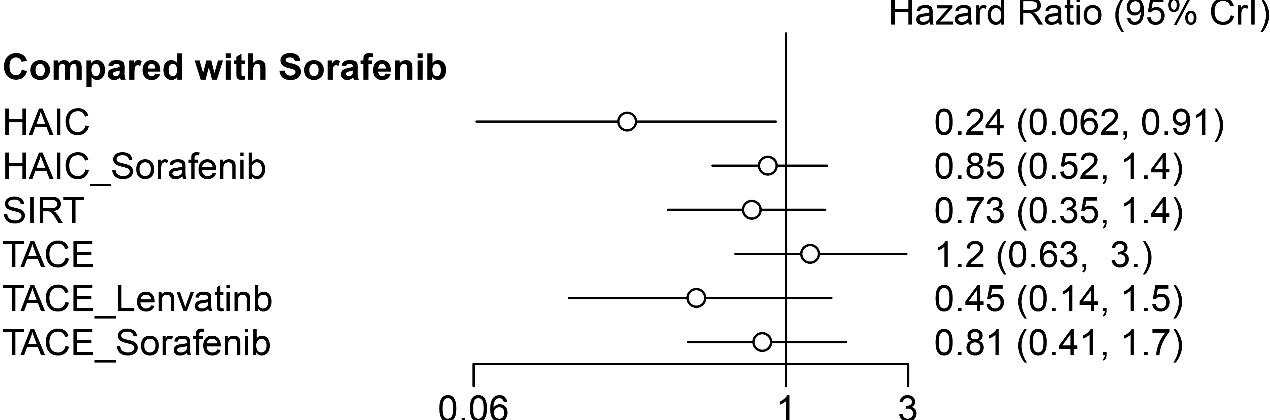


AE
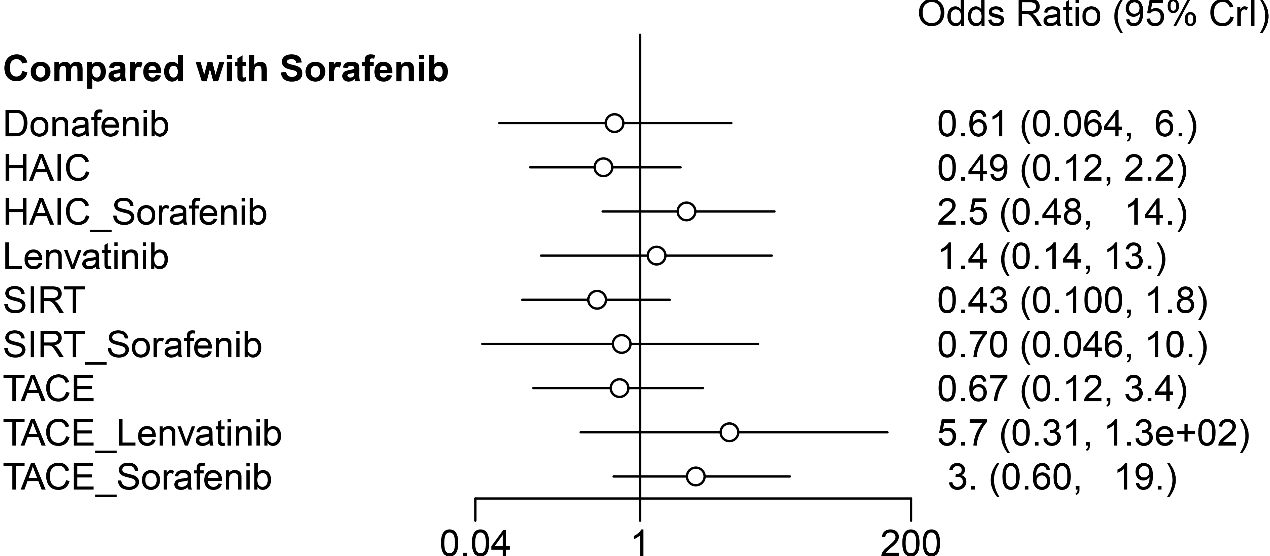


ORR(mRECIST)
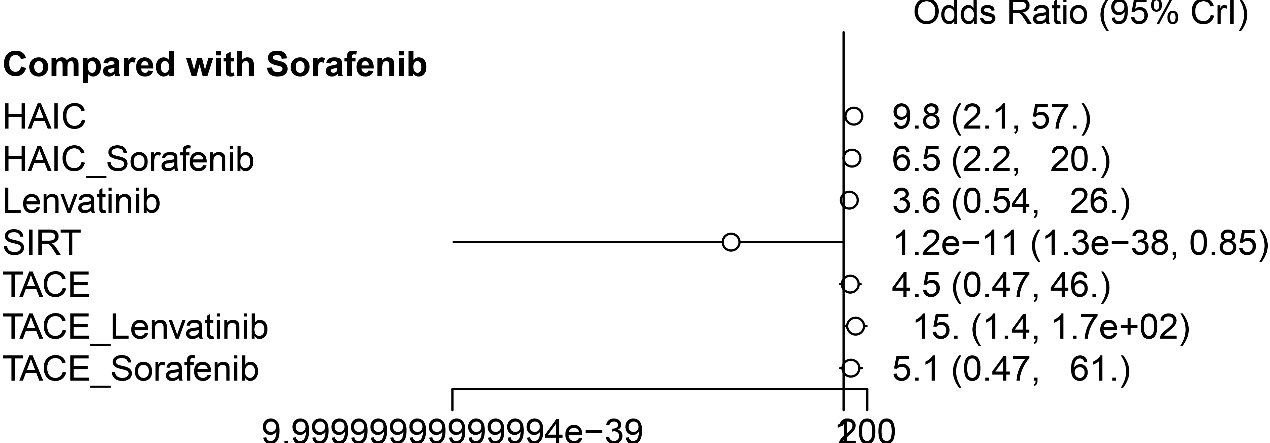


DCR(mRECIST)
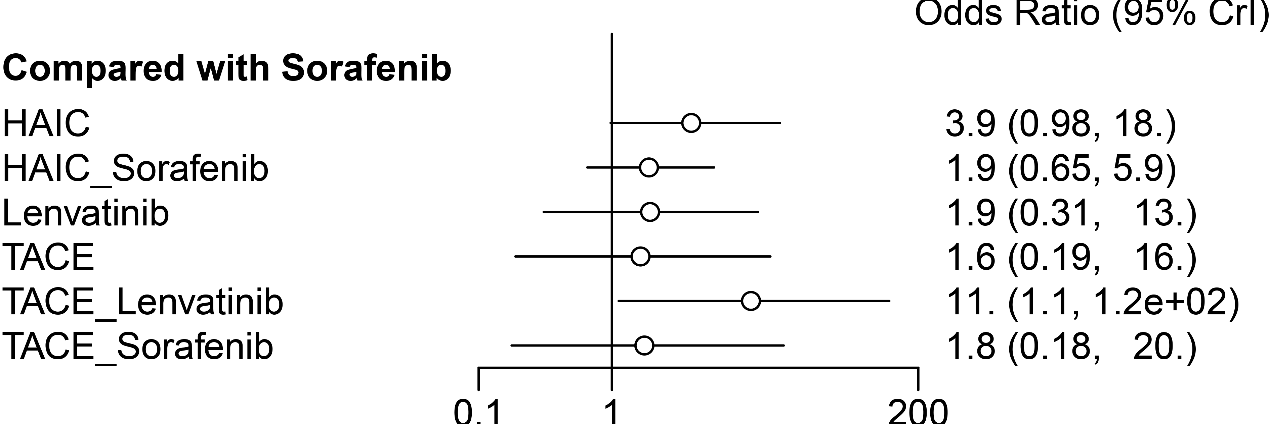


ORR(RECIST)
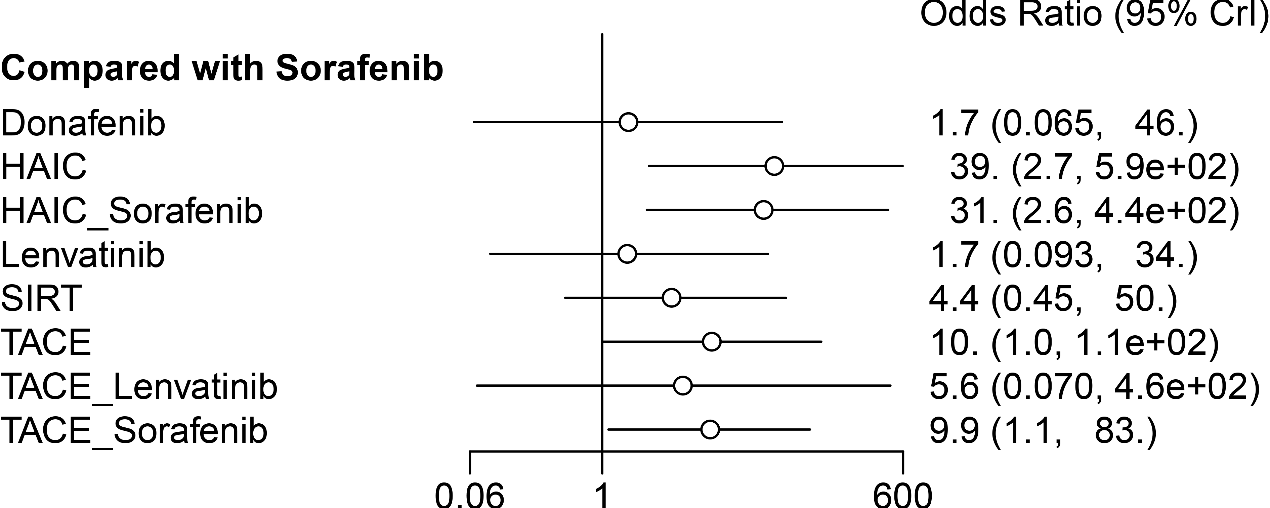


DCR(RECIST)
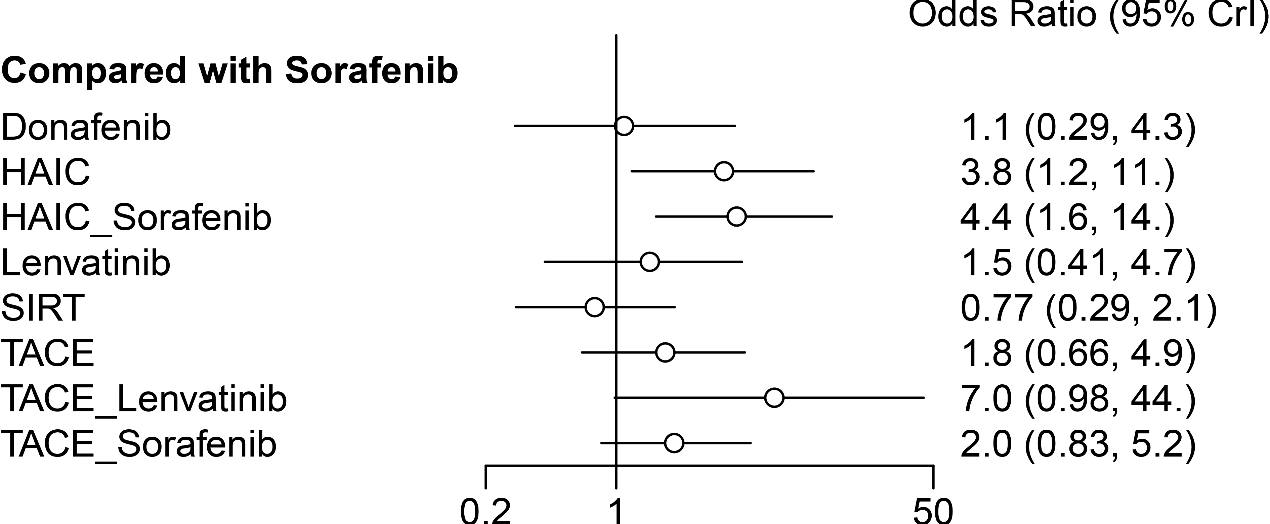


Supplementary Material S14. Results of meta regression for OS.

Covariate:year

1. Empirical mean and standard deviation for each variable,

plus standard error of the mean:

Mean SD Naive SE

d.HAIC.TACE 0.73929 0.3137 0.0011090

d.Lenvatinib.TACE_Lenvatinib -0.68733 0.3591 0.0012697

d.SIRT.SIRT_Sorafenib -0.14879 0.4037 0.0014275

d.Sorafenib.Donafenib 0.01522 0.4220 0.0014919

d.Sorafenib.HAIC -0.66676 0.2896 0.0010240

d.Sorafenib.HAIC_Sorafenib -0.56606 0.2004 0.0007087

d.Sorafenib.Lenvatinib -0.09045 0.3492 0.0012346

d.Sorafenib.SIRT -0.18975 0.2638 0.0009326

d.Sorafenib.TACE_Sorafenib -0.06682 0.2752 0.0009729

sd.d 0.35782 0.1293 0.0004572

B -0.57584 0.4361 0.0015419

Time-series SE

d.HAIC.TACE 0.002920

d.Lenvatinib.TACE_Lenvatinib 0.002018

d.SIRT.SIRT_Sorafenib 0.001594

d.Sorafenib.Donafenib 0.003608

d.Sorafenib.HAIC 0.002761

d.Sorafenib.HAIC_Sorafenib 0.001002

d.Sorafenib.Lenvatinib 0.002048

d.Sorafenib.SIRT 0.001998

d.Sorafenib.TACE_Sorafenib 0.002136

sd.d 0.001718

B 0.008925

2. Quantiles for each variable:

2.5% 25% 50%

d.HAIC.TACE 0.1409 0.5418 0.72854

d.Lenvatinib.TACE_Lenvatinib -1.3983 -0.9110 -0.68974

d.SIRT.SIRT_Sorafenib -0.9589 -0.3907 -0.14892

d.Sorafenib.Donafenib -0.8447 -0.2337 0.02118

d.Sorafenib.HAIC -1.2658 -0.8443 -0.65611

d.Sorafenib.HAIC_Sorafenib -0.9609 -0.6921 -0.56805

d.Sorafenib.Lenvatinib -0.7766 -0.3022 -0.09849

d.Sorafenib.SIRT -0.7268 -0.3487 -0.18758

d.Sorafenib.TACE_Sorafenib -0.6255 -0.2360 -0.06505

sd.d 0.1604 0.2688 0.33908

B -1.4190 -0.8576 -0.58118

75% 97.5%

d.HAIC.TACE 0.92543 1.39606

d.Lenvatinib.TACE_Lenvatinib -0.46868 0.03923

d.SIRT.SIRT_Sorafenib 0.09465 0.66312

d.Sorafenib.Donafenib 0.26884 0.85269

d.Sorafenib.HAIC -0.48068 -0.11600

d.Sorafenib.HAIC_Sorafenib -0.44117 -0.16277

d.Sorafenib.Lenvatinib 0.11539 0.62769

d.Sorafenib.SIRT -0.02793 0.33002

d.Sorafenib.TACE_Sorafenib 0.10234 0.48009

sd.d 0.42574 0.66332

B -0.29701 0.28726

-- Model fit (residual deviance):

Dbar pD DIC

21.931152 -2.384436 19.546716

21 data points, ratio 1.044, I^2 = 9%

-- Regression settings:

Regression on "year", shared coefficients, "Sorafenib" as control

Input standardized: x' = (year - 2019) / 5.585696

Estimates at the centering value: year = 2019

Covariate: sample size

1. Empirical mean and standard deviation for each variable,

plus standard error of the mean:

Mean SD Naive SE

d.HAIC.TACE 0.84926 0.3704 0.0013097

d.Lenvatinib.TACE_Lenvatinib -0.70500 0.4444 0.0015711

d.SIRT.SIRT_Sorafenib -0.14998 0.4634 0.0016385

d.Sorafenib.Donafenib -0.20786 0.6089 0.0021529

d.Sorafenib.HAIC -0.77935 0.3127 0.0011055

d.Sorafenib.HAIC_Sorafenib -0.52720 0.2672 0.0009446

d.Sorafenib.Lenvatinib -0.03379 0.6982 0.0024686

d.Sorafenib.SIRT -0.09271 0.3189 0.0011276

d.Sorafenib.TACE_Sorafenib -0.06908 0.3416 0.0012077

sd.d 0.42399 0.1367 0.0004832

B 0.02442 0.4890 0.0017290

Time-series SE

d.HAIC.TACE 0.005779

d.Lenvatinib.TACE_Lenvatinib 0.008229

d.SIRT.SIRT_Sorafenib 0.001765

d.Sorafenib.Donafenib 0.025591

d.Sorafenib.HAIC 0.002585

d.Sorafenib.HAIC_Sorafenib 0.008503

d.Sorafenib.Lenvatinib 0.038165

d.Sorafenib.SIRT 0.006591

d.Sorafenib.TACE_Sorafenib 0.005814

sd.d 0.001717

B 0.033001

2. Quantiles for each variable:

2.5% 25% 50%

d.HAIC.TACE 0.1153 0.6200 0.84330

d.Lenvatinib.TACE_Lenvatinib -1.6040 -0.9781 -0.70367

d.SIRT.SIRT_Sorafenib -1.0777 -0.4355 -0.15103

d.Sorafenib.Donafenib -1.4083 -0.5903 -0.21204

d.Sorafenib.HAIC -1.4133 -0.9746 -0.77687

d.Sorafenib.HAIC_Sorafenib -1.0680 -0.6931 -0.52465

d.Sorafenib.Lenvatinib -1.4008 -0.4862 -0.04117

d.Sorafenib.SIRT -0.7460 -0.2876 -0.08919

d.Sorafenib.TACE_Sorafenib -0.7472 -0.2831 -0.07044

sd.d 0.2184 0.3288 0.40355

B -0.9538 -0.2803 0.02793

75% 97.5%

d.HAIC.TACE 1.0754 1.6094765

d.Lenvatinib.TACE_Lenvatinib -0.4283 0.1765392

d.SIRT.SIRT_Sorafenib 0.1337 0.7789996

d.Sorafenib.Donafenib 0.1672 1.0313182

d.Sorafenib.HAIC -0.5808 -0.1681849

d.Sorafenib.HAIC_Sorafenib -0.3580 0.0005941

d.Sorafenib.Lenvatinib 0.4086 1.3750450

d.Sorafenib.SIRT 0.1049 0.5388858

d.Sorafenib.TACE_Sorafenib 0.1430 0.6218684

sd.d 0.4948 0.7537662

B 0.3512 0.9667891

-- Model fit (residual deviance):

Dbar pD DIC

21.53874 18.34161 39.88035

21 data points, ratio 1.026, I^2 = 7%

-- Regression settings:

Regression on "sample", shared coefficients, "Sorafenib" as control

Input standardized: x' = (sample - 142.619) / 223.0036

Estimates at the centering value: sample = 142.619

Covariate: mean age

1. Empirical mean and standard deviation for each variable,

plus standard error of the mean:

Mean SD Naive SE

d.HAIC.TACE 0.676147 0.2065 0.0007300

d.Lenvatinib.TACE_Lenvatinib -0.702397 0.2416 0.0008542

d.SIRT.SIRT_Sorafenib -0.147981 0.2572 0.0009093

d.Sorafenib.Donafenib 0.221146 0.2664 0.0009420

d.Sorafenib.HAIC -0.545484 0.1994 0.0007049

d.Sorafenib.HAIC_Sorafenib -0.509876 0.1426 0.0005042

d.Sorafenib.Lenvatinib -0.099321 0.2124 0.0007511

d.Sorafenib.SIRT -0.062655 0.1634 0.0005779

d.Sorafenib.TACE_Sorafenib -0.006308 0.1839 0.0006503

sd.d 0.189924 0.1136 0.0004017

B 0.626581 0.1862 0.0006582

Time-series SE

d.HAIC.TACE 0.002468

d.Lenvatinib.TACE_Lenvatinib 0.002052

d.SIRT.SIRT_Sorafenib 0.001613

d.Sorafenib.Donafenib 0.002289

d.Sorafenib.HAIC 0.002718

d.Sorafenib.HAIC_Sorafenib 0.001487

d.Sorafenib.Lenvatinib 0.001470

d.Sorafenib.SIRT 0.001259

d.Sorafenib.TACE_Sorafenib 0.001807

sd.d 0.002768

B 0.002834

2. Quantiles for each variable:

2.5% 25% 50%

d.HAIC.TACE 0.30181 0.54750 0.66328

d.Lenvatinib.TACE_Lenvatinib -1.16567 -0.85306 -0.70533

d.SIRT.SIRT_Sorafenib -0.67083 -0.29585 -0.14555

d.Sorafenib.Donafenib -0.32791 0.07568 0.22155

d.Sorafenib.HAIC -0.96913 -0.66363 -0.53704

d.Sorafenib.HAIC_Sorafenib -0.80958 -0.59590 -0.50538

d.Sorafenib.Lenvatinib -0.51061 -0.22034 -0.11340

d.Sorafenib.SIRT -0.42380 -0.14948 -0.05214

d.Sorafenib.TACE_Sorafenib -0.36931 -0.11810 -0.01002

sd.d 0.01301 0.11024 0.17713

B 0.25620 0.51281 0.62582

75% 97.5%

d.HAIC.TACE 0.790383 1.1272

d.Lenvatinib.TACE_Lenvatinib -0.559817 -0.2013

d.SIRT.SIRT_Sorafenib 0.002289 0.3646

d.Sorafenib.Donafenib 0.368450 0.7632

d.Sorafenib.HAIC -0.419282 -0.1680

d.Sorafenib.HAIC_Sorafenib -0.418465 -0.2421

d.Sorafenib.Lenvatinib 0.010386 0.3657

d.Sorafenib.SIRT 0.036823 0.2355

d.Sorafenib.TACE_Sorafenib 0.100995 0.3751

sd.d 0.253163 0.4487

B 0.739672 0.9986

-- Model fit (residual deviance):

Dbar pD DIC

22.63258 -42.01071 -19.37813

21 data points, ratio 1.078, I^2 = 12%

-- Regression settings:

Regression on "mean.age", shared coefficients, "Sorafenib" as control

Input standardized: x' = (mean.age - 61.31905) / 12.68458

Estimates at the centering value: mean.age = 61.31905

Covariate: proportion of male

1. Empirical mean and standard deviation for each variable,

plus standard error of the mean:

Mean SD Naive SE

d.HAIC.TACE 0.68284 0.3255 0.0011508

d.Lenvatinib.TACE_Lenvatinib -0.72888 0.3646 0.0012891

d.SIRT.SIRT_Sorafenib -0.15133 0.4083 0.0014434

d.Sorafenib.Donafenib -0.17065 0.3979 0.0014068

d.Sorafenib.HAIC -0.64279 0.2915 0.0010305

d.Sorafenib.HAIC_Sorafenib -0.43857 0.2094 0.0007404

d.Sorafenib.Lenvatinib -0.05089 0.3450 0.0012199

d.Sorafenib.SIRT -0.09155 0.2544 0.0008994

d.Sorafenib.TACE_Sorafenib -0.14872 0.2860 0.0010113

sd.d 0.36770 0.1177 0.0004160

B -0.47758 0.2946 0.0010416

Time-series SE

d.HAIC.TACE 0.003336

d.Lenvatinib.TACE_Lenvatinib 0.002000

d.SIRT.SIRT_Sorafenib 0.001605

d.Sorafenib.Donafenib 0.001479

d.Sorafenib.HAIC 0.002707

d.Sorafenib.HAIC_Sorafenib 0.001315

d.Sorafenib.Lenvatinib 0.001788

d.Sorafenib.SIRT 0.001229

d.Sorafenib.TACE_Sorafenib 0.002319

sd.d 0.001425

B 0.004755

2. Quantiles for each variable:

2.5% 25% 50%

d.HAIC.TACE 0.03717 0.4799 0.68156

d.Lenvatinib.TACE_Lenvatinib -1.45219 -0.9569 -0.73059

d.SIRT.SIRT_Sorafenib -0.97427 -0.4006 -0.14952

d.Sorafenib.Donafenib -0.97304 -0.4116 -0.17099

d.Sorafenib.HAIC -1.21992 -0.8259 -0.64424

d.Sorafenib.HAIC_Sorafenib -0.85547 -0.5704 -0.43876

d.Sorafenib.Lenvatinib -0.74138 -0.2632 -0.05292

d.Sorafenib.SIRT -0.61394 -0.2469 -0.08637

d.Sorafenib.TACE_Sorafenib -0.71216 -0.3278 -0.15096

sd.d 0.19080 0.2865 0.34998

B -1.07144 -0.6631 -0.47326

75% 97.5%

d.HAIC.TACE 0.88417 1.340145

d.Lenvatinib.TACE_Lenvatinib -0.50177 0.004215

d.SIRT.SIRT_Sorafenib 0.10037 0.665913

d.Sorafenib.Donafenib 0.07209 0.625492

d.Sorafenib.HAIC -0.46014 -0.060470

d.Sorafenib.HAIC_Sorafenib -0.30583 -0.024926

d.Sorafenib.Lenvatinib 0.16042 0.646901

d.Sorafenib.SIRT 0.06843 0.402364

d.Sorafenib.TACE_Sorafenib 0.02984 0.425472

sd.d 0.42849 0.647235

B -0.28847 0.098534

-- Model fit (residual deviance):

Dbar pD DIC

20.502423 -1.850761 18.651662

21 data points, ratio 0.9763, I^2 = 2%

-- Regression settings:

Regression on "proportion.of.male", shared coefficients, "Sorafenib" as control

Input standardized: x' = (proportion.of.male - 0.8535421) / 0.093287

Estimates at the centering value: proportion.of.male = 0.8535421

Covariate: proportion of Child A

1. Empirical mean and standard deviation for each variable,

plus standard error of the mean:

Mean SD Naive SE

d.HAIC.TACE 0.91640 0.3693 0.0013056

d.Lenvatinib.TACE_Lenvatinib -0.75089 0.4139 0.0014633

d.SIRT.SIRT_Sorafenib -0.15093 0.4578 0.0016185

d.Sorafenib.Donafenib -0.10163 0.4820 0.0017040

d.Sorafenib.HAIC -0.89695 0.3929 0.0013891

d.Sorafenib.HAIC_Sorafenib -0.52352 0.2228 0.0007876

d.Sorafenib.Lenvatinib 0.05667 0.4098 0.0014488

d.Sorafenib.SIRT -0.12351 0.2962 0.0010473

d.Sorafenib.TACE_Sorafenib -0.10284 0.3217 0.0011373

sd.d 0.41814 0.1364 0.0004823

B -0.19509 0.4083 0.0014436

Time-series SE

d.HAIC.TACE 0.004850

d.Lenvatinib.TACE_Lenvatinib 0.003129

d.SIRT.SIRT_Sorafenib 0.001771

d.Sorafenib.Donafenib 0.005349

d.Sorafenib.HAIC 0.008453

d.Sorafenib.HAIC_Sorafenib 0.001028

d.Sorafenib.Lenvatinib 0.003872

d.Sorafenib.SIRT 0.002496

d.Sorafenib.TACE_Sorafenib 0.003074

sd.d 0.001671

B 0.012439

2. Quantiles for each variable:

2.5% 25% 50%

d.HAIC.TACE 0.2012 0.6845 0.90826

d.Lenvatinib.TACE_Lenvatinib -1.5794 -1.0064 -0.75225

d.SIRT.SIRT_Sorafenib -1.0722 -0.4272 -0.15122

d.Sorafenib.Donafenib -1.0608 -0.3964 -0.10533

d.Sorafenib.HAIC -1.6989 -1.1437 -0.89011

d.Sorafenib.HAIC_Sorafenib -0.9705 -0.6618 -0.52310

d.Sorafenib.Lenvatinib -0.7623 -0.1948 0.05409

d.Sorafenib.SIRT -0.7305 -0.3035 -0.11904

d.Sorafenib.TACE_Sorafenib -0.7421 -0.3034 -0.10352

sd.d 0.2104 0.3233 0.39762

B -1.0312 -0.4513 -0.19039

75% 97.5%

d.HAIC.TACE 1.14147 1.67808

d.Lenvatinib.TACE_Lenvatinib -0.49317 0.07985

d.SIRT.SIRT_Sorafenib 0.12683 0.76316

d.Sorafenib.Donafenib 0.18834 0.88013

d.Sorafenib.HAIC -0.64708 -0.13206

d.Sorafenib.HAIC_Sorafenib -0.38323 -0.08258

d.Sorafenib.Lenvatinib 0.30668 0.88774

d.Sorafenib.SIRT 0.06185 0.45790

d.Sorafenib.TACE_Sorafenib 0.09695 0.53845

sd.d 0.49040 0.74561

B 0.06365 0.61226

-- Model fit (residual deviance):

Dbar pD DIC

21.48895 12.64507 34.13402

21 data points, ratio 1.023, I^2 = 7%

-- Regression settings:

Regression on "proportion.of.child.a", shared coefficients, "Sorafenib" as control

Input standardized: x' = (proportion.of.child.a - 0.8997074) / 0.1968658

Estimates at the centering value: proportion.of.child.a = 0.8997074
